# Inverse Symmetry in Complete Genomes and Whole-Genome Inverse Duplication

**DOI:** 10.1371/journal.pone.0007553

**Published:** 2009-11-09

**Authors:** Sing-Guan Kong, Wen-Lang Fan, Hong-Da Chen, Zi-Ting Hsu, Nengji Zhou, Bo Zheng, Hoong-Chien Lee

**Affiliations:** 1 Graduate Institute of Systems Biology and Bioinformatics, National Central University, Chungli, Taiwan, Republic of China; 2 Department of Physics, National Central University, Chungli, Taiwan, Republic of China; 3 Institute of Modern Physics, Zhejiang University, Hangzhou, Zhejiang, China; 4 National Center for Theoretical Science, Shinchu, Taiwan, Republic of China; Cairo University, Egypt

## Abstract

The cause of symmetry is usually subtle, and its study often leads to a deeper understanding of the bearer of the symmetry. To gain insight into the dynamics driving the growth and evolution of genomes, we conducted a comprehensive study of textual symmetries in 786 complete chromosomes. We focused on symmetry based on our belief that, in spite of their extreme diversity, genomes must share common dynamical principles and mechanisms that drive their growth and evolution, and that the most robust footprints of such dynamics are symmetry related. We found that while complement and reverse symmetries are essentially absent in genomic sequences, inverse–complement plus reverse–symmetry is prevalent in complex patterns in most chromosomes, a vast majority of which have near maximum global inverse symmetry. We also discovered relations that can quantitatively account for the long observed but unexplained phenomenon of 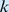-mer skews in genomes. Our results suggest segmental and whole-genome inverse duplications are important mechanisms in genome growth and evolution, probably because they are efficient means by which the genome can exploit its double-stranded structure to enrich its code-inventory.

## Introduction

Symmetry has been considered as an aspect of beauty in mathematics [1], physics [2], chemistry [3], evolution [4], human appearance [5], and psychology [6]. The cause of symmetry is usually subtle, and the pursue of it often leads to a deep understanding of the possessor of the symmetry. Chargaff's parity rule, stating that in a DNA sequence contents of A and T, and of C and G, are separately identical [7], was a crucial clue to Watson and Crick's discovery of the double helical structure of DNA [8]. Chargaff's second parity rule (CPR2) states that at a lower level of accuracy the first rule also extends to a single strand of DNA [9]–[12]. This monomeric base-complement symmetry has two possible generalizations to 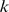-letter words, or 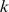-mers: complement and reverse-complement, or inverse, symmetries. It has been suggested that CPR2 is a special case of inverse symmetry, not complement symmetry [13]–[16]. If we represent the four types of bases by black and white arrows that can point up or down–black, A/T; white, C/G; up, A/G; down, C/T–and place a 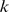-mer in front of a pair of right-angled mirrors, then the laterally reflected image is the reverse (conjugate) of the 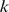-mer, the image reflected through the mirror below, its complement, and the doubly-reflected image through both mirrors, its inverse. For example, the reverse, complement, and inverse conjugates of the 5-mer AAGTC are CTGAA, TTCAG, and GACTT, respectively. In our notion, a genome with a perfect symmetry is one where for every word in the genome, there is symmetry-conjugate of that word somewhere else in the genome. For a proper discussion of symmetry a quantitative description of the phenomenon, including its complete absence, is needed.

In a search for insights into the dynamics that drive the growth and evolution of genomes, we conducted a comprehensive study of the three symmetries in 786 complete chromosomes (all the complete genomes available in public databases when the study was initiated). We focused on symmetry because, in spite of the extreme diversity of genomes, we expect the same dynamical principles and mechanisms to drive genome growth, and expect footprints of the symmetry generating part of the dynamics to be the most robust. For this study we define a new symmetry index, 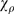, where 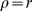, 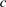, and 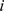 stand for reverse, complement and inverse, respectively, that allows us to quantify accurately the three symmetries in any sequence long enough–about 5 kb–to make word counting in the sequence statistically meaningful. Each symmetry index has a value ranging from zero (perfect symmetry) to approximately unity (absence of symmetry). The value of our index is intuitively understood. For instance, in the case of inverse symmetry, an index value of 0.05 for 5-mers implies that the average difference in the frequencies of all inverse-conjugate pairs of 5-mers is one-twentieth that of all pairs of 5-mers. Using the indexes we verified that all three symmetries are absent as expected in sufficiently long random sequences, and we found that: reverse and complement symmetries are absent, globally and locally, in the 786 complete chromosomes studied; in sharp contrast, a high level of global inverse symmetry (GIS) is ubiquitous in almost all complete chromosomes; the grand average of the GIS index in all complete chromosomes is 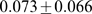; while broadly similar in their global behavior, chromosomes exhibit a wide variety of patterns in local inverse symmetry (LIS); coding and non-coding regions have essentially the same global and local symmetry properties. We infer from these results that inverse segmental duplication, in several forms, is an important mechanism in the growth and evolution of genomes. As a by-product we also gained a quantitative understanding of reverse, complement, and inverse skews in genomes, in monomers and 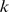-mers.

## Methods

### Ethics Statement

N/A.

### Partition of 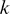-mers into 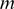-sets

By a genomic sequence we mean a single-stranded sequence. We call a 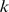-nucleotide word a 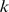-mer and denote the set of all 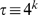 types of 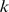-mers by 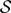. Given a sequence, we count the frequency of occurrence (or frequency) 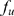 of each 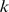-mer type 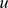 in 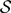 using an overlapping sliding window of width 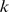 and slide one [17]. The sum of the frequencies is 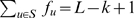, approximated by 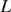, and the mean frequency is 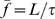. Let the fractional A/T- and C/G-content of a sequence be denoted by 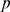 and 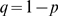, respectively. Whereas the 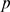 ratio varies widely from genome to genome, the well-verified CPR2 [9], [11] [12] [18] states that in any long stretch of a single strand of genomic sequence the A∶T and C∶G ratios are both invariably close to 1. This property suggests a binary partition of 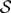 into subsets (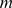-sets) 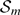, 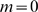 to 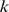, where each of the 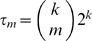 types of 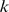-mers in 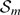 contain 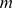 and only 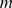 A/T's (note that 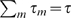) [19]. For example, in the case of 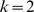, 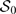 is the set CC, CG, GC, GG; 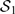 is the set CA, CT, GA, GT, AC, AG, TC, TG; and 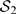 is the set AA AT, TA, TT.

### Definition of Symmetry Indexes

Given 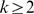, let 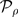 be the set of distinct 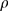-conjugate (but non-self-conjugate) pairs of 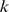-mers types, where 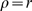, 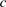, and 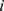 denote reverse, complement, and inverse symmetry, respectively. For example, for 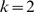, 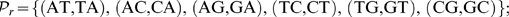; 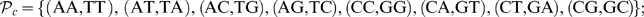; 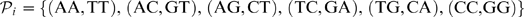. The 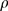-symmetry index, 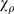, is defined as:
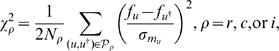
(1)where 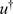 is the 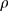-conjugation of 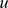, 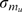 is the standard deviation of the 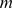-set to which both 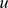 and 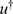 belong, and 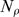 is the number of 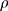-conjugate pairs in 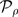. For example, for 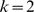, 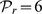, 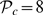, and 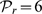. We make two remarks concerning Eq. (1). First, 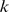-mers from different 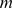 are treated separately. Second, the difference (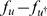) is measured relative to 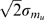, the average *fluctuation* of frequencies of 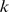-mers within the 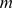-set to which 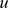 and 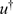 belong. Because the standard deviation in frequencies of all 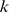-mers (for given 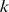, but regardless of 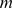-sets) depends sensitively on base composition and is not an accurate measure of the fluctuation in frequencies, the two features mentioned above are crucial for disentangling symmetry from the effects of base composition. By design 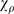 is expected to be close to unity in the absence of 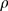-symmetry. A 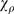 significantly less than unity indicates the presence of 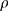-symmetry and 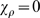 implies exact 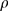-symmetry. We have verified that 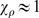 for all three symmetries in random sequences.

### Comparing 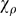 with an 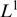-Distance Index

In [15] an index defined in terms of an 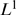-distance, 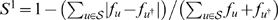, was used to measure symmetries in DNA sequences: the closer 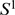 approaches unity the better the symmetry. The weakness of an algorithm based on 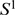 is that it is of the order of unity in *all* cases. Moreover, unlike 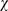 it is sensitive to compositional variations. Table 1 gives the values of 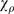 and 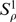 (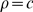 and 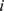) for the 4.6 Mb *E. coli* chromosome, the 228 Mb human chromosom I, and matching random sequences. Here, matching means having the same length and base composition. We obtain a matching random sequence by either sufficiently scrambling the genomic sequence or generating a random sequence using an appropriately loaded die, and have found the two methods yield mutually consistent results as far as symmetry is concerned. It is evident that 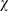 has a significantly better analyzing power. The 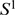 values in the table does indicate inverse symmetry to be *better than* complement symmetry in the *E. coli* genome and the human chromosome I. However, they also indicate both symmetries in random sequences are at least as good as inverse symmetry is in DNA sequences, which is of course incorrect. In sharp contrast, the 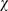 values correctly indicate that inverse symmetry is *present* at a high level in DNA sequences where complement symmetry is *absent*, and both symmetries are *absent* in random sequences. The 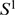 in Table 1 for random sequence is very close to unity not because 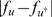 between a conjugate-pair is especially small, but because in a random sequence the difference between any given pair (from the same 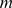-set) is small. This illustrates the importance of measuring 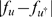 against 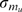, which has non-trivial properties [20].

**Table 1:** Comparing symmetries measured by 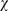 and 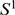.

*k*	Chr.	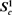	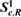	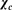	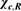	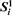	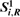	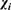	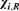
2	*E. coli*	0.9280	0.9992	0.9563	1.1359	0.9974	0.9991	0.0345	1.1925
	HS1	0.8866	0.9998	1.1006	1.1927	0.9992	0.9996	0.0093	1.4425
3	*E. coli*	0.8863	0.9983	0.9607	1.0558	0.9965	0.9982	0.0255	1.0602
	HS1	0.8509	0.9996	0.8821	1.1241	0.9992	0.9996	0.0061	1.1587
4	*E. coli*	0.8323	0.9960	0.9465	1.0086	0.9943	0.9963	0.0307	0.9497
	HS1	0.8001	0.9993	0.8703	1.0733	0.9989	0.9993	0.0065	1.1097
5	*E. coli*	0.7765	0.9918	0.9320	1.0177	0.9905	0.9921	0.0399	0.9706
	HS1	0.7590	0.9988	0.8792	1.0511	0.9984	0.9988	0.0066	1.0207
6	*E. coli*	0.7328	0.9839	0.9188	1.0110	0.9824	0.9846	0.0611	0.9671
	HS1	0.7159	0.9976	0.8888	1.0191	0.9973	0.9976	0.0091	1.0082

The 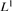-distances (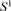) and symmetry indexes (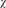) for complement symmetry (subscripts *“c”*) and inverse symmetry (subscript *“I”*) for the *E. coli* genome and the human chromosome 1 and their matching random sequences (subscript *“R”*) are shown. Here matching means having the same length and base composition.

### A Mean-Field Estimate of 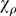


We derive a relation between 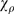 and 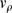, the fraction of 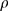-conjugate 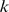-mers that are paired. For simplicity we do not explicitly mention 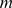-sets and write 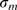 as 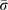, but it is understood that a pair of 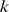-mers always implies a pair in which both 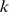-mers belong to the same 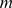-set. Let 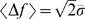 be the average frequency difference of an unrelated pair (of 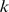-mers). This means a typical pair of 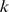-mers have respective frequencies 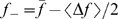 and 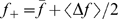, so that on average the fraction of unrelated 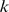-mers that are “paired-up” is
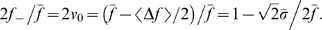
(2)


Similarly, if 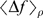 is the average frequency difference in a 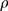-conjugate pair, then the fraction of 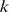-mers paired with their respective 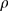-conjugate partners is
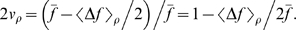
(3)


It follows from the definition of 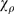 (Eq. (1)) that a mean-field approximation of its value is
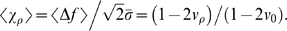
(4)


Note that 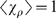 when 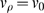 and 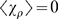 when 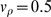. It is important to realize that the value of 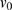 alone does not determine the level of symmetry. For long random sequences, 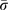 scales as 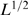 so that 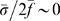 and 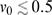. Yet none of three symmetries are present in random sequences, nor are they expected (see Table 1). Finally, the fraction of 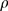-conjugate-paired 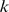-mers above background level is
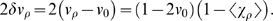
(5)


### Global and Segmental Symmetry Index

**Figure 1:**
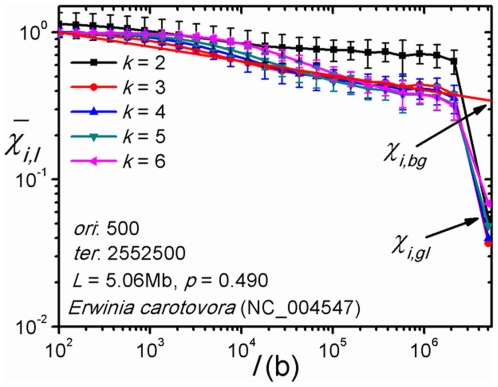
A 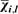 *vs.* segments length (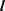) plot. The 5.06 Mb chromosome of *E. carotovora* is partitioned into non-overlapping segments of length 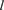 and the 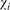 averaged over segments, or 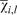, is plotted against 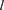. The last datum at full chromosome length gives the global value for 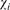, or 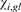. The body of data (for each 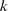) follows an approximate power law (blue line). The value of 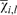 at full length given by the power law (averaged over 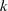) is the background value for 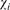, or 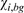. The symbols 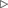 and 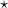, indicate the values of 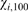 at the original (*ori*) and terminal (*ter*) sites of replication, respectively.

We use 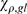 to denote the global 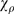 for a whole chromosome and 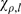 to denote the index for a segment of length 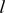. For each chromosome we compute a 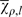 versus 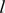 plot such as the one shown in Fig. 1, where each data is the mean segmental value 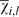 (in this case 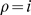) of all the non-overlapping segments of length 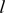 into which the chromosome is partitioned. The error bar gives the standard deviation. The datum at full length is the global 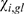 for the chromosome. The body of data is seen to be roughly linear in the log-log plot for segment lengths up to near the full chromosome length, followed by a sharp drop in 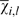 thereafter. There is a measurable 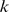-dependence in the data (here 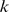 = 2 stands out, but not in all cases) but in this report we consider mostly 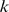-averaged data. We utilize this property to characterize a chromosome by 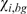 and 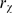, where 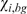 is the linear part of the 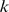-averaged 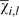 extrapolated to full chromosome length, and 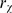 is the ratio 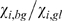. For example, in Fig. 1, 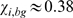, 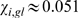, and 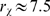.

### The 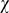-Matrix

Given a chromosome, a user defined overlapping sliding window is used to generate a set of 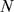 overlapping segments of length 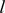 covering the entire chromosome. The (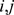) element of the symmetric 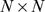 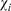-matrix is the 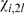 value of the concatenation (of length 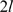) of the 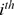 and 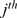 segments of the set.

### Symmetry Index for Coding and Non-Coding Parts

From each complete sequence, the coding and noncoding segments are spliced from a single strand of the chromosome and the segments–coding and noncoding–are separately concatenated in the order and orientation as they occur in the strand to form two sequences, the coding and non-coding parts, respectively. Symmetry indexes for the two parts are separately computed.

### The Complete Sequences

The 786 complete sequences analyzed in this study, 356 eubacteria chromosomes, 28 archaea chromosomes, and 402 chromosomes from 28 eukaryotes, were downloaded in November of 2006 from the National Center for Biotechnology Information (NCBI) chromosome database [21], except the rice genome, which was taken from the Rice Annotation Project Database (RAP-DB) [22]. The set included all the non-redundant prokaryotic and eukaryotic complete genomes in public databases at the time of the download. Individual chromosomes range in length from 200 kb to 230 Mb. The total length of the 786 sequences is 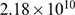 bases. A list of the complete chromosomes is given in Table S1, 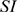.

### Computing Programs

All computing programs used in generating the results reported in this paper can be downloaded from the ISDB [23].

## Results

### The Inverse Symmetry Database (ISDB)

Data, in the form of numerical lists and plots, on local and global symmetries for 786 complete chromosomes are given in the Inverse Symmetry Database (ISDB) [23]. Here we present a summary; results for individual chromosomes are given for illustrative purposes. Some Tables and Figures mentioned but not shown in the text are given in Supporting Information (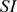).

### Reverse and Complement Symmetries Are Absent on All Scales

**Figure 2:**
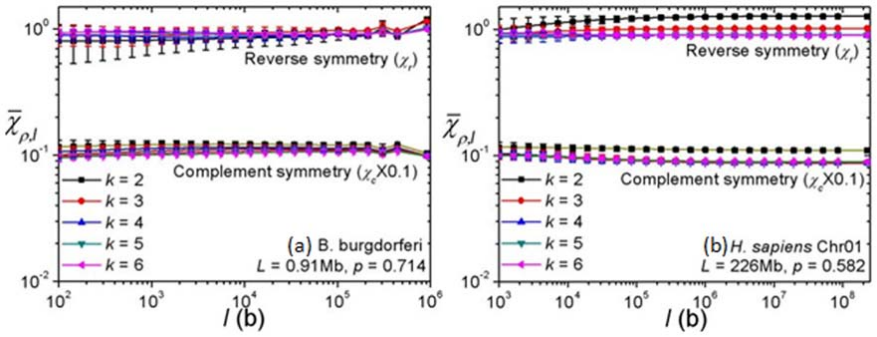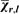 and 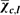 *vs.* segment length plots. (a) *B. burgdorferi* and (b) human chromosome 1. It is seen that 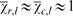 for all lengths.

**Figure 3:**
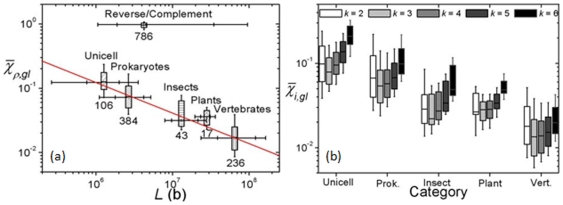
Summary of genome symmetry. (a) At top of plot, (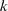-averaged) global 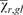 and 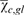 averaged over all chromosomes. Numeral attached to box indicates number of chromosomes in category. Boxes in lower half: global 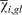 averaged over categories of organisms. Horizontal tics on boxes give 10, 25, 75 and 90 percentile values of lengths of chromosomes in each category. Straight line shows approximate power-law of data. (b) 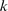-specific 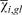 averaged over categories of organisms.

The quantity 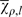 measures the segmental average of the 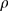-symmetry index for a chromosome partitioned into segments of length 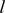 (Methods), and a 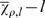-plot reveals the scale-dependence of 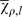. We computed the 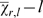 and 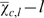 plots for a large selection of chromosomes and found that in all cases the two symmetries were absent on all scales. Two examples, for *B. burgdorferi* and the human chromosome 1, are shown in Fig. 2. The data given at the top of Fig. 3 (a) is a summary of the finding that reverse and complement symmetries are globally absent, 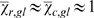, in all chromosomes studied (see ISDB [23] for full results). These results confirm a previous finding [13] that CPR2 cannot be a specialization to monomers of a general 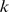-mer complement symmetry.

### All Chromosomes Have Good Global Inverse Symmetry

Fig. 3 summarizes our finding of GIS in the 786 complete chromosomes. The lower part of panel (a) are the result for 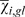 averaged over 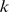 (2 to 6) and category of organisms (Table S1, 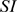) showing that GIS is strongly present, namely 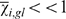. Fig. 3 (b) shows the 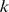-dependence of category-averaged 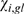 varies with category, possibly as a reflection of the diversity of organisms included in each category. For example, the vertebrates are phylogenetically far closer than the organisms included in the unicellular category. Because the 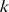-dependence is not pronounced and owing to the large quantity of data, in this report we will focus on 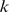-averaged results. Data for eubacteria and archaea are not given separately as they are not significantly different. A power-law dependence on sequence length 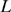, 
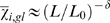
, is evident, where 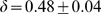 and 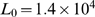 b. The grand average for 786 sequences is 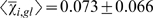. A possible mathematical origin of the power-law behavior will be reported elsewhere. Complete lists of 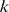-averaged (k = 2 to 6) and 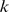-specific 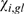 are given in Table S1, 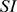 and ISDB [23], respectively.

### The Case of *E. coli* as an Example

The 4.6 Mb genome of *E. coli* 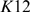 is almost compositionally even with 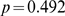. We examine the results for 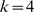. The mean frequency (in bases) of 4-mers is 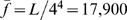 (to three significant figures). The standard deviations for the 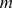-sets (Methods) are computed to be 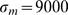, 7660, 5510, 5950, 6050 for 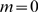 to 4, respectively. The weighted mean standard deviation is 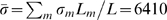, hence the average difference between the frequencies of a typical pair of 4-mers (among the 32640 pairs) is 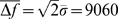. There are 120, 128, and 120 reverse-, complement-, and inverse-conjugate pairs of 4-mers. The global symmetry indexes for the genome are computed to be 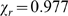, 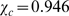, and 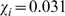. The average frequency differences for a typical pair of reverse, complement, and inverse-conjugate pairs of 4-mers are 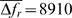, 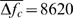, and 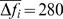, respectively. We remark that 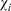 is well approximated by a mean-field estimation of 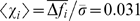 (Methods). This implies, for instance, that if the frequency of the 4-mer AAGC is 18000, then the frequencies of its reverse-conjugate (CGAA), complement-conjugate (TTCG) and inverse-conjugate (GCTT) would fall within the ranges 9000–27000, 9400–26400, and 17700–18300, respectively, with 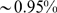 confidence. Note that a single inverse-conjugate pair having a frequency difference of the order of 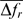 is sufficient to cause 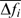 to increase from 280 to 860 and raise 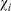 from 0.031 to 0.095.

### Chromosomes Exhibit Several Types of LIS

**Figure 4:**
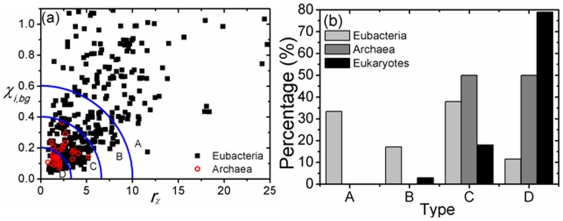
Distributions of genomes on 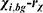 plane and by type. (a) Distribution of prokaryotic chromosomes in the 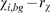 plane. Types delineated by concentric curves, from center outwards, are D, C, B, and A, respectively. (b) Percentage type-distribution of all chromosomes studied.

The 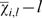 plots (Fig. 1, Methods) of chromosomes exhibit considerable variation (see ISDB [23] for a complete set of such plots). We notice that a 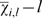 plot can be meaningfully characterized by two quantities: 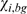, which measures the ambient, or background, LIS, and 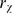, the ratio 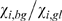. A large 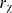 implies the symmetry is much stronger globally than it is locally. Fig. 4 (a) is a 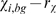 plot of the 786 sequences. Although the data do not appear to form distinct clusters, for ease of discussion we used the function 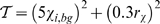 to partition chromosomes into four types: Type A, 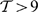; type B, 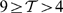; type C, 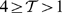; type D, 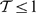. This way of classification implies that whereas the difference between two adjacent chromosomes on opposite sides of a boundary may be fuzzy, there is a stark distinction between, say, type A and type D chromosomes. Of the 356 eubacteria, 33%, 17%, 38%, and 12% are types A, B, C, and D, respectively. The 28 archaeons are split evenly between types C and D, with types A and B absent. About 4%, 21% and 75% of the 402 eukaryotic chromosomes are type B, C and D, respectively (Fig. 4 (b)). A classification of chromosomes by inverse symmetry type is given in Table S2, 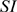. Many of the phylogenetically most deeply rooted thermophilic eubacteria, including *A. aeolicus* and *T. maritima*, are type D. Multicellular chromosomes, with chromosomes larger than the typical bacterial ones, are exclusively type D, but some smaller protozoan chromosomes, including some from *P. falciparum* and *E. cuniculi*, are type B or C (Figure S1, 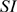). With few exceptions inter-chromosomal differences in multicellular organisms are slight, while those in protozoans tend to be larger. Within a complete sequence, the general properties in inverse symmetry of coding and non-coding parts are similar (Figure S2, 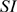). We note that CPR2 is significantly more strongly violated in individual genes (exons in eukaryotes) than in non-coding regions (see, e.g., [24]). However, this difference is not apparent in our case because of the way we concatenate coding (and non-coding) parts. Specifically, the coding part concatenates all genes in both orientations into a single strand (Methods). Several factors that generally hold–there are exceptions–now conspire to make CPR2 violation on a long stretch of the coding concatenate to be typically much weaker than that in a single gene: the level of violation is fairly uniform for all genes; the densities of positively (+) and negatively (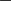) oriented genes are about the same; the violations on +genes and 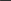
genes have opposite signs. The vast majority of chromosomes have 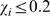; see Table S3, 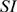 for a list of 38 exceptional chromosomes. Of the 23 prokaryotic chromosomes in this category, 10 are type A, 6 each are types B and C, and 1 is type D; all are eubacteria. Of the eukaryotic chromosomes in this category, 9 and 6 each are types B and C, respectively; all are unicellular and most are from the yeast, *P. falciparum*, and *E. cuniculi* genomes. Only one chromosome has 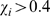: *X. fastidiosa*, with 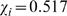. A preliminary study indicates a correlation between type-classification and phylogeny [25].

### 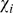-Matrix Reveals Strong Intra-Chromosomal Correlation in Segmental Inverse Symmetry

**Figure 5:**
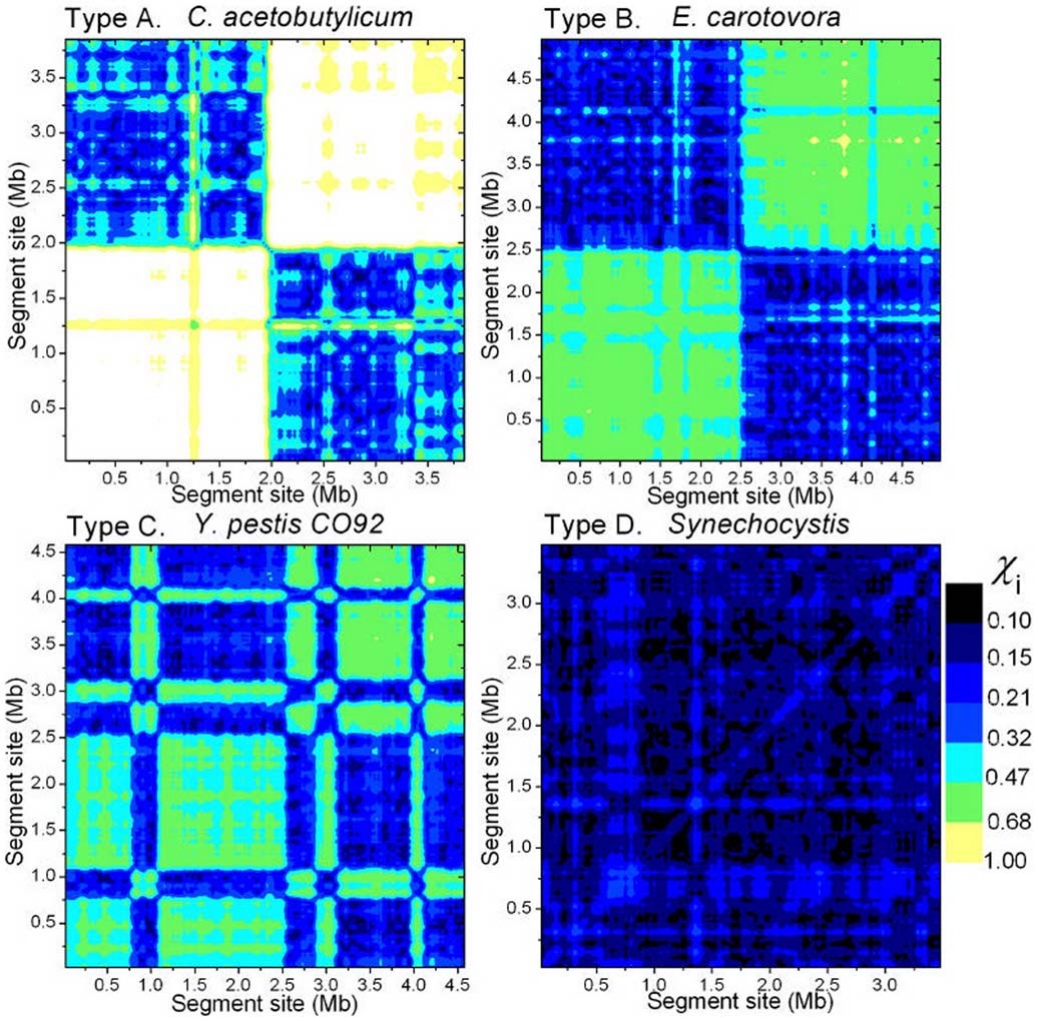
Four typical 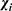-matrix plots. 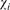
-matrix plots (Methods) for four type-representative chromosomes: *C. acetobutylicum*, type A; *E. carotovora*, type B; *M. mazei*, type C; *Synechocystis*, type D. The window size is 100 kb and the slide is 25 kb. Each 25 kb by 25 kb pixel shows the color-coded, 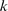-averaged (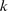 = 2 to 5) value 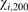 of a 200 kb concatenate composed of two 100 kb taken from the 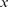 th and 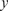 th windows of the chromosome. The diagonal of a 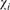-matrix is mapped to the skew-diagonal of the corresponding 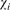-matrix plot. The color code is linear in 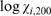.

The 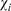-matrix is designed to display the inverse relation between two segments from the same chromosome. In the present case, a chromosome is scanned by a window of width 100 kb and slide 25 kb, and the (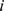, 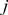)-element of a 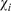-matrix gives the 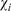-value for the 200 kb concatenate composed of the 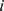 th and 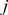 th (100 kb) segments in the chromosome (Methods). By definition a 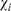-matrix is symmetric. Fig. 5 shows graphical representations of 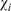-matrices, or 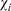-matrix plots, of four representative chromosomes, *C. acetobutylicum*, *E. carotovora*, *M. mazei*, and *Synechocystis*, one for each of the four types. The chromosomes were chosen for their typicality, for having 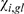
's being approximately 0.05, and for having lengths approximately 4 Mb.

We first focus on the 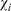-matrix plot for the type-A *C. acetobutylicum*, Fig. 5 (a). It is composed of four roughly equal-sized quadrants (the plot is symmetric with respect to its skew diagonal). With the finer structure ignored, the two diagonal quadrants are “gray/blue”, or 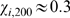, and the two skew-diagonal quadrants are white, or 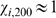. An exception is a single pixel near the midpoint of the skew-diagonal, which is light gray/blue. The whiteness of the skew-diagonal (except the midpoint) implies LIS on a scale of 100 kb is absent in the entire chromosome. For reference, the 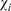-matrix plot for a random sequence will be all white. The midpoint of the chromosome happens to be near the terminal site (*ter*) of replication. We call the (approximate) half-chromosome to the left of *ter* the lead-strand, and the other half the lag-strand. The (200 kb) concatenates whose 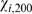 make up the lower-left (upper-right, respectively) quadrant are composed of segments both from the lead-strand (lag-strand), and those whose 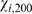 make up the upper-left quadrant (or the lower-right, which is the same) are composed of one segment from the lead-strand and another from the lag-strand. The light color of the two skew-diagonal quadrants implies that the 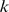-mer contents of any two segments from the same strand are similar, such that the inverse-symmetry property of the concatenate is close to that of either of the component segments, which in this case have similar low levels of symmetry. In contrast, and this is the most interesting part of the plot, the dark color of the two diagonal quadrants suggests that any two segments taken from different strands–one each from the lead and lag strands–have a significant “inverse relation”, meaning that, as far as 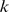-mer content is concerned, the two segments are relatively close to being mutual inverse conjugates. This being the case, the nature of the non-whiteness of the single pixel near the middle of the skew-diagonal is also understood: it indicates a (200 kb) concatenate that straddles the *ter* site, so that one of its component segments is (mostly) from the lead-strand, and the other from the lag-strand. We can now interpret the entire pattern of the type-A 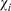-matrix plot as follows: the chromosome is bisected by the *ter* site into two almost equal strands (in some cases the bisection occurs at the origin site (*ori*) and in some cases the partition is not so nearly equal), each of which is without inverse symmetry, but 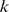-mer-wise the two are nearly mutual inverse conjugates. However, it cannot be said that the two strands are simply mutual mirror inverse copies. For if that were the case, then the two diagonal quadrants would be mostly white save for a black, narrow diagonal ridge several pixels wide. In the type-A 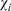-matrix plot shown in Fig. 5, which is that of *C. acetobutylicum*, the neat four-quadrant appearance reflects the fact that the *ori* site is close to the origin of the genome. In some type-A chromosomes neither the *ori* nor the *ter* site is close to the origin, and this causes their 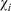-matrix plots to look superficially more complicated (see below).

The 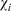-matrix plot for the type-B chromosome is similar to a type-A plot, except that all four quadrants are a shade darker than their counterparts in the type-A plot, caused by the chromosome having a higher (and nearly) homogeneous ambient inverse symmetry with 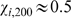. The type-D plot is qualitatively different from the two just discussed. It does not have a quadrant structure and therefore exhibits no hint of bisection of the chromosome. Rather, not counting finer structures, it shows 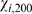 to be 0.2 or less everywhere, implying that the inverse relation between any two segments is strong. The type-C chromosome has a structure intermediate between types B and D. The example shown in Fig. 5 is broadly composed of two sections: a type-B-like section from 0 to 2.7 Mb and a type-D-like section from 2.7 to 4 Mb. In this context, the 0.9 to 3.8 Mb segment of the type-D *Synechocystis* appears to be a “super-type D” embedded within the chromosome. Fig. 5 (a–c) show that some segments within a chromosome, some as long as 1 Mb, have LIS significantly distinct from that of the rest of the chromosome. Such segments suggest alien origins, possibly the result of lateral gene transfers [26].

**Figure 6:**
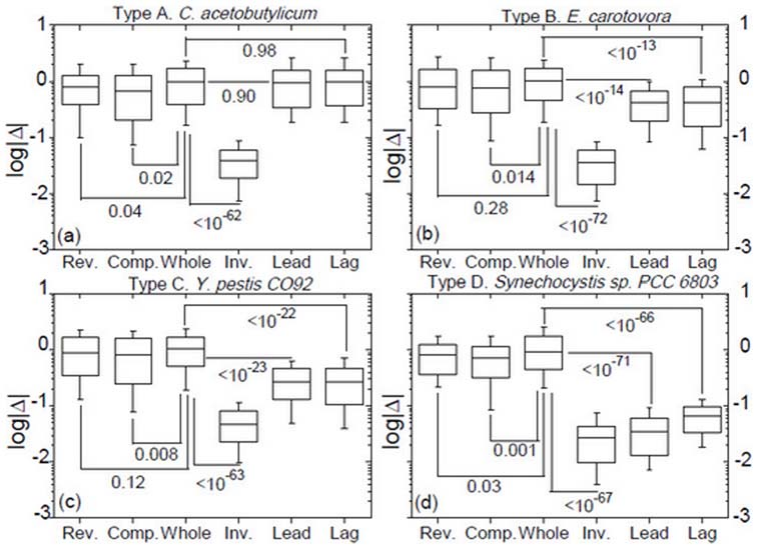
Type-specificity of 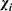-matrix plots. Statistics and P-values of distributions of 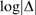 for 4-mers in the four type-representative chromosomes. The “rev”, “comp”, and “inv” sets of 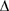
's are the terms 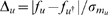 for conjugate-pairs (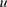,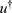) contributing to 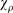, 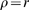, 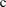, and 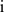, respectively (Eq. (1)). The numbers of r, c, and i-conjugate pairs of 4-mers are 120, 128, and 120, respectively. The “whole” set consists of 1000 randomly selected pairs from a total of 8484, where in each pair both 4-mers belong to the same 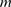-set (Methods) but are otherwise unrelated. In “lead” and “lag”, pairs are 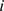-conjugates from the lead-strand and lag-strand, respectively.

The type-specificity of 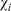-matrix plots is also seen in the box plots in Fig. 6, which compare distributions of frequency differences (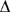) of pair of 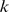-mers (in this case 4-mers) in various pair-sets. For type A the set of 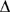
's for intra-strand inverse-conjugate pairs (lead and lag) are not different from that for uncorrelated pairs (whole), while that for all inverse-conjugate pairs (inv) has distinctly smaller values. In contrast, for type D the lead- and lag-sets are similar to the inv-set and have distinctly smaller values than the whole-set. As before the patterns for types B and C are intermediate between type A and type D.

**Figure 7:**
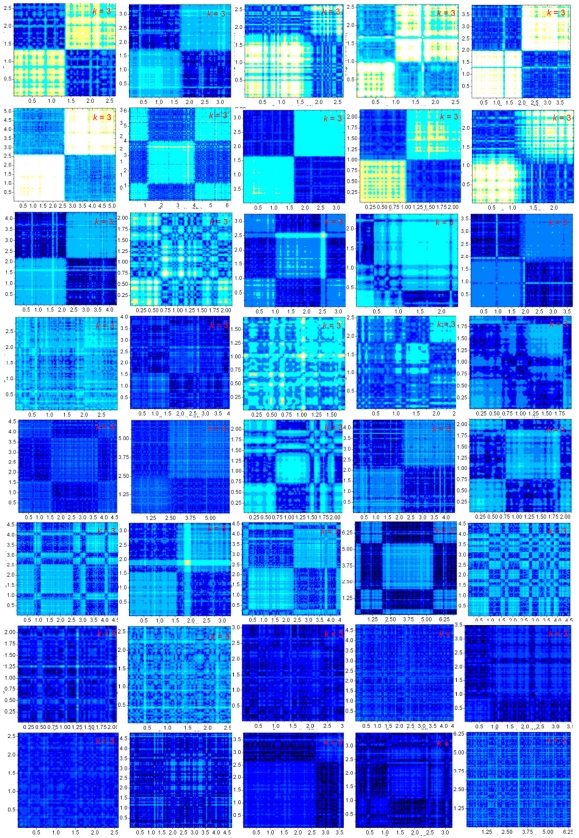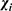-matrix plots of 40 prokaryotic chromosomes. Rows 1 and 2:, type-A eubacteria; row 3, type-B eubacteria; row 4, type-C archaea; rows 5 and 6, type-C eubacteria; row 7, type-D archaea; row 8, type-D eubacteria. The chromosomes are all longer than 2 Mb and are otherwise selected to represent the breath of the variety of the plots.

In Fig. 7 the 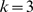 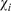-matrix plots of 40 prokaryotic chromosomes are shown to indicate the diversity of chromosomes as reflected in such plots. The types of the eight rows are, respectively: A (eubacteria), A (eub), B (eub), C (archaea), C (eub), C (eub), D (arc), D (eub). We remark that in some plots a tidier four-quadrant structure can be obtained by a shifting of the coordinates of the origin. The general trend of type-dependence of the plots is such that, going from A to D, the largest structure decreases in size, and the lightest color gets darker, or equivalently, the ambient level of inverse symmetry rises. All plots are rich in fine structure and invite a deeper level of analysis than presented here. The 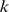-specific 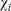-matrix plots of all 384 prokaryotic chromosomes are given in ISDB [23]. 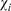-matrix plots of eukaryotic chromosomes, which generally take longer to compute, will be added to the database at a later date.

### Four-Quadrant Structure of 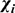-Matrix Plot Is Directly Related to Discontinuity in 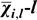 Plot

**Figure 8:**
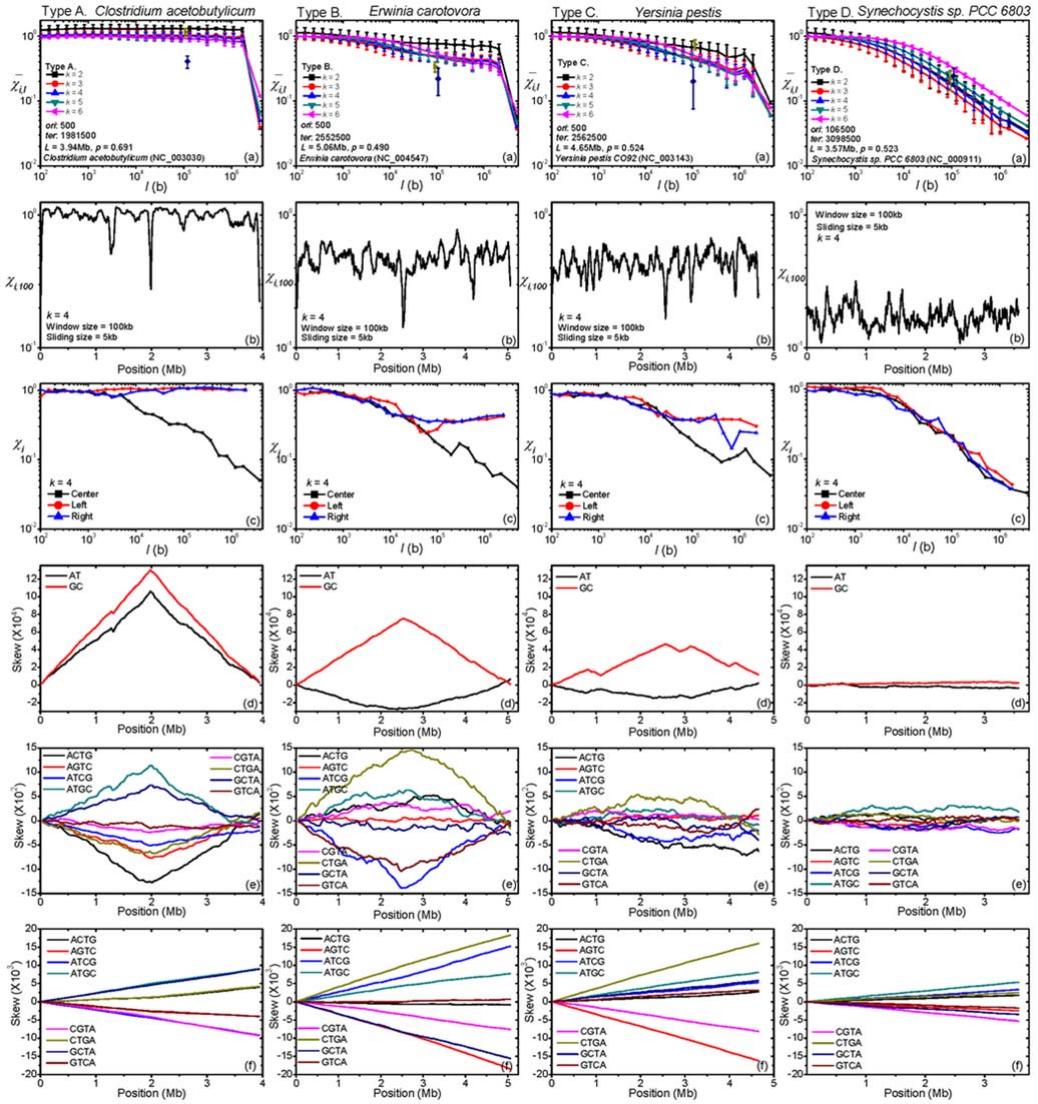
Some 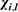 characteristics and cumulative 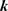-mer-skews in the four types. The four column of panels are, left to right, for *C. acetobutylicum*, type A, *E. carotovora*, type B, *M. mazei*, type C, and *Synechocystis*, type D. In each column: (a) Mean segmental 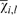 *vs.* segment length 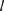; see also caption of Fig. 1 (Methods). (b) Fixed segment-length 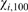 along a chromosome; data obtained by a sliding window of size 100 kb and slide 5 kb. (c) 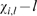: (□) 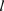 measured with *ter* (or *ori*, whichever is near mid-chromosome) as center; (•) 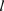 measured from *ter* towards 5′ end; (Δ) 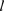 measured from *ter* towards 3′ end. Chromosome is treated as being circular. (d) Cumulative GC- and AT-skews. (e) Cumulative inverse skews in eight base-neutral, inverse-conjugate pairs of 4-mers; e.g., ACTG denotes the ACTG-CAGT pair. (f) Cumulative complement skews in eight base-neutral, complement-conjugate pairs of 4-mers; e.g., ACTG denotes the ACTG-TGAC pair.

Some characteristics of chromosomes that cause each to have a distinct 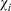-matrix plot are evident in their 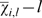 plot. The (a) panels in Fig. 8 illustrate the four type-distinct 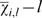-plots. These plots have two prominent features: (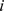) From 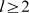 kb onward, except the data near full length, 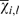 and 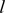 have an approximate power-law relation, with the power-law exponent increasing from type A (in which case it is almost zero) to type D; (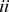) 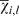 tends to show a discontinuity when 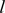 is near full length, with the discontinuity increasing from type D (not apparent) to type A. The magnitude of the continuity is given by 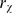 (Methods), with 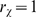 indicating no discontinuity. For a type-A chromosome, the large value of 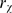 (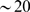) is directly related to four-quadrant structure of its 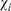-matrix plot (Fig. 5 (a)). For 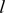 less than half the full sequence length 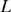, 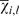 gives the (averaged) values of 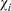 for segments that are entirely either in the lead-strand or in the lag-strand of the chromosome. Since in these halves inverse symmetry is essentially absent, 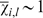. That 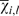 decreases from about 1 at 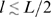 to about 0.05 at 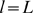 implies the 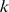-mers in the lead- and lag-strands have a strong inverse-conjugate relation.

We now analyze in detail the plots in Fig. 8 (a) near 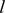 equals to full length 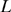 where the discontinuity of data occurs. Let 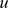 be a typical 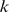-mer (say, AACGC, in the case of 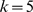) in an 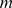-set (for the present example it is the 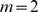 set), and 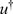 (GCGTT) be its inverse-conjugate. Consider the two halves of the chromosome, the lead-strand and the lag-strand, and let 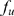 and 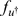 (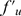 and 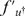, resp.) be the frequencies of 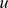 and 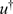 from the lead-strand (lag-strand). Then the data near 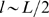 in panels (a) of Fig. 8 imply that on average (Methods)
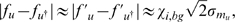
(6)where 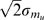 is the average fluctuation of the frequencies of all 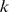-mers in the 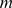-set (the subscript 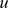 in 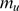 indicates the 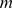-set to which the pair 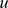 and 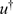 belongs). The value of 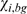 ranges from 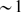 for type A, implying that LIS is absent in both the lead- and lag-strands of the chromosome, to being in the range of 0.2 to 0.5 for types B and C, implying LIS is moderate, to 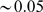 for type D, implying LIS being strongly present in both halves. The data at the end points giving the discontinuity may be written as
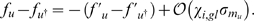
(7)


The last term is expressed as a remainder because, regardless of type, the global 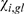 is typically small, of the order of 0.05. Significantly, the negative sign attached to the right-hand-side characterizes a “mutual inverse relation” between the lead- and lag-strands. To summarize, a strong LIS is absent in type A but present in type D everywhere, and the strong GIS in type A is an expression of a lead-lag mutual inverse relation while in type D it may be an extension of LIS.

### *Ori* and *ter* Sites Are Revealed by 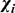-Scanning

Each of the (b) panels in Fig. 8 gives the result of segmental 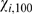 when a chromosome is scanned by a 100 kb wide sliding window. In each case 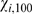 fluctuates around a nearly constant background value that is typical; about 1.0, 0.5, 0.3, and 0.15 for types A, B, C, and D, respectively. Over the background are isolated sharp minima, prominent in types A and B and less conspicuous in type C but absent in type D. As a general rule, in types A and B the two deepest minima occur near the *ori* and *ter* sites. In type C the minima near these sites are two among many. In type D there is no longer any feature that is conspicuously associated with either site. It has been shown that 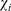-scanning is an effective tool for locating *ori* and *ter* sites for non-type-D chromosomes [25]. Known or putative *ori* and *ter* sites in all prokaryotic chromosomes studied are given in the ISDB [23].

### *Ori* or *ter* Sites Are Centers of Inverse-Symmetry Reflection (CIR)

In the (a) plots of Fig. 8 the ordinates of the 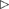 and 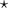 symbols give 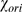 and 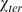, the values of 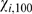 at the *ori* and *ter* sites, respectively. The fact that at least one of 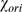 or 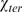 is noticeably less than the average 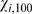 (except for type D) is another indication that segments straddling the *ori* and *ter* sites tend to have a high level of inverse symmetry. To further test this inference, we compute three types of 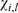-plots, with results shown in the (c) panels of Fig. 8. The square symbols represent data for segments whose centers are either the *ori* or the *ter* site, whichever is nearer the midpoint of the chromosome, the bullet (triangle, resp.) symbols represent data for segments that start from *ori* or the *ter* site and extend towards the 5′ (3′ resp.) end. The results indicate that while the bullet- and triangle-symbol data are type-dependent and tract the 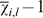 plot of the chromosome ((a) panels), the square-symbol data are similar for every chromosome and drop rapidly with increasing 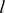 regardless of type. This confirms our interpretation that *ori* and *ter* site are (near) centers of inverse-symmetry reflection (CIR).

### CIRs Are Turning Points for Inverse Skews

Cumulative base-skews, or compositional asymmetry, have been noticed to “turn” at loci near *ori* and *ter* sites [18], [27]–[30]–here called CIRs–and this fact has been used to locate such sites [31]. This phenomenon can be understood as a consequence of the pair of relations Eqs. (6) and (7) when applied to monomers. The relations however predicate a more general phenomena unrelated to the overall base composition of a chromosome: inverse skews in 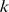-mers (not just in monomers) is strongest in type A and weakest in type D. The panels (d) of Fig. 8 show, in the four representative chromosomes, cumulative GC- and AT-skews, and panels (e) show cumulative inverse skews in eight base-neutral, inverse-conjugate 4-mer-pairs. The correlation between the magnitudes (determined by 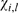) of the skews and type is evident. Eq. (7) states that the slope of a cumulative inverse skew, when it is measurable, must change sign at a CIR. Our relations make no prediction with regards to the relative magnitudes and signs of the GC- and AT-skews. Data on monomer, 2-mer, and 4-mer inverse-skews in all prokaryotic chromosomes studied are give in the ISDB [23].

### Complement and Reverse Skews Are the Norm and Do Not Have Turning Points

From Eq. (6), and because complement and reverse symmetries are both absent (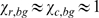), pervasive cumulative complement and reverse skews in 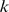-mers *without* turning points are expected in all chromosomes. We have verified this to be true in general. As examples, panels (f) in Fig. 8 show cumulative reverse skews in eight base-neutral, complement-conjugate 4-mer-pairs in the four representative chromosomes (see Figure S3, 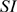 for more examples).

## Discussion

### A Quantitative Description of the Three Prototypes of Cumulative 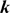-mer-Skews

The relation between base-skew [18], [27]–[33] and complement/inverse symmetry has been noted previously [13]–[16], [34]. Rocha et al. [35] made a comprehensive review of the pro and con of eight hypotheses, including the most often evoked cytosine deamination, genome rearrangements, recombination signals, put forward to explain compositional skews. They concluded that whereas all the (eight) hypotheses have the potential to explain part of available data, none is entirely satisfactory, and that the simplest explanation is that the bias is multifactorial.

Our study shows that cumulative skews in 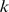-mers, including monomer, have three prototypes: up-and-up (row (f), Fig. 8; Figure S3, 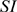), up-and-down (or down-and-up) (rows (d) and (e), type-A and -B columns, Fig. 8), and flat (rows (d) and (e), type-D column, Fig. 8). The classification is dictated by Eqs. (6) and (7) and the values of 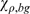 and 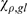: up-and-up if 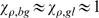; up-and-down if 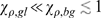; flat if 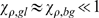. Thus, the cumulative skew between an inverse-conjugate pair of 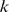-mers (including monomers) will be up-and-down in type-A or -B chromosomes (mildly so for type-C), flat in type-D chromosomes, and never up-and-up in any chromosome. In contrast, the cumulative skew between a reverse-conjugate or complement-conjugate pair of 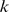-mers, or any pair of 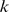-mers that are not inverse-conjugate (unless the pair are related by a hidden, not yet discovered symmetry), will be up-and-up in any chromosome. In other words, up-and-up manifests no symmetry, up-and-down manifests strong global symmetry but weak or no local symmetry, and flat manifests strong local (and consequentially global) symmetry. To sum: cumulative skew of the up-and-up type is the norm, to be expected between a randomly selected pair, and the other two types are special, occurring only between inverse-conjugate pairs.

In the up-and-up and up-and-down types, the approximate constant of the slope of the cumulative skew is a reflection of the typical approximate uniformity of 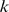-mer-content on a scale greater than 25 kb in most chromosomes [36], [37]. Eq. (6) shows the key to the magnitude of the slope is 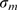. It has been pointed out that 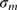 has an approximate 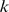
- and 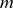-dependent universal (for all genomes) value: 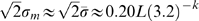 (to within a factor of two) [19] (Strictly, 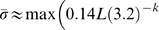, 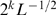), so the maximum 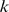 for which the first expression applies is 10 and 14 for 2 Mb and 200 Mb chromosomes, respectively). This means that, typically, the full values (when 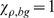) for skews are about 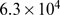 b per Mb for monomers, 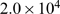 b per Mb for 2-mers, and about 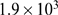 b per Mb for 4-mers. These estimates, together with the computed 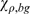 and 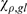, give a reasonable quantitative account of the 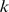-mer-skews we observe, including data shown in Fig. 8 (d–e) and in Figure S3, 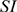. An in-depth quantitative study of this subject will be reported elsewhere.

### Inverse Duplication Generates Inverse Symmetry

**Figure 9:**
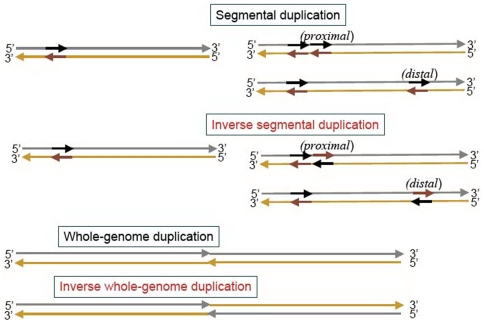
A schematic inverse segmental duplication event. In an inverse segmental duplication, a segment is copied, inverted, and reinserted into the host sequence. In such an event, because the inverse conjugates of all 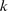-mers appearing in the copied segment is added to the sequence via the inverse duplicate, such a duplication event enhances global inverse symmetry (GIS).

Segmental duplication is known to be a driving force in chromosome growth and evolution [38]–[43], and inverse segmental duplication (ISD), or segmental duplication from one strand of the DNA to the other strand (Fig. 9), is also known to have occurred in chromosome evolution [44]–[46]. “Countless inversions and inverted transporsitions,” which are types of ISD events, were invoked to explain patterns of violation of CPR2 in 3-mers [47], [48]. Instead of being mass produced by ISD, inverse-conjugate pairs may also be generated in a deliberate base-by-base process in the form of stem-loop extrusions (SLEs) from duplex DNA [12], to be discussed at length in a later section. We know of no mechanism analogous to ISD that can stochastically generate either reverse or complement symmetry on a large scale, and this may explain why these two symmetries are not prominent in chromosomes.

The fraction of a sequence that causes it to manifest inverse-inverse symmetry is estimated in mean-field approximation to be 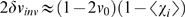 (Eq. (5), Methods), where 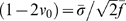 is the fraction of unrelated 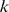-mers that are paired-up and 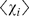 is the mean-field approximation of 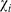. For sequences with strong inverse symmetry (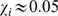), 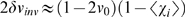. Using the universal value for 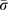 given above and noting that 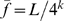 we have 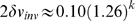 (for 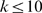), which for 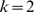 to 6 yield
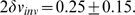
(8)


This suggests that inverse-symmetry generating mechanisms played a major role in chromosome composition.

### Codon Usage and Inverse Symmetry

We analyze possible effects of codon usage on inverse symmetry. The 3-mer and codon frequencies from a DNA sequence differ in two aspects. First, codons are counted in the natural orientations of the genes, while 3-mers are counted along the gene concatenate in one direction (Methods). For example, the codon Trp, or UGG, encoded in a negatively oriented gene (or in a gene in the negative strand) adds one count to the frequency of codon Trp, but one count to that of the 3-mer CCT, not AGG. Second, codons are read only from protein-coding genes and are frame defined, while 3-mers are read over the entire sequence–non-coding as well as coding parts–using a sliding window of slide one. Therefore, the summed frequency of the codons (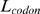) is one-third the summed length of protein-coding genes, and that of 3-mers (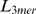) is the length of the DNA sequence (minus 2). In prokaryotes, where the coding region is typically about 88% of the chromosome, the ratio 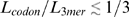, so the likelihood that codon usage could determine the inverse symmetry of the entire chromosome is already small. In increasingly advanced eukaryotes the ratio becomes progressively much less than 1/3–in human it is about 0.01, and the likelihood decreases accordingly. Table 2 uses the coding region (gene concatenate) in the four type-representative chromosomes to illustrates the difference between inverse symmetries in codons and 3-mers. Over the entire gene concatenate 3-mers have excellent symmetry even as codons have no or very poor symmetry. This can be the manifest of “genic inverse symmetry”, meaning that the two sets of positively and negatively oriented genes are broadly homologous. Genic inverse symmetry ensures 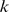-mer inverse symmetry in the coding region, independent of codon usage. The “lead” and “lag” results in Table 2 show that, just as for 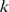-mers, genic inverse symmetry may also be global (type A), or local (type D), or shades in between. Even good genic inverse symmetry is insufficient to explain the 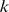-mer inverse symmetry we observe, because the latter is of the entire chromosome, which includes both coding and non-coding regions. Therefore, genic inverse symmetry cannot be the cause genomic inverse symmetry. Rather, it is a consequence of whatever that causes genomes to have inverse symmetry.

**Table 2:** Inverse symmetry indexes for codons and 3-mers.

Type	Genes	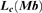	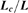	*ori*	*ter*	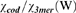	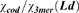	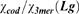
A	3882	3.42	0.87	500	1981500	1.112/0.032	1.112/1.043	1.112/1.040
B	4501	4.35	0.89	500	2552500	0.657/0.055	0.650/0.412	0.665/0.438
C	4205	3.90	0.84	500	2562500	0.735/0.055	0.734/0.306	0.737/0.307
D	3462	3.13	0.88	106500	3098500	1.019/0.028	0.813/0.037	1.021/0.074

Inverse symmetry indexes computed from counting codons in genes (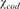) and from counting 3-mers in gene-concatenates (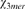) for four type-representative chromosomes. The four typical bacterial genomes are: A, *C. acetobutylicum*; B, *E. carotovora*; C, *Y. pestis*: and D, *Synechocystis*. Given a segment - Whole, Lead or Lag - the number of codons is counted in all gene-embedding sequences in the segment, and the number of 3-mers is counted in the gene-concatenate formed by stitching together all gene-embedding sequences (Methods). In the last three columns listing 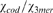, “W” means gene-embedding sequences are spliced from the entire chromosome, “Ld”, from the ori to ter strand, and “Lg”, from the ter to ori strand. Codons do not exhibit inverse symmetry ((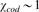)) in all cases. 3-mers exhibit strong inverse symmetry ((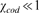)) in “W” always, and in “Ld” and “Lg” according to type.

### Type A Suggests Chromosome-Size Inverse Duplication and the *prox* Hypothesis

**Figure 10:**
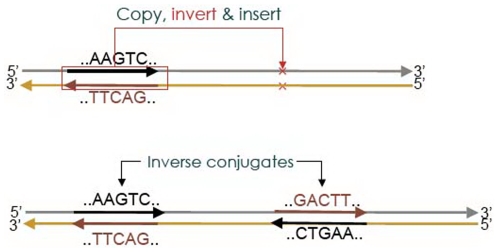
Modes of segmental duplication. Proximal and distal modes of segmental duplications (top) and direct and inverse whole-genome duplications (bottom).

**Figure 11:**
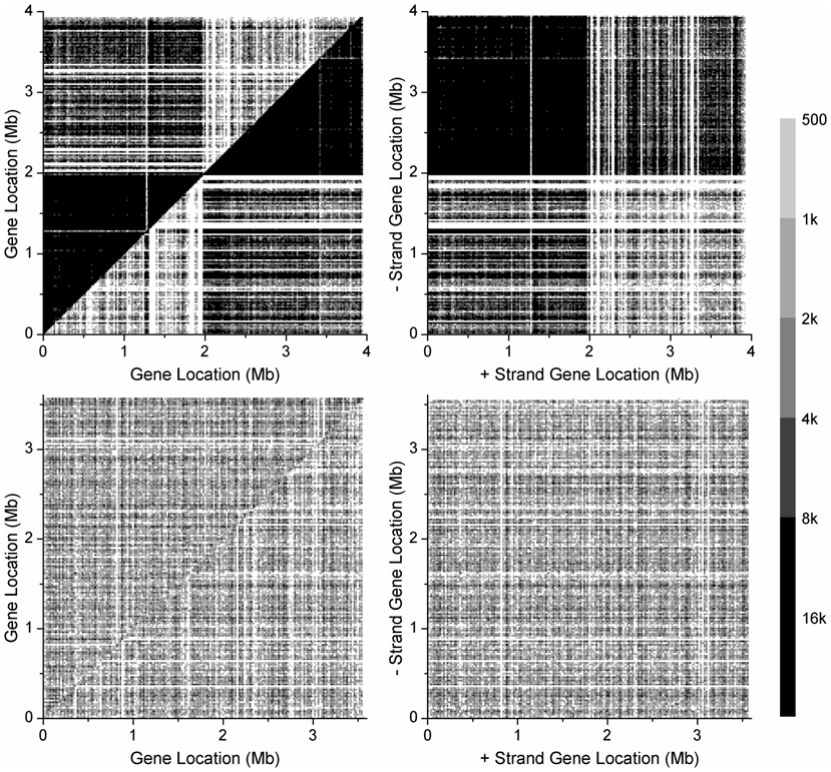
BLAST plots of homologs in *C. acetobutylicum* and *Synechocystis*. The top pair of plots are for *C. acetobutylicum* and the bottom plots pair are for *Synechocystis*. In each plot, coordinates are sites of homologs on the chromosome. Pixel size is 20 kb by 20 kb and in each pixel BLAST scores for pairs of homologs falling in the pixel are summed. Cut-off scores of 500 for *C. acetobutylicum* and 400 for *Synechocystis* are imposed to remove random background. Scores are given in log2 gray scale. Plots on left: top-left (bottom-right) triangle gives BLAST scores for intra-strand homologs on the positive (negative) strand; pixels on the diagonals, which include very high scores from same-gene BLASTs, are removed. Plots on right: BLAST scores for inter-strand-homologs; x-axis (y-axis) gives sites on the positive (negative) strand. The bottom plots suggest a relatively low level of homology in the type-D *Synechocystis* for both inter-strand and inter-strand pairs. To discuss the feature-rich top plots for the type-A *C. acetobutylicum* we use the obvious notation to denote the four half-strands of the double-stranded chromosome: (+5), (+3), (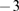), and (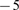). The intra-strand (top-left) plot suggests that in *C. acetobutylicum* the (+5) and (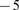) half-strands are rich in homologs and the (+3) and (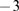) half-strands are (relatively) poor in homologs. The dark top-left quadrant in the inter-strand (top-right) plot suggest that (+5) and (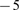) share many pairs of genes with high degrees. The pattern of the BLAST plots for *C. acetobutylicum* is consistent with the hypothesis that its chromosome experienced a WGID, and that before that event the positive strand was gene and homolog rich while the negative strand was either gene or homolog poor, or both. Furthermore, after the WGID event few 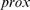 ISD event occurred.

To simplify discussion we define the following: a *prox* duplication is one such that the site of insertion of the duplicated segment is proximal (relative to chromosome-scale) to the site of duplication; a *dist* duplication, necessarily trans-CIR, is one such that the site of insertion is distal to the site of the duplication (Fig. 10). A *prox*-ISD tends to enhance LIS–near the location of the ISD event–as well as GIS, whereas a *dist*-ISD can enhance only GIS. The most parsimonious explanation for the fact that a type-A chromosome has strong GIS while both of its two approximately equal sized halves–the lead and lag strands–are without inverse symmetry is that it is the result of a whole-genome/chromosome ISD (WGID) on a chromosome that had no inverse symmetry before the event. Following a WGID event, an originally symmetry-free chromosome will have perfect GIS, with 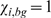 but 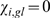, which defines an extreme type-A chromosome. The 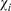-matrix plot of a chromosome immediately after an WGID event will not look exactly like Fig. 5 (a), however. Its skew-diagonal quadrants will be mostly white, just like in Fig. 5 (a). The two dark diagonal quadrants seen in panel (a) will be replaced by quadrants of a much lighter shade (but not white, owing to the fact that the word-content of a chromosome has a fair degree of homogeneity), with a black, narrow diagonal strip running through it. The actual type-A patterns seen in Figs. 4 and 5 could be the consequence of (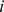) very few of either *prox*-ISD or *dist*-DSDs (direct segmental duplications) but (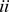) many *prox*-DSDs occurring after the WGID. If we assume that a WGID is the major event giving rise to the pattern of inverse symmetry in type-A chromosomes, then (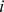) and (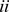) above may be viewed as constraints that need to be satisfied. Since it is known that DSD is a major driving force in genome growth [38]–[43], satisfaction of constraint (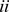) and the second half of constraint (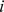) follows if we hypothesize that as a general rule SDs are mostly *prox*. We call this the “ *prox* hypothesis”. (This hypothesis is consistent with an unrelated requirement, put forward for understanding the general existence of long-range correlation in genomes, that at least a significant portion of DSDs are made in tandem [20].) The *prox* hypothesis drastically simplifies the narrative for a type-A chromosome: a chromosome suffers a WGID, after which very few *prox*-ISD occurred. If a WGID event did occur then we should find homologs between the two arms of the chromosome. Our preliminary study using sequence alignment indicates that the pattern of homologs is consistent with the hypothesis that a WGID occurred in a type-A chromosome (*C. acetobutylicum*) and not in a type-D chromosome (*Synechocystis*), as shown in Fig. 11. On the other hand, both chromosomes exhibit good genic inverse symmetry in ways that are expected, global in type A and local in type D. A comprehensive BLAST-based study of this topic is underway and results will be reported elsewhere. An alternative explanation for type A is that there had been no WGID, instead the high degree of inverse symmetry was the result of many *dist*-ISDs, such that the cumulative length of inversely copied segments was close to (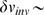) 13% of the full chromosome length (Eq. (8)). For this explanation to work constraint (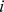) is still needed. This alternative explanation seems highly unnatural since it implies there were simultaneously many *dist*-ISDs and very few *prox*-ISDs.

Whole-genome duplication (WGD) was first proposed by Ohno as an important mechanism for genome evolution [49]. Recently it has been firmly established that such events did occur in yeast [50]–[52], ray-finned fishes [53], and freshwater puffer fish [54]. The possibility of WGID was previously discussed in connection with base-skews in *B. burgdorferi* [16], [34].

### Type D Suggests Many *prox*-ISD Events

In a type-D chromosome, the existence of inverse symmetry on all scales (greater than 5 kb) including local genic inverse symmetry (Fig. 11) and the homogeneity of 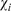 across the entire chromosome (type-D patterns in in Figs. 4, 5, and 6) can best be explained as the result of many small, and mostly *prox*, ISD events; necessarily *prox* because otherwise LIS on a small scale would not be generated. This explanation is consistent with the *prox* hypothesis. Our results suggest that the upper bound of the distance between copying and insertion sites in a *prox*-ISD should be considerably less than 100 kb. In spite of the absence of distinct CIRs in type-D chromosomes (type D in Fig. 8 (b,c)), we may not rule out the possibility that early WGIDs did occur in such chromosomes, because much of the trace of an early WGID, assuming that it had occurred, would have been obliterated by the large number of small ISDs that came afterwards.

### Possible Role of SLE in Local Inverse Symmetry

Extrusion of a stem-loop (SLE) from duplex DNA [12] can enhance local inverse symmetry in general and CPR2 in particular. If such structures are of adaptive significance, then in a scenario of “Nature (writing) with parity primarily at the oligonucleotide level”, organisms which had single base mutations that strengthened the stem would have been selected over organisms that had not [24]. Inverse-conjugate pairs formed in such a process are necessarily extremely proximal to each other, with a separation not exceeding tens of base pairs. SLE cannot be the sole cause of inverse symmetry because unlike *prox*-ISD it does not generate local genic inverse symmetry, which is prevalent in type-D chromosomes (Table 2 and Fig. 11). Furthermore, SLE promotes inverse symmetry at the expense of violating Chargaff's (first) parity rule [7] (CPR1), a key to DNA replication and biological inheritance. The CPR1-violating effect of SLE may be lessened if it is considered as a mechanism that generates, as opposed to a structural form that manifests, inverse symmetry. In this case SLE exists as a extruding structure only during its formative phase (which cannot be too short a time if it is to be formed one nucleotide at a time [12]), but subsequently becomes a part of the (non-extruding) stem-loop structure [55] via DNA replication (making two copies that are slightly different). Then only a small number of structural SLEs need to exist in the chromosome at any given time, causing only a miniscule violation of CPR1. Current sequencing techniques based on the shotgun method cannot distinguish SLS from SLE. The number of SLSs in excess of expected background with minimum stem length of 12 bp and loop length ranging from 5 to 100 nt was measured to be 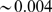 per base in 40 prokaryotes [55]. If we assume all SLSs with loop lengths less than 50 nt–about half the total [55]–are generated by the SLE mechanism then we estimate the SLE contribution to 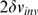 to be about 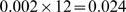, about one-tenth of the full value of 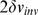 (Eq. (8)).

### A Unified Interpretation for All Types

If we view types B and C as intermediates between A and D, then a unified interpretation of the behavior of 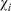 for all types emerges: Every chromosome, with the possible exception of type-Ds, experienced a WGID. The chromosomes differ mainly in the amount of *prox*-ISDs each had, in increasing amount from type A to D, with type A hardly any, and type D close to the saturation amount, involving a fraction of 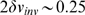 of the chromosome. In all cases a large number (unconstrained as far as inverse symmetry is concerned) of *prox*-DSD events may have happened, while *dist* events, either ISDs or DSDs, occurred rarely. A fraction of highly proximal inverse-conjugate pairs, possibly contributing to up to one-tenth of the local inverse symmetry in type D chromosomes, and possibly a larger fraction in the background component in the inverse symmetry in the other types, may have been generated by SLE instead of ISD. The DSD and ISD (and SLE) events that occurred were mostly neutral, because the coding and non-coding parts in a chromosome do not differ significantly in their patterns of inverse symmetry (Figure S2, 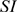). On the other hand, because segments involved in ISD (as well as DSD) events sometimes contained genes, ISD enhanced genic inverse symmetry just as it did 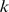-mer inverse symmetry, so the unified interpretation explains the results seen in Table 2 and in Fig. 11. Many alternative interpretations are possible for our data, but none will be as unifying, simple, and parsimonious as the one proposed here. We believe that by refining and expanding the analysis reported here, a great deal more about how genomes grew and evolved can be learned.

Our study suggests that the ISD events, if they did occur, were causatively related to DNA replication. First, we found it consistent to identify the sites of insertion of WGID events in type-A, B and C chromosomes (the CIRs) with *ori* or *ter* sites. Second, genomes known to have multiple *ori* sites tend to be archaeons and eukaryotes [56], [57], not eubacteria, and we found archaeons and eukaryotes tended to be type D and never type A, while eubacteria tended to be the opposite. On the other hand, some type-D chromosomes are from eubacteria, and half of archaea are not type D. It could be that not all ISD events are associated with replication, or that some eubacteria also have multiple *ori* sites while some archeaons do not, or both. We offer no explanation why replication may cause ISD, except to point out that during replication the chromosome is spliced at the *ori* site, and this offers opportunities for the chromosome to misconnect on rare occasions, possibly resulting in an ISD event. In any case, the genome seemed to have developed machineries for ISD and used it frequently, probably because ISDs allow it to efficiently exploit its double-stranded structure to enrich its code-inventory.

## Supporting Information

Table S1 List of chromosomes by taxonomy and global *k*-averaged symmetry index of reverse, complement and inverse symmetries.
(1.46 MB DOC)

Table S2 Classification of chromosomes by inverse symmetry type and *χ_i,bg_* and *r_χ_* values.
(0.83 MB DOC)

Table S3 List of 38 exceptional chromosomes and their *χ_i_* values.
(0.09 MB DOC)

Figure S1 *χ_i,l_-l* plots for six eukaryotes (number of chromosomes in parentheses). (a) Yeast (16), (b) Worm (6), (c) Fly (6), (d) Human (24), (e) *P. falciparum*(14), (f) *E. cuniculi* (11). In each case the result for all chromosomes are overlayed. Results for other *k*-mers are similar.
(1.26 MB TIF)

Figure S2 *χ_i,l_*-*l* plots for the coding and non-coding parts of (a) the type-A eubacterial *B. burgdorferi* (5% of chromosome is non-coding), (b) the type-D archaeon *M. acetivorans* (29%), (c) the type-C chromosome 14 of the protozoan *P. falciparum* (41%), and (d) the type-D chromosome 1 of human (49%).
(0.92 MB TIF)

Figure S3 Cumulative skews of 12 base-neutral reverse- and complement-conjugate paris of 4-mers in four types of chromosomes. Reverse (a) and complement (b) skews in type-A *C. acetobutylicum*; reverse (c) and complement (d) skews in type-B *E. carotovora*; reverse (e) and complement (f) skews in type-C *M. mazei*; reverse (g) and complement (h) skews in type-D *Synechocystis*.
(1.29 MB TIF)
