# Supplementary material for: Inverse Symmetry in Complete Genomes and Whole-Genome Inverse Duplication
Source: PLoS One. 2009 Nov 9;4(11):e7553. doi: 10.1371/journal.pone.0007553 (PMC2771390; doi:10.1371/journal.pone.0007553)
Supplement: Table S1 — List of chromosomes by taxonomy and global k-averaged symmetry index of reverse, complement and inverse symmetries. (1.46 MB DOC) [file pone.0007553.s001.doc]

Table S1. List of chromosomes by taxonomy and global *k*-averaged symmetry index of reverse, complement and inverse symmetries

| **28 Archaeal chromosomes** | **Accession no.** | | ***χ_r_*,*_gl_*** | | ***χ_c_*,*_gl_*** | | ***χ_i_*,*_gl_*** | | ***L* (Mb)** | | ***p*** | **Type** |  |
| --- | --- | --- | --- | --- | --- | --- | --- | --- | --- | --- | --- | --- | --- |
| *A. pernix K1* | NC_000854 | | 0.871 | | 0.814 | | 0.121 | | 1.670 | | 0.437 | D |  |
| *A. fulgidus DSM 4304* | NC_000917 | | 0.777 | | 0.721 | | 0.055 | | 2.180 | | 0.514 | D |  |
| *H. marismortui ATCC 43049* | NC_006396 | | 1.174 | | 1.086 | | 0.041 | | 3.130 | | 0.376 | D |  |
| *H. marismortui ATCC 43049* | NC_006397 | | 1.171 | | 1.090 | | 0.198 | | 0.290 | | 0.428 | C |  |
| *H. salinarum NRC-1* | NC_002607 | | 1.165 | | 1.077 | | 0.062 | | 2.010 | | 0.321 | C |  |
| *M. jannaschii DSM 2661* | NC_000909 | | 0.886 | | 0.824 | | 0.091 | | 1.670 | | 0.686 | D |  |
| *M. burtonii DSM 6242* | NC_007955 | | 1.214 | | 1.128 | | 0.109 | | 2.580 | | 0.592 | C |  |
| *M. maripaludis S2* | NC_005791 | | 1.023 | | 0.950 | | 0.092 | | 1.660 | | 0.669 | C |  |
| *M. kandleri AV19* | NC_003551 | | 1.183 | | 1.094 | | 0.086 | | 1.700 | | 0.388 | D |  |
| *M. acetivorans C2A* | NC_003552 | | 0.849 | | 0.791 | | 0.056 | | 5.750 | | 0.573 | D |  |
| *M. barkeri str. fusaro* | NC_007355 | | 0.907 | | 0.845 | | 0.061 | | 4.840 | | 0.607 | D |  |
| *M. mazei Go1* | NC_003901 | | 0.887 | | 0.825 | | 0.041 | | 4.100 | | 0.585 | C |  |
| *M. stadtmanae DSM 3091* | NC_007681 | | 1.013 | | 0.946 | | 0.164 | | 1.770 | | 0.724 | C |  |
| *M. hungatei JF-1* | NC_007796 | | 1.192 | | 1.109 | | 0.089 | | 3.550 | | 0.549 | D |  |
| *M. thermautotrophicus str. Delta H* | NC_000916 | | 1.043 | | 0.967 | | 0.074 | | 1.750 | | 0.505 | D |  |
| *N. equitans Kin4-M* | NC_005213 | | 0.813 | | 0.755 | | 0.075 | | 0.490 | | 0.684 | C |  |
| *N. pharaonis DSM 2160* | NC_007426 | | 1.106 | | 1.023 | | 0.048 | | 2.600 | | 0.366 | D |  |
| *P. torridus DSM 9790* | NC_005877 | | 1.232 | | 1.141 | | 0.080 | | 1.550 | | 0.640 | D |  |
| *P. aerophilum str. IM2* | NC_003364 | | 0.958 | | 0.896 | | 0.150 | | 2.220 | | 0.486 | D |  |
| *P. abyssi GE5* | NC_000868 | | 0.497 | | 0.467 | | 0.057 | | 1.770 | | 0.553 | C |  |
| *P. furiosus DSM 3638* | NC_003413 | | 0.654 | | 0.610 | | 0.042 | | 1.910 | | 0.592 | C |  |
| *P. horikoshii OT3* | NC_000961 | | 0.511 | | 0.484 | | 0.086 | | 1.740 | | 0.581 | C |  |
| *S. acidocaldarius DSM 639* | NC_007181 | | 0.660 | | 0.615 | | 0.090 | | 2.230 | | 0.633 | C |  |
| *S. solfataricus P2* | NC_002754 | | 0.604 | | 0.582 | | 0.165 | | 2.990 | | 0.642 | C |  |
| *S. tokodaii str. 7* | NC_003106 | | 0.720 | | 0.675 | | 0.108 | | 2.700 | | 0.672 | D |  |
| *T. kodakarensis KOD1* | NC_006624 | | 0.511 | | 0.483 | | 0.050 | | 2.090 | | 0.480 | D |  |
| *T. acidophilum DSM 1728* | NC_002578 | | 1.166 | | 1.082 | | 0.083 | | 1.570 | | 0.540 | C |  |
| *T. volcanium GSS1* | NC_002689 | | 0.986 | | 0.914 | | 0.094 | | 1.590 | | 0.601 | C |  |
| *A. pernix K1* | NC_000854 | | 0.871 | | 0.814 | | 0.121 | | 1.670 | | 0.437 | D |  |
| **356 Eubacterial chromosomes** | **Accession no.** | | | ***χ_r_*,*_gl_*** | ***χ_c_*,*_gl_*** | | ***χ_i_*,*_gl_*** | | ***L* (Mb)** | | ***p*** | **Type** | |
| *A. bacterium Ellin345* | NC_008009 | | | 1.019 | 0.944 | | 0.028 | | 5.650 | | 0.416 | D | |
| *Acinetobacter sp. ADP1* | NC_005966 | | | 1.165 | 1.080 | | 0.055 | | 3.600 | | 0.596 | B | |
| *A. tumefaciens str. C58* | NC_003062 | | | 1.094 | 1.016 | | 0.091 | | 2.840 | | 0.406 | D | |
| *A. tumefaciens str. C58* | NC_003304 | | | 1.094 | 1.016 | | 0.091 | | 2.840 | | 0.406 | D | |
| *A. tumefaciens str. C58* | NC_003063 | | | 1.124 | 1.042 | | 0.042 | | 2.070 | | 0.407 | C | |
| *A. tumefaciens str. C58* | NC_003305 | | | 1.124 | 1.042 | | 0.042 | | 2.080 | | 0.407 | C | |
| *A. variabilis ATCC 29413* | NC_007413 | | | 1.058 | 0.980 | | 0.067 | | 6.370 | | 0.586 | D | |
| *A. dehalogenans 2CP-C* | NC_007760 | | | 0.865 | 0.798 | | 0.085 | | 5.010 | | 0.251 | D | |
| *A. marginale str. St. Maries* | NC_004842 | | | 1.152 | 1.071 | | 0.139 | | 1.200 | | 0.502 | A | |
| *A. phagocytophilum HZ* | NC_007797 | | | 1.163 | 1.124 | | 0.328 | | 1.470 | | 0.584 | A | |
| *A. aeolicus VF5* | NC_000918 | | | 0.454 | 0.427 | | 0.061 | | 1.550 | | 0.565 | D | |
| *A. yellows* | NC_007716 | | | 0.857 | 0.798 | | 0.163 | | 0.710 | | 0.731 | C | |
| *Azoarcus sp. EbN1* | NC_006513 | | | 1.047 | 0.969 | | 0.033 | | 4.300 | | 0.349 | C | |
| *B. anthracis str. Ames* | NC_003997 | | | 0.939 | 0.871 | | 0.099 | | 5.230 | | 0.646 | A | |
| *B. anthracis str. 'Ames Ancestor'* | NC_007530 | | | 0.939 | 0.871 | | 0.099 | | 5.230 | | 0.646 | A | |
| *B. anthracis str. Sterne* | NC_005945 | | | 0.939 | 0.871 | | 0.101 | | 5.230 | | 0.646 | A | |
| *B. cereus ATCC 10987* | NC_003909 | | | 0.940 | 0.869 | | 0.064 | | 5.220 | | 0.644 | A | |
| *B. cereus ATCC 14579* | NC_004722 | | | 0.962 | 0.888 | | 0.053 | | 5.410 | | 0.647 | A | |
| *B. cereus E33L* | NC_006274 | | | 0.942 | 0.872 | | 0.085 | | 5.300 | | 0.646 | A | |
| *B. clausii KSM-K16* | NC_006582 | | | 0.946 | 0.874 | | 0.048 | | 4.300 | | 0.552 | A | |
| *B. halodurans C-125* | NC_002570 | | | 0.898 | 0.842 | | 0.151 | | 4.200 | | 0.563 | A | |
| *B. licheniformis ATCC 14580* | NC_006270 | | | 0.937 | 0.869 | | 0.031 | | 4.220 | | 0.538 | A | |
| *B. licheniformis ATCC 14580* | NC_006322 | | | 0.937 | 0.869 | | 0.032 | | 4.220 | | 0.538 | A | |
| *B. subtilis* | NC_000964 | | | 1.095 | 1.015 | | 0.045 | | 4.210 | | 0.565 | A | |
| *B. thuringiensis* | NC_005957 | | | 0.938 | 0.869 | | 0.090 | | 5.240 | | 0.646 | A | |
| *B. fragilis NCTC 9343* | NC_003228 | | | 1.060 | 0.980 | | 0.059 | | 5.210 | | 0.568 | A | |
| *B. fragilis YCH46* | NC_006347 | | | 1.058 | 0.979 | | 0.051 | | 5.280 | | 0.567 | A | |
| *B. thetaiotaomicron VPI-5482* | NC_004663 | | | 1.064 | 0.989 | | 0.103 | | 6.260 | | 0.572 | A | |
| *B. henselae str. Houston-1* | NC_005956 | | | 1.146 | 1.073 | | 0.211 | | 1.930 | | 0.618 | A | |
| *B. quintana str. Toulouse* | NC_005955 | | | 1.159 | 1.078 | | 0.153 | | 1.580 | | 0.612 | A | |
| *B. cicadellinicola str. Hc* | NC_007984 | | | 1.060 | 0.982 | | 0.117 | | 0.690 | | 0.668 | C | |
| *B. bacteriovorus HD100* | NC_005363 | | | 1.065 | 0.984 | | 0.045 | | 3.780 | | 0.494 | A | |
| *B. longum NCC2705* | NC_004307 | | | 1.150 | 1.065 | | 0.052 | | 2.260 | | 0.399 | C | |
| *B. bronchiseptica RB50* | NC_002927 | | | 1.124 | 1.041 | | 0.053 | | 5.340 | | 0.319 | C | |
| *B. parapertussis 12822* | NC_002928 | | | 1.125 | 1.043 | | 0.078 | | 4.770 | | 0.319 | C | |
| *B. pertussis Tohama I* | NC_002929 | | | 1.124 | 1.041 | | 0.046 | | 4.090 | | 0.323 | D | |
| *B. burgdorferi B31* | NC_001318 | | | 1.073 | 0.997 | | 0.086 | | 0.910 | | 0.714 | A | |
| *B. garinii Pbi* | NC_006156 | | | 1.047 | 0.974 | | 0.096 | | 0.900 | | 0.717 | A | |
| *B. japonicum USDA 110* | NC_004463 | | | 1.117 | 1.036 | | 0.019 | | 9.110 | | 0.359 | C | |
| *B. abortus biovar 1* | NC_006932 | | | 1.096 | 1.016 | | 0.056 | | 2.120 | | 0.428 | C | |
| *B. abortus biovar 1* | NC_006933 | | | 1.129 | 1.047 | | 0.076 | | 1.160 | | 0.427 | C | |
| *B. melitensis 16M* | NC_003317 | | | 1.096 | 1.015 | | 0.056 | | 2.120 | | 0.428 | C | |
| *B. melitensis 16M* | NC_003318 | | | 1.131 | 1.048 | | 0.054 | | 1.180 | | 0.427 | C | |
| *B. melitensis* | NC_007618 | | | 1.096 | 1.016 | | 0.056 | | 2.120 | | 0.428 | C | |
| *B. melitensis* | NC_007624 | | | 1.129 | 1.047 | | 0.076 | | 1.160 | | 0.427 | C | |
| *B. suis 1330* | NC_004310 | | | 1.096 | 1.015 | | 0.055 | | 2.110 | | 0.428 | C | |
| *B. suis 1330* | NC_004311 | | | 1.130 | 1.047 | | 0.053 | | 1.210 | | 0.427 | C | |
| *B. aphidicola str. APS* | NC_002528 | | | 1.078 | 1.003 | | 0.202 | | 0.640 | | 0.737 | B | |
| *B. aphidicola str. Bp* | NC_004545 | | | 1.028 | 0.964 | | 0.225 | | 0.620 | | 0.747 | A | |
| *B. aphidicola str. Sg* | NC_004061 | | | 1.002 | 0.932 | | 0.222 | | 0.640 | | 0.747 | B | |
| *B. cenocepacia AU 1054* | NC_008060 | | | 1.082 | 1.000 | | 0.042 | | 3.290 | | 0.331 | C | |
| *B. cenocepacia AU 1054* | NC_008061 | | | 1.116 | 1.033 | | 0.046 | | 2.790 | | 0.331 | C | |
| *B. cenocepacia AU 1054* | NC_008062 | | | 1.104 | 1.022 | | 0.081 | | 1.200 | | 0.330 | D | |
| *B. mallei ATCC 23344* | NC_006348 | | | 1.062 | 0.984 | | 0.072 | | 3.510 | | 0.318 | D | |
| *B. mallei ATCC 23344* | NC_006349 | | | 1.095 | 1.014 | | 0.046 | | 2.330 | | 0.310 | C | |
| *B. pseudomallei 1710b* | NC_007434 | | | 1.072 | 0.992 | | 0.043 | | 4.130 | | 0.324 | C | |
| *B. pseudomallei 1710b* | NC_007435 | | | 1.106 | 1.024 | | 0.050 | | 3.180 | | 0.315 | C | |
| *B. pseudomallei K96243* | NC_006350 | | | 1.070 | 0.990 | | 0.057 | | 4.070 | | 0.323 | D | |
| *B. pseudomallei K96243* | NC_006351 | | | 1.105 | 1.023 | | 0.047 | | 3.170 | | 0.315 | C | |
| *Burkholderia sp. 383* | NC_007510 | | | 1.077 | 0.996 | | 0.045 | | 3.690 | | 0.338 | C | |
| *Burkholderia sp. 383* | NC_007511 | | | 1.110 | 1.027 | | 0.033 | | 3.590 | | 0.333 | C | |
| *Burkholderia sp. 383* | NC_007509 | | | 1.132 | 1.048 | | 0.044 | | 1.400 | | 0.347 | C | |
| *B. thailandensis E264* | NC_007651 | | | 1.067 | 0.989 | | 0.079 | | 3.810 | | 0.327 | D | |
| *B. thailandensis E264* | NC_007650 | | | 1.105 | 1.023 | | 0.043 | | 2.910 | | 0.319 | C | |
| *B. xenovorans LB400* | NC_007951 | | | 1.061 | 0.982 | | 0.038 | | 4.900 | | 0.372 | C | |
| *B. xenovorans LB400* | NC_007952 | | | 1.085 | 1.005 | | 0.033 | | 3.360 | | 0.372 | C | |
| *B. xenovorans LB400* | NC_007953 | | | 1.117 | 1.035 | | 0.064 | | 1.470 | | 0.383 | C | |
| *C. jejuni RM1221* | NC_003912 | | | 1.135 | 1.052 | | 0.058 | | 1.780 | | 0.697 | A | |
| *C. jejuni* | NC_002163 | | | 1.137 | 1.054 | | 0.066 | | 1.640 | | 0.695 | A | |
| *C. Blochmannia floridanus* | NC_005061 | | | 1.097 | 1.058 | | 0.353 | | 0.710 | | 0.726 | A | |
| *C. Blochmannia pennsylvanicus* | NC_007292 | | | 1.144 | 1.084 | | 0.295 | | 0.790 | | 0.704 | A | |
| *C. Pelagibacter* | NC_007205 | | | 1.097 | 1.017 | | 0.085 | | 1.310 | | 0.703 | C | |
| *C. Protochlamydia* | NC_005861 | | | 1.068 | 0.990 | | 0.072 | | 2.410 | | 0.653 | B | |
| *C. hydrogenoformans Z-2901* | NC_007503 | | | 0.681 | 0.633 | | 0.041 | | 2.400 | | 0.580 | A | |
| *C. crescentus CB15* | NC_002696 | | | 1.041 | 0.966 | | 0.046 | | 4.020 | | 0.328 | D | |
| *C. muridarum Nigg* | NC_002620 | | | 0.798 | 0.743 | | 0.083 | | 1.070 | | 0.597 | A | |
| *C. trachomatis A/HAR-13* | NC_007429 | | | 0.805 | 0.748 | | 0.084 | | 1.040 | | 0.587 | A | |
| *C. trachomatis D/UW-3/CX* | NC_000117 | | | 0.807 | 0.750 | | 0.083 | | 1.040 | | 0.587 | A | |
| *C. abortus S26/3* | NC_004552 | | | 0.856 | 0.798 | | 0.109 | | 1.140 | | 0.601 | A | |
| *C. caviae GPIC* | NC_003361 | | | 0.862 | 0.802 | | 0.080 | | 1.170 | | 0.608 | A | |
| *C. felis Fe/C-56* | NC_007899 | | | 0.805 | 0.750 | | 0.087 | | 1.170 | | 0.606 | A | |
| *C. pneumoniae AR39* | NC_002179 | | | 0.754 | 0.702 | | 0.094 | | 1.230 | | 0.594 | A | |
| *C. pneumoniae CWL029* | NC_000922 | | | 0.754 | 0.702 | | 0.094 | | 1.230 | | 0.594 | B | |
| *C. pneumoniae J138* | NC_002491 | | | 0.754 | 0.703 | | 0.095 | | 1.230 | | 0.594 | B | |
| *C. pneumoniae TW-183* | NC_005043 | | | 0.754 | 0.702 | | 0.095 | | 1.230 | | 0.594 | B | |
| *C. chlorochromatii CaD3* | NC_007514 | | | 0.923 | 0.856 | | 0.070 | | 2.570 | | 0.557 | C | |
| *C. tepidum TLS* | NC_002932 | | | 0.993 | 0.922 | | 0.078 | | 2.150 | | 0.435 | C | |
| *C. violaceum ATCC 12472* | NC_005085 | | | 1.116 | 1.032 | | 0.045 | | 4.750 | | 0.352 | B | |
| *C. salexigens DSM 3043* | NC_007963 | | | 1.174 | 1.089 | | 0.068 | | 3.700 | | 0.361 | C | |
| *C. acetobutylicum ATCC 824* | NC_003030 | | | 1.010 | 0.937 | | 0.062 | | 3.940 | | 0.691 | A | |
| *C. perfringens str. 13* | NC_003366 | | | 0.874 | 0.850 | | 0.267 | | 3.030 | | 0.714 | A | |
| *C. tetani E88* | NC_004557 | | | 0.852 | 0.805 | | 0.158 | | 2.800 | | 0.713 | A | |
| *C. psychrerythraea 34H* | NC_003910 | | | 0.988 | 0.916 | | 0.042 | | 5.370 | | 0.620 | B | |
| *C. diphtheriae NCTC 13129* | NC_002935 | | | 0.959 | 0.887 | | 0.059 | | 2.490 | | 0.465 | A | |
| *C. efficiens YS-314* | NC_004369 | | | 1.175 | 1.088 | | 0.056 | | 3.150 | | 0.369 | D | |
| *C. glutamicum ATCC 13032* | NC_003450 | | | 1.083 | 1.003 | | 0.073 | | 3.310 | | 0.462 | C | |
| *C. glutamicum ATCC 13032* | NC_006958 | | | 1.082 | 1.002 | | 0.064 | | 3.280 | | 0.462 | C | |
| *C. jeikeium K411* | NC_007164 | | | 0.994 | 0.920 | | 0.145 | | 2.460 | | 0.386 | A | |
| *C. burnetii RSA 493* | NC_002971 | | | 0.802 | 0.740 | | 0.060 | | 2.000 | | 0.573 | A | |
| *D. aromatica RCB* | NC_007298 | | | 1.098 | 1.017 | | 0.045 | | 4.500 | | 0.408 | B | |
| *D. ethenogenes 195* | NC_002936 | | | 0.993 | 0.922 | | 0.098 | | 1.470 | | 0.511 | C | |
| *Dehalococcoides sp. CBDB1* | NC_007356 | | | 0.968 | 0.896 | | 0.063 | | 1.400 | | 0.530 | B | |
| *D. geothermalis DSM 11300* | NC_008025 | | | 1.114 | 1.030 | | 0.073 | | 2.470 | | 0.334 | C | |
| *D. radiodurans R1* | NC_001263 | | | 0.894 | 0.826 | | 0.049 | | 2.650 | | 0.330 | D | |
| *D. radiodurans R1* | NC_001264 | | | 0.910 | 0.844 | | 0.246 | | 0.410 | | 0.333 | C | |
| *D. hafniense Y51* | NC_007907 | | | 1.053 | 1.009 | | 0.307 | | 5.730 | | 0.526 | B | |
| *D. psychrophila LSv54* | NC_006138 | | | 1.068 | 0.995 | | 0.132 | | 3.520 | | 0.532 | B | |
| *D. desulfuricans G20* | NC_007519 | | | 1.039 | 0.962 | | 0.055 | | 3.730 | | 0.422 | B | |
| *D. vulgaris* | NC_002937 | | | 1.052 | 0.973 | | 0.045 | | 3.570 | | 0.369 | A | |
| *E. canis str. Jake* | NC_007354 | | | 1.149 | 1.070 | | 0.147 | | 1.320 | | 0.710 | A | |
| *E. chaffeensis str. Arkansas* | NC_007799 | | | 1.150 | 1.091 | | 0.278 | | 1.180 | | 0.699 | A | |
| *E. ruminantium str. Gardel* | NC_006831 | | | 1.054 | 0.990 | | 0.188 | | 1.500 | | 0.725 | A | |
| *E. ruminantium* | NC_005295 | | | 1.060 | 0.996 | | 0.197 | | 1.520 | | 0.725 | A | |
| *E. ruminantium* | NC_006832 | | | 1.060 | 0.996 | | 0.196 | | 1.510 | | 0.725 | A | |
| *E. faecalis V583* | NC_004668 | | | 0.930 | 0.858 | | 0.085 | | 3.220 | | 0.625 | A | |
| *E. carotovora* | NC_004547 | | | 1.083 | 1.005 | | 0.050 | | 5.060 | | 0.490 | B | |
| *E. litoralis HTCC2594* | NC_007722 | | | 1.097 | 1.018 | | 0.036 | | 3.050 | | 0.369 | D | |
| *E. coli CFT073* | NC_004431 | | | 1.037 | 0.962 | | 0.040 | | 5.230 | | 0.495 | C | |
| *E. coli K12* | NC_000913 | | | 1.016 | 0.943 | | 0.038 | | 4.640 | | 0.492 | C | |
| *E. coli O157:H7 EDL933* | NC_002655 | | | 1.041 | 0.967 | | 0.031 | | 5.520 | | 0.496 | B | |
| *E. coli O157:H7 str. Sakai* | NC_002695 | | | 1.040 | 0.966 | | 0.037 | | 5.500 | | 0.495 | C | |
| *E. coli UTI89* | NC_007946 | | | 1.028 | 0.955 | | 0.051 | | 5.070 | | 0.494 | C | |
| *E. coli W3110* | AC_000091 | | | 1.017 | 0.944 | | 0.034 | | 4.650 | | 0.492 | C | |
| *F. tularensis* | NC_007880 | | | 1.204 | 1.123 | | 0.150 | | 1.900 | | 0.678 | B | |
| *F. tularensis* | NC_006570 | | | 1.200 | 1.118 | | 0.150 | | 1.890 | | 0.677 | B | |
| *Frankia sp. CcI3* | NC_007777 | | | 1.212 | 1.119 | | 0.047 | | 5.430 | | 0.299 | C | |
| *F. nucleatum* | NC_003454 | | | 0.931 | 0.884 | | 0.206 | | 2.170 | | 0.728 | B | |
| *G. kaustophilus HTA426* | NC_006510 | | | 0.932 | 0.861 | | 0.045 | | 3.540 | | 0.479 | A | |
| *G. metallireducens GS-15* | NC_007517 | | | 0.979 | 0.909 | | 0.077 | | 4.000 | | 0.405 | C | |
| *G. sulfurreducens PCA* | NC_002939 | | | 1.074 | 0.994 | | 0.063 | | 3.810 | | 0.391 | B | |
| *G. violaceus PCC 7421* | NC_005125 | | | 1.027 | 0.952 | | 0.043 | | 4.660 | | 0.380 | D | |
| *G. oxydans 621H* | NC_006677 | | | 1.141 | 1.065 | | 0.136 | | 2.700 | | 0.389 | C | |
| *H. ducreyi 35000HP* | NC_002940 | | | 0.821 | 0.794 | | 0.250 | | 1.700 | | 0.618 | B | |
| *H. influenzae 86-028NP* | NC_007146 | | | 0.889 | 0.822 | | 0.046 | | 1.910 | | 0.618 | C | |
| *H. influenzae Rd KW20* | NC_000907 | | | 0.880 | 0.815 | | 0.064 | | 1.830 | | 0.618 | C | |
| *H. chejuensis KCTC 2396* | NC_007645 | | | 1.077 | 0.997 | | 0.048 | | 7.220 | | 0.461 | A | |
| *H. acinonychis str. Sheeba* | NC_008229 | | | 1.039 | 0.961 | | 0.075 | | 1.550 | | 0.618 | C | |
| *H. hepaticus ATCC 51449* | NC_004917 | | | 1.178 | 1.093 | | 0.107 | | 1.800 | | 0.641 | C | |
| *H. pylori 26695* | NC_000915 | | | 1.017 | 0.942 | | 0.090 | | 1.670 | | 0.611 | C | |
| *H. pylori HPAG1* | NC_008086 | | | 1.018 | 0.941 | | 0.051 | | 1.600 | | 0.609 | C | |
| *H. pylori J99* | NC_000921 | | | 1.010 | 0.934 | | 0.065 | | 1.640 | | 0.608 | C | |
| *I. loihiensis L2TR* | NC_006512 | | | 0.770 | 0.758 | | 0.277 | | 2.840 | | 0.530 | C | |
| *Jannaschia sp. CCS1* | NC_007802 | | | 1.211 | 1.128 | | 0.112 | | 4.320 | | 0.377 | D | |
| *L. acidophilus NCFM* | NC_006814 | | | 1.066 | 0.995 | | 0.169 | | 1.990 | | 0.653 | A | |
| *L. delbrueckii* | NC_008054 | | | 0.938 | 0.866 | | 0.067 | | 1.860 | | 0.503 | B | |
| *L. johnsonii NCC 533* | NC_005362 | | | 1.104 | 1.027 | | 0.161 | | 1.990 | | 0.654 | A | |
| *L. plantarum WCFS1* | NC_004567 | | | 0.679 | 0.630 | | 0.059 | | 3.310 | | 0.555 | A | |
| *L. sakei subsp. sakei 23K* | NC_007576 | | | 0.742 | 0.689 | | 0.069 | | 1.880 | | 0.587 | A | |
| *L. salivarius* | NC_007929 | | | 1.092 | 1.012 | | 0.119 | | 1.830 | | 0.671 | A | |
| *L. lactis subsp. lactis Il1403* | NC_002662 | | | 1.100 | 1.017 | | 0.091 | | 2.370 | | 0.647 | A | |
| *L. intracellularis PHE/MN1-00* | NC_008011 | | | 1.053 | 0.977 | | 0.107 | | 1.460 | | 0.667 | A | |
| *L. pneumophila str. Lens* | NC_006369 | | | 1.260 | 1.169 | | 0.083 | | 3.350 | | 0.616 | B | |
| *L. pneumophila str. Paris* | NC_006368 | | | 1.260 | 1.168 | | 0.062 | | 3.500 | | 0.616 | A | |
| *L. pneumophila* | NC_002942 | | | 1.259 | 1.167 | | 0.052 | | 3.400 | | 0.617 | A | |
| *L. xyli* | NC_006087 | | | 1.097 | 1.015 | | 0.061 | | 2.580 | | 0.323 | C | |
| *L. interrogans* | NC_005823 | | | 0.939 | 0.873 | | 0.042 | | 4.280 | | 0.650 | A | |
| *L. interrogans* | NC_005824 | | | 0.917 | 0.856 | | 0.141 | | 0.350 | | 0.650 | B | |
| *L. interrogans* | NC_004342 | | | 0.937 | 0.872 | | 0.062 | | 4.330 | | 0.650 | B | |
| *L. interrogans* | NC_004343 | | | 0.918 | 0.857 | | 0.145 | | 0.360 | | 0.649 | B | |
| *L. innocua Clip11262* | NC_003212 | | | 0.873 | 0.810 | | 0.106 | | 3.010 | | 0.626 | A | |
| *L. monocytogenes EGD-e* | NC_003210 | | | 0.885 | 0.821 | | 0.096 | | 2.940 | | 0.620 | A | |
| *L. monocytogenes* | NC_002973 | | | 0.881 | 0.817 | | 0.095 | | 2.910 | | 0.620 | A | |
| *M. magneticum AMB-1* | NC_007626 | | | 1.208 | 1.119 | | 0.095 | | 4.970 | | 0.349 | C | |
| *M. succiniciproducens MBEL55E* | NC_006300 | | | 0.733 | 0.684 | | 0.114 | | 2.310 | | 0.575 | C | |
| *M. florum L1* | NC_006055 | | | 1.111 | 1.029 | | 0.105 | | 0.790 | | 0.730 | A | |
| *M. loti MAFF303099* | NC_002678 | | | 1.130 | 1.048 | | 0.058 | | 7.040 | | 0.373 | D | |
| *M. flagellatus KT* | NC_007947 | | | 1.286 | 1.191 | | 0.052 | | 2.970 | | 0.443 | B | |
| *M. capsulatus* | NC_002977 | | | 1.121 | 1.039 | | 0.049 | | 3.300 | | 0.364 | B | |
| *M. thermoacetica ATCC 39073* | NC_007644 | | | 0.826 | 0.764 | | 0.068 | | 2.630 | | 0.442 | B | |
| *M. avium* | NC_002944 | | | 1.086 | 1.005 | | 0.042 | | 4.830 | | 0.307 | D | |
| *M. bovis AF2122/97* | NC_002945 | | | 1.084 | 1.006 | | 0.060 | | 4.350 | | 0.344 | C | |
| *M. leprae TN* | NC_002677 | | | 0.930 | 0.865 | | 0.087 | | 3.270 | | 0.422 | A | |
| *Mycobacterium sp. MCS* | NC_008146 | | | 1.188 | 1.097 | | 0.030 | | 5.710 | | 0.315 | C | |
| *M. tuberculosis CDC1551* | NC_002755 | | | 1.085 | 1.006 | | 0.059 | | 4.400 | | 0.344 | C | |
| *M. tuberculosis H37Rv* | NC_000962 | | | 1.085 | 1.007 | | 0.057 | | 4.410 | | 0.344 | C | |
| *M. capricolum* | NC_007633 | | | 1.059 | 0.984 | | 0.142 | | 1.010 | | 0.762 | B | |
| *M. gallisepticum R* | NC_004829 | | | 0.930 | 0.862 | | 0.118 | | 1.000 | | 0.685 | A | |
| *M. genitalium G37* | NC_000908 | | | 0.963 | 0.898 | | 0.165 | | 0.580 | | 0.683 | C | |
| *M. hyopneumoniae 232* | NC_006360 | | | 0.950 | 0.881 | | 0.136 | | 0.890 | | 0.714 | C | |
| *M. hyopneumoniae 7448* | NC_007332 | | | 0.962 | 0.893 | | 0.137 | | 0.920 | | 0.715 | C | |
| *M. hyopneumoniae J* | NC_007295 | | | 0.963 | 0.892 | | 0.124 | | 0.900 | | 0.715 | C | |
| *M. mobile 163K* | NC_006908 | | | 1.100 | 1.020 | | 0.106 | | 0.780 | | 0.750 | C | |
| *M. mycoides* | NC_005364 | | | 1.054 | 0.979 | | 0.137 | | 1.210 | | 0.760 | C | |
| *M. penetrans HF-2* | NC_004432 | | | 1.042 | 0.968 | | 0.114 | | 1.360 | | 0.743 | A | |
| *M. pneumoniae M129* | NC_000912 | | | 0.742 | 0.700 | | 0.187 | | 0.820 | | 0.600 | C | |
| *M. pulmonis UAB CTIP* | NC_002771 | | | 1.081 | 1.005 | | 0.122 | | 0.960 | | 0.734 | C | |
| *M. synoviae 53* | NC_007294 | | | 0.990 | 0.937 | | 0.240 | | 0.800 | | 0.715 | C | |
| *M. xanthus DK 1622* | NC_008095 | | | 1.038 | 0.955 | | 0.047 | | 9.140 | | 0.311 | B | |
| *N. gonorrhoeae FA 1090* | NC_002946 | | | 0.756 | 0.700 | | 0.052 | | 2.150 | | 0.473 | C | |
| *N. meningitidis MC58* | NC_003112 | | | 0.761 | 0.706 | | 0.075 | | 2.270 | | 0.485 | C | |
| *N. meningitidis Z2491* | NC_003116 | | | 0.761 | 0.705 | | 0.060 | | 2.180 | | 0.482 | C | |
| *N. sennetsu str. Miyayama* | NC_007798 | | | 1.098 | 1.020 | | 0.142 | | 0.860 | | 0.589 | A | |
| *N. hamburgensis X14* | NC_007964 | | | 1.126 | 1.044 | | 0.027 | | 4.410 | | 0.383 | C | |
| *N. winogradskyi Nb-255* | NC_007406 | | | 1.122 | 1.041 | | 0.039 | | 3.400 | | 0.380 | C | |
| *N. oceani ATCC 19707* | NC_007484 | | | 1.078 | 0.998 | | 0.075 | | 3.480 | | 0.497 | A | |
| *N. europaea ATCC 19718* | NC_004757 | | | 1.280 | 1.198 | | 0.167 | | 2.810 | | 0.493 | C | |
| *N. multiformis ATCC 25196* | NC_007614 | | | 1.224 | 1.136 | | 0.050 | | 3.180 | | 0.461 | C | |
| *N. farcinica IFM 10152* | NC_006361 | | | 1.164 | 1.075 | | 0.036 | | 6.020 | | 0.292 | C | |
| *Nostoc sp. PCC 7120* | NC_003272 | | | 1.057 | 0.978 | | 0.043 | | 6.410 | | 0.587 | D | |
| *N. aromaticivorans* | NC_007794 | | | 1.087 | 1.006 | | 0.042 | | 3.560 | | 0.348 | C | |
| *O. iheyensis HTE831* | NC_004193 | | | 1.075 | 0.995 | | 0.075 | | 3.630 | | 0.643 | A | |
| *O. yellows phytoplasma OY-M* | NC_005303 | | | 0.849 | 0.798 | | 0.217 | | 0.860 | | 0.723 | C | |
| *P. multocida* | NC_002663 | | | 0.864 | 0.807 | | 0.128 | | 2.260 | | 0.596 | C | |
| *P. carbinolicus DSM 2380* | NC_007498 | | | 1.088 | 1.008 | | 0.061 | | 3.670 | | 0.449 | A | |
| *P. luteolum DSM 273* | NC_007512 | | | 1.088 | 1.011 | | 0.100 | | 2.360 | | 0.427 | B | |
| *P. profundum SS9* | NC_006370 | | | 0.967 | 0.898 | | 0.060 | | 4.090 | | 0.580 | A | |
| *P. profundum SS9* | NC_006371 | | | 1.018 | 0.944 | | 0.077 | | 2.240 | | 0.588 | B | |
| *P. luminescens* | NC_005126 | | | 1.166 | 1.083 | | 0.132 | | 5.690 | | 0.572 | B | |
| *Polaromonas sp. JS666* | NC_007948 | | | 1.103 | 1.022 | | 0.033 | | 5.200 | | 0.375 | C | |
| *P. gingivalis W83* | NC_002950 | | | 0.955 | 0.885 | | 0.061 | | 2.340 | | 0.517 | C | |
| *P. marinus str. MIT 9312* | NC_007577 | | | 0.987 | 0.918 | | 0.061 | | 1.710 | | 0.688 | C | |
| *P. marinus str. MIT 9313* | NC_005071 | | | 1.241 | 1.188 | | 0.340 | | 2.410 | | 0.493 | B | |
| *P. marinus str. NATL2A* | NC_007335 | | | 1.055 | 0.979 | | 0.063 | | 1.840 | | 0.649 | B | |
| *P. marinus subsp. marinus* | NC_005042 | | | 1.116 | 1.037 | | 0.059 | | 1.750 | | 0.636 | A | |
| *P. marinus subsp. pastoris* | NC_005072 | | | 0.942 | 0.877 | | 0.074 | | 1.660 | | 0.692 | C | |
| *P. acnes KPA171202* | NC_006085 | | | 1.225 | 1.133 | | 0.065 | | 2.560 | | 0.400 | A | |
| *P. atlantica T6c* | NC_008228 | | | 0.944 | 0.876 | | 0.039 | | 5.190 | | 0.554 | B | |
| *P. haloplanktis TAC125* | NC_007481 | | | 0.954 | 0.886 | | 0.041 | | 3.210 | | 0.598 | B | |
| *P. haloplanktis TAC125* | NC_007482 | | | 0.962 | 0.908 | | 0.225 | | 0.640 | | 0.606 | D | |
| *P. aeruginosa PAO1* | NC_002516 | | | 1.039 | 0.964 | | 0.101 | | 6.260 | | 0.334 | C | |
| *P. entomophila L48* | NC_008027 | | | 1.093 | 1.010 | | 0.037 | | 5.890 | | 0.358 | C | |
| *P. fluorescens Pf-5* | NC_004129 | | | 1.157 | 1.070 | | 0.061 | | 7.070 | | 0.367 | C | |
| *P. fluorescens PfO-1* | NC_007492 | | | 1.056 | 0.980 | | 0.043 | | 6.440 | | 0.395 | C | |
| *P. putida KT2440* | NC_002947 | | | 1.088 | 1.008 | | 0.057 | | 6.180 | | 0.385 | C | |
| *P. syringae pv. phaseolicola* | NC_005773 | | | 1.089 | 1.010 | | 0.041 | | 5.930 | | 0.420 | C | |
| *P. syringae pv. syringae* | NC_007005 | | | 1.102 | 1.021 | | 0.037 | | 6.090 | | 0.408 | C | |
| *P. syringae pv. tomato* | NC_004578 | | | 1.068 | 0.991 | | 0.043 | | 6.400 | | 0.416 | C | |
| *P. arcticus 273-4* | NC_007204 | | | 1.059 | 0.981 | | 0.057 | | 2.650 | | 0.572 | C | |
| *P. cryohalolentis K5* | NC_007969 | | | 1.077 | 0.996 | | 0.050 | | 3.060 | | 0.577 | C | |
| *R. eutropha JMP134* | NC_007348 | | | 1.108 | 1.028 | | 0.068 | | 2.730 | | 0.350 | D | |
| *R. eutropha JMP134* | NC_007347 | | | 1.085 | 1.005 | | 0.059 | | 3.810 | | 0.353 | C | |
| *R. metallidurans CH34* | NC_007973 | | | 1.123 | 1.040 | | 0.062 | | 3.930 | | 0.362 | C | |
| *R. metallidurans CH34* | NC_007974 | | | 1.149 | 1.067 | | 0.077 | | 2.580 | | 0.364 | C | |
| *R. solanacearum GMI1000* | NC_003295 | | | 1.083 | 1.003 | | 0.071 | | 3.720 | | 0.330 | C | |
| *R. etli CFN 42* | NC_007761 | | | 1.093 | 1.017 | | 0.082 | | 4.380 | | 0.387 | D | |
| *R. sphaeroides 2.4.1* | NC_007493 | | | 1.080 | 1.003 | | 0.061 | | 3.190 | | 0.310 | D | |
| *R. sphaeroides 2.4.1* | NC_007494 | | | 1.083 | 1.007 | | 0.082 | | 0.940 | | 0.310 | D | |
| *R. ferrireducens T118* | NC_007908 | | | 1.063 | 0.986 | | 0.034 | | 4.710 | | 0.401 | C | |
| *R. baltica SH 1* | NC_005027 | | | 1.089 | 1.009 | | 0.060 | | 7.150 | | 0.446 | C | |
| *R. palustris BisB18* | NC_007925 | | | 1.127 | 1.045 | | 0.030 | | 5.510 | | 0.350 | D | |
| *R. palustris BisB5* | NC_007958 | | | 1.131 | 1.049 | | 0.033 | | 4.890 | | 0.352 | D | |
| *R. palustris CGA009* | NC_005296 | | | 1.118 | 1.036 | | 0.031 | | 5.460 | | 0.350 | C | |
| *R. palustris HaA2* | NC_007778 | | | 1.139 | 1.056 | | 0.031 | | 5.330 | | 0.340 | D | |
| *R. rubrum ATCC 11170* | NC_007643 | | | 1.122 | 1.042 | | 0.024 | | 4.350 | | 0.346 | A | |
| *R. bellii RML369-C* | NC_007940 | | | 1.089 | 1.023 | | 0.180 | | 1.520 | | 0.684 | C | |
| *R. conorii str. Malish 7* | NC_003103 | | | 0.992 | 0.924 | | 0.112 | | 1.270 | | 0.676 | B | |
| *R. felis URRWXCal2* | NC_007109 | | | 0.939 | 0.881 | | 0.148 | | 1.490 | | 0.675 | C | |
| *R. prowazekii str. Madrid E* | NC_000963 | | | 1.187 | 1.107 | | 0.156 | | 1.110 | | 0.710 | A | |
| *R. typhi str. Wilmington* | NC_006142 | | | 1.195 | 1.118 | | 0.187 | | 1.110 | | 0.711 | B | |
| *R. xylanophilus DSM 9941* | NC_008148 | | | 0.686 | 0.646 | | 0.145 | | 3.230 | | 0.295 | C | |
| *S. degradans 2-40* | NC_007912 | | | 0.836 | 0.774 | | 0.035 | | 5.060 | | 0.542 | A | |
| *S. ruber DSM 13855* | NC_007677 | | | 1.093 | 1.009 | | 0.043 | | 3.550 | | 0.338 | B | |
| *S. enterica* | NC_006905 | | | 1.011 | 0.937 | | 0.043 | | 4.760 | | 0.478 | B | |
| *S. enterica* | NC_006511 | | | 1.009 | 0.935 | | 0.045 | | 4.590 | | 0.478 | B | |
| *S. enterica* | NC_003198 | | | 1.017 | 0.942 | | 0.033 | | 4.810 | | 0.479 | B | |
| *S. enterica* | NC_004631 | | | 1.016 | 0.942 | | 0.050 | | 4.790 | | 0.479 | C | |
| *S. typhimurium LT2* | NC_003197 | | | 1.014 | 0.939 | | 0.045 | | 4.860 | | 0.478 | C | |
| *S. denitrificans OS217* | NC_007954 | | | 1.194 | 1.106 | | 0.049 | | 4.550 | | 0.549 | B | |
| *S. oneidensis MR-1* | NC_004347 | | | 1.050 | 0.972 | | 0.044 | | 4.970 | | 0.540 | A | |
| *S. boydii Sb227* | NC_007613 | | | 1.027 | 0.953 | | 0.045 | | 4.520 | | 0.488 | D | |
| *S. dysenteriae Sd197* | NC_007606 | | | 1.016 | 0.944 | | 0.073 | | 4.370 | | 0.488 | D | |
| *S. flexneri 2a str. 2457T* | NC_004741 | | | 1.027 | 0.953 | | 0.042 | | 4.600 | | 0.491 | C | |
| *S. flexneri 2a str. 301* | NC_004337 | | | 1.026 | 0.953 | | 0.045 | | 4.610 | | 0.491 | C | |
| *S. sonnei Ss046* | NC_007384 | | | 1.032 | 0.958 | | 0.036 | | 4.830 | | 0.490 | C | |
| *S. pomeroyi DSS-3* | NC_003911 | | | 1.200 | 1.113 | | 0.038 | | 4.110 | | 0.358 | C | |
| *S. sp. TM1040* | NC_008044 | | | 1.208 | 1.120 | | 0.044 | | 3.200 | | 0.396 | C | |
| *S. meliloti 1021* | NC_003047 | | | 1.062 | 0.986 | | 0.042 | | 3.650 | | 0.373 | D | |
| *S. glossinidius* | NC_007712 | | | 1.064 | 0.987 | | 0.085 | | 4.170 | | 0.453 | C | |
| *S. alaskensis RB2256* | NC_008048 | | | 1.123 | 1.041 | | 0.050 | | 3.350 | | 0.345 | D | |
| *S. aureus RF122* | NC_007622 | | | 0.989 | 0.916 | | 0.074 | | 2.740 | | 0.672 | A | |
| *S. aureus subsp. aureus COL* | NC_002951 | | | 0.997 | 0.924 | | 0.096 | | 2.810 | | 0.672 | A | |
| *S. aureus subsp. aureus MRSA252* | NC_002952 | | | 0.997 | 0.926 | | 0.104 | | 2.900 | | 0.672 | A | |
| *S. aureus subsp. aureus MSSA476* | NC_002953 | | | 0.992 | 0.920 | | 0.095 | | 2.800 | | 0.671 | A | |
| *S. aureus subsp. aureus Mu50* | NC_002758 | | | 0.990 | 0.918 | | 0.086 | | 2.880 | | 0.671 | A | |
| *S. aureus subsp. aureus MW2* | NC_003923 | | | 0.993 | 0.922 | | 0.100 | | 2.820 | | 0.672 | A | |
| *S. aureus subsp. aureus N315* | NC_002745 | | | 0.993 | 0.921 | | 0.095 | | 2.810 | | 0.672 | A | |
| *S. aureus subsp. aureus NCTC 8325* | NC_007795 | | | 0.989 | 0.922 | | 0.135 | | 2.820 | | 0.671 | A | |
| *S. aureus subsp. aureus USA300* | NC_007793 | | | 0.998 | 0.927 | | 0.103 | | 2.870 | | 0.672 | A | |
| *S. epidermidis ATCC 12228* | NC_004461 | | | 1.084 | 1.021 | | 0.239 | | 2.500 | | 0.679 | A | |
| *S. epidermidis RP62A* | NC_002976 | | | 1.068 | 1.023 | | 0.304 | | 2.620 | | 0.678 | A | |
| *S. haemolyticus JCSC1435* | NC_007168 | | | 1.009 | 0.938 | | 0.152 | | 2.690 | | 0.672 | A | |
| *S. saprophyticus* | NC_007350 | | | 1.023 | 0.949 | | 0.100 | | 2.520 | | 0.668 | A | |
| *S. agalactiae 2603V/R* | NC_004116 | | | 1.108 | 1.026 | | 0.105 | | 2.160 | | 0.644 | A | |
| *S. agalactiae A909* | NC_007432 | | | 1.103 | 1.025 | | 0.146 | | 2.130 | | 0.644 | A | |
| *S. agalactiae NEM316* | NC_004368 | | | 1.107 | 1.025 | | 0.093 | | 2.210 | | 0.644 | A | |
| *S. mutans UA159* | NC_004350 | | | 1.144 | 1.060 | | 0.087 | | 2.030 | | 0.632 | A | |
| *S. pneumoniae R6* | NC_003098 | | | 1.032 | 0.956 | | 0.072 | | 2.040 | | 0.603 | A | |
| *S. pneumoniae TIGR4* | NC_003028 | | | 1.039 | 0.963 | | 0.118 | | 2.160 | | 0.603 | A | |
| *S. pyogenes M1 GAS* | NC_002737 | | | 1.154 | 1.072 | | 0.132 | | 1.850 | | 0.615 | A | |
| *S. pyogenes MGAS10270* | NC_008022 | | | 1.149 | 1.064 | | 0.088 | | 1.930 | | 0.616 | A | |
| *S. pyogenes MGAS10394* | NC_006086 | | | 1.153 | 1.069 | | 0.092 | | 1.900 | | 0.613 | A | |
| *S. pyogenes MGAS10750* | NC_008024 | | | 1.144 | 1.067 | | 0.161 | | 1.940 | | 0.617 | A | |
| *S. pyogenes MGAS2096* | NC_008023 | | | 1.154 | 1.069 | | 0.088 | | 1.860 | | 0.613 | A | |
| *S. pyogenes MGAS315* | NC_004070 | | | 1.158 | 1.075 | | 0.113 | | 1.900 | | 0.614 | A | |
| *S. pyogenes MGAS5005* | NC_007297 | | | 1.156 | 1.071 | | 0.091 | | 1.840 | | 0.615 | A | |
| *S. pyogenes MGAS6180* | NC_007296 | | | 1.150 | 1.067 | | 0.113 | | 1.900 | | 0.616 | A | |
| *S. pyogenes MGAS8232* | NC_003485 | | | 1.159 | 1.073 | | 0.085 | | 1.900 | | 0.615 | A | |
| *S. pyogenes MGAS9429* | NC_008021 | | | 1.153 | 1.071 | | 0.130 | | 1.840 | | 0.615 | A | |
| *S. pyogenes SSI-1* | NC_004606 | | | 1.156 | 1.077 | | 0.147 | | 1.890 | | 0.614 | A | |
| *S. thermophilus CNRZ1066* | NC_006449 | | | 1.041 | 0.966 | | 0.104 | | 1.800 | | 0.609 | A | |
| *S. thermophilus LMG 18311* | NC_006448 | | | 1.042 | 0.967 | | 0.104 | | 1.800 | | 0.609 | A | |
| *S. avermitilis MA-4680* | NC_003155 | | | 1.089 | 1.002 | | 0.065 | | 9.030 | | 0.293 | D | |
| *S. coelicolor A3(2)* | NC_003888 | | | 1.062 | 0.976 | | 0.064 | | 8.670 | | 0.279 | C | |
| *S. thermophilum IAM 14863* | NC_006177 | | | 1.041 | 0.965 | | 0.111 | | 3.570 | | 0.313 | A | |
| *S. elongatus PCC 6301* | NC_006576 | | | 1.069 | 0.990 | | 0.035 | | 2.700 | | 0.445 | D | |
| *S. elongatus PCC 7942* | NC_007604 | | | 1.069 | 0.990 | | 0.036 | | 2.700 | | 0.445 | C | |
| *Synechococcus sp. CC9605* | NC_007516 | | | 1.249 | 1.155 | | 0.045 | | 2.510 | | 0.408 | B | |
| *Synechococcus sp. CC9902* | NC_007513 | | | 1.190 | 1.101 | | 0.058 | | 2.230 | | 0.458 | A | |
| *Synechococcus sp. JA-2-3B'a(2-13)* | NC_007776 | | | 1.148 | 1.062 | | 0.039 | | 3.050 | | 0.415 | D | |
| *Synechococcus sp. JA-3-3Ab* | NC_007775 | | | 1.129 | 1.044 | | 0.034 | | 2.930 | | 0.398 | D | |
| *Synechococcus sp. WH 8102* | NC_005070 | | | 1.251 | 1.157 | | 0.058 | | 2.430 | | 0.406 | B | |
| *Synechocystis sp. PCC 6803* | NC_000911 | | | 0.865 | 0.800 | | 0.030 | | 3.570 | | 0.523 | D | |
| *S. aciditrophicus SB* | NC_007759 | | | 1.060 | 0.987 | | 0.086 | | 3.180 | | 0.485 | C | |
| *T. tengcongensis MB4* | NC_003869 | | | 0.892 | 0.828 | | 0.049 | | 2.690 | | 0.624 | A | |
| *T. fusca YX* | NC_007333 | | | 1.048 | 0.965 | | 0.056 | | 3.640 | | 0.325 | C | |
| *T. elongatus BP-1* | NC_004113 | | | 1.166 | 1.078 | | 0.042 | | 2.590 | | 0.461 | D | |
| *T. maritima MSB8* | NC_000853 | | | 0.817 | 0.768 | | 0.147 | | 1.860 | | 0.538 | D | |
| *T. thermophilus HB27* | NC_005835 | | | 0.496 | 0.467 | | 0.079 | | 1.890 | | 0.306 | C | |
| *T. thermophilus HB8* | NC_006461 | | | 0.496 | 0.475 | | 0.117 | | 1.850 | | 0.305 | D | |
| *T. denitrificans ATCC 25259* | NC_007404 | | | 1.022 | 0.946 | | 0.041 | | 2.910 | | 0.339 | D | |
| *T. crunogena XCL-2* | NC_007520 | | | 0.936 | 0.871 | | 0.131 | | 2.430 | | 0.569 | A | |
| *T. denitrificans ATCC 33889* | NC_007575 | | | 1.070 | 0.994 | | 0.083 | | 2.200 | | 0.655 | C | |
| *T. denticola ATCC 35405* | NC_002967 | | | 0.739 | 0.686 | | 0.090 | | 2.840 | | 0.621 | C | |
| *T. pallidum subsp. pallidum* | NC_000919 | | | 0.827 | 0.770 | | 0.108 | | 1.140 | | 0.472 | A | |
| *T. whipplei str. Twist* | NC_004572 | | | 1.207 | 1.128 | | 0.190 | | 0.930 | | 0.537 | B | |
| *T. whipplei TW08/27* | NC_004551 | | | 1.207 | 1.126 | | 0.170 | | 0.930 | | 0.537 | B | |
| *U. parvum serovar 3* | NC_002162 | | | 0.989 | 0.920 | | 0.179 | | 0.750 | | 0.745 | B | |
| *V. cholerae O1 biovar eltor* | NC_002505 | | | 0.991 | 0.920 | | 0.073 | | 2.960 | | 0.523 | B | |
| *V. cholerae O1 biovar eltor* | NC_002506 | | | 0.985 | 0.917 | | 0.148 | | 1.070 | | 0.531 | C | |
| *V. fischeri ES114* | NC_006840 | | | 0.983 | 0.910 | | 0.061 | | 2.910 | | 0.610 | A | |
| *V. fischeri ES114* | NC_006841 | | | 1.058 | 0.985 | | 0.151 | | 1.330 | | 0.630 | A | |
| *V. parahaemolyticus RIMD 2210633* | NC_004603 | | | 0.762 | 0.707 | | 0.065 | | 3.290 | | 0.546 | B | |
| *V. parahaemolyticus RIMD 2210633* | NC_004605 | | | 0.791 | 0.734 | | 0.063 | | 1.880 | | 0.546 | B | |
| *V. vulnificus CMCP6* | NC_004460 | | | 0.942 | 0.873 | | 0.049 | | 1.840 | | 0.529 | B | |
| *V. vulnificus CMCP6* | NC_004459 | | | 0.905 | 0.839 | | 0.077 | | 3.280 | | 0.536 | B | |
| *V. vulnificus YJ016* | NC_005139 | | | 0.905 | 0.838 | | 0.059 | | 3.350 | | 0.536 | A | |
| *V. vulnificus YJ016* | NC_005140 | | | 0.937 | 0.869 | | 0.061 | | 1.860 | | 0.528 | B | |
| *W. glossinidia endosymbiont* | NC_004344 | | | 1.044 | 0.970 | | 0.095 | | 0.700 | | 0.775 | C | |
| *W. endosymbiont* | NC_002978 | | | 1.199 | 1.113 | | 0.093 | | 1.270 | | 0.648 | C | |
| *W. endosymbiont* | NC_006833 | | | 1.180 | 1.129 | | 0.311 | | 1.080 | | 0.658 | C | |
| *W. succinogenes DSM 1740* | NC_005090 | | | 0.956 | 0.889 | | 0.074 | | 2.110 | | 0.515 | B | |
| *X. axonopodis pv. citri* | NC_003919 | | | 1.110 | 1.029 | | 0.033 | | 5.180 | | 0.352 | C | |
| *X. campestris pv. campestris* | NC_007086 | | | 1.102 | 1.022 | | 0.035 | | 5.150 | | 0.350 | C | |
| *X. campestris pv. campestris* | NC_003902 | | | 1.101 | 1.021 | | 0.038 | | 5.080 | | 0.349 | C | |
| *X. campestris pv. vesicatoria* | NC_007508 | | | 1.109 | 1.028 | | 0.032 | | 5.180 | | 0.353 | C | |
| *X. oryzae pv. oryzae KACC10331* | NC_006834 | | | 1.102 | 1.022 | | 0.031 | | 4.940 | | 0.363 | C | |
| *X. oryzae pv. oryzae MAFF 311018* | NC_007705 | | | 1.101 | 1.021 | | 0.043 | | 4.940 | | 0.363 | C | |
| *X. fastidiosa 9a5c* | NC_002488 | | | 1.009 | 1.052 | | 0.517 | | 2.680 | | 0.473 | A | |
| *X. fastidiosa Temecula1* | NC_004556 | | | 1.064 | 0.993 | | 0.127 | | 2.520 | | 0.482 | A | |
| *Y. pestis Antiqua* | NC_008150 | | | 1.099 | 1.018 | | 0.038 | | 4.700 | | 0.523 | B | |
| *Y. pestis biovar Microtus* | NC_005810 | | | 1.094 | 1.013 | | 0.046 | | 4.600 | | 0.523 | C | |
| *Y. pestis CO92* | NC_003143 | | | 1.096 | 1.016 | | 0.070 | | 4.650 | | 0.524 | C | |
| *Y. pestis KIM* | NC_004088 | | | 1.094 | 1.014 | | 0.067 | | 4.600 | | 0.524 | C | |
| *Y. pestis Nepal516* | NC_008149 | | | 1.092 | 1.013 | | 0.081 | | 4.530 | | 0.524 | B | |
| *Y. pseudotuberculosis IP 32953* | NC_006155 | | | 1.084 | 1.004 | | 0.063 | | 4.740 | | 0.524 | B | |
| *Z. mobilis subsp. mobilis ZM4* | NC_006526 | | | 0.973 | 0.931 | | 0.279 | | 2.060 | | 0.537 | C | |
| **106 Unicellular chromosomes** | | **Accession no.** | | ***χ_r_*,*_gl_*** | | ***χ_c_*,*_gl_*** | | ***χ_i_*,*_gl_*** | | ***L* (Mb)** | ***p*** | **Type** | |
| *A. fumigatus* | | NC_007194 | | 1.119 | | 1.034 | | 0.056 | | 4.880 | 0.501 | D | |
| *A. fumigatus* | | NC_007195 | | 1.100 | | 1.016 | | 0.066 | | 4.820 | 0.501 | D | |
| *A. fumigatus* | | NC_007196 | | 1.141 | | 1.054 | | 0.069 | | 4.070 | 0.504 | D | |
| *A. fumigatus* | | NC_007197 | | 1.140 | | 1.054 | | 0.060 | | 3.580 | 0.501 | D | |
| *A. fumigatus* | | NC_007198 | | 1.156 | | 1.069 | | 0.077 | | 3.890 | 0.498 | D | |
| *A. fumigatus* | | NC_007199 | | 1.124 | | 1.039 | | 0.067 | | 3.750 | 0.503 | D | |
| *A. fumigatus* | | NC_007200 | | 1.163 | | 1.075 | | 0.081 | | 2.020 | 0.505 | D | |
| *A. fumigatus* | | NC_007201 | | 1.186 | | 1.097 | | 0.082 | | 1.800 | 0.506 | C | |
| *C. albicans* | | NC_007436 | | 0.878 | | 0.811 | | 0.088 | | 0.950 | 0.665 | D | |
| *C. glabrata* | | NC_005967 | | 1.028 | | 0.957 | | 0.220 | | 0.490 | 0.601 | C | |
| *C. glabrata* | | NC_005968 | | 1.048 | | 0.976 | | 0.194 | | 0.500 | 0.611 | C | |
| *C. glabrata* | | NC_006026 | | 1.075 | | 0.997 | | 0.190 | | 0.560 | 0.603 | C | |
| *C. glabrata* | | NC_006027 | | 1.076 | | 0.997 | | 0.165 | | 0.650 | 0.609 | C | |
| *C. glabrata* | | NC_006028 | | 1.056 | | 0.980 | | 0.169 | | 0.690 | 0.610 | C | |
| *C. glabrata* | | NC_006029 | | 1.075 | | 0.996 | | 0.132 | | 0.930 | 0.619 | C | |
| *C. glabrata* | | NC_006030 | | 1.049 | | 0.972 | | 0.135 | | 0.990 | 0.614 | C | |
| *C. glabrata* | | NC_006031 | | 1.068 | | 0.988 | | 0.105 | | 1.050 | 0.619 | C | |
| *C. glabrata* | | NC_006032 | | 1.059 | | 0.979 | | 0.112 | | 1.090 | 0.615 | C | |
| *C. glabrata* | | NC_006033 | | 1.066 | | 0.987 | | 0.120 | | 1.190 | 0.612 | C | |
| *C. glabrata* | | NC_006034 | | 1.059 | | 0.983 | | 0.147 | | 1.300 | 0.618 | C | |
| *C. glabrata* | | NC_006035 | | 1.048 | | 0.972 | | 0.151 | | 1.440 | 0.617 | C | |
| *C. glabrata* | | NC_006036 | | 1.087 | | 1.006 | | 0.100 | | 1.400 | 0.615 | D | |
| *C. neoformans* | | NC_006670 | | 0.965 | | 0.892 | | 0.068 | | 2.300 | 0.514 | D | |
| *C. neoformans* | | NC_006679 | | 0.887 | | 0.821 | | 0.091 | | 1.090 | 0.512 | D | |
| *C. neoformans* | | NC_006680 | | 0.918 | | 0.849 | | 0.093 | | 1.020 | 0.514 | C | |
| *C. neoformans* | | NC_006681 | | 0.968 | | 0.896 | | 0.103 | | 0.910 | 0.516 | D | |
| *C. neoformans* | | NC_006682 | | 0.878 | | 0.818 | | 0.156 | | 0.790 | 0.514 | C | |
| *C. neoformans* | | NC_006683 | | 0.906 | | 0.838 | | 0.112 | | 0.760 | 0.513 | C | |
| *C. neoformans* | | NC_006684 | | 0.962 | | 0.891 | | 0.097 | | 1.630 | 0.516 | D | |
| *C. neoformans* | | NC_006685 | | 0.963 | | 0.889 | | 0.068 | | 2.110 | 0.516 | D | |
| *C. neoformans* | | NC_006686 | | 0.983 | | 0.909 | | 0.084 | | 1.780 | 0.515 | D | |
| *C. neoformans* | | NC_006687 | | 0.946 | | 0.876 | | 0.101 | | 1.510 | 0.513 | D | |
| *C. neoformans* | | NC_006691 | | 0.957 | | 0.885 | | 0.088 | | 1.440 | 0.516 | D | |
| *C. neoformans* | | NC_006692 | | 0.950 | | 0.883 | | 0.134 | | 1.350 | 0.515 | D | |
| *C. neoformans* | | NC_006693 | | 0.922 | | 0.853 | | 0.095 | | 1.190 | 0.514 | D | |
| *C. neoformans* | | NC_006694 | | 0.944 | | 0.874 | | 0.107 | | 1.180 | 0.515 | D | |
| *D. hansenii* | | NC_006043 | | 1.015 | | 0.948 | | 0.180 | | 1.250 | 0.634 | C | |
| *D. hansenii* | | NC_006044 | | 1.025 | | 0.953 | | 0.148 | | 1.350 | 0.633 | C | |
| *D. hansenii* | | NC_006045 | | 1.022 | | 0.945 | | 0.081 | | 1.590 | 0.636 | C | |
| *D. hansenii* | | NC_006046 | | 1.012 | | 0.937 | | 0.120 | | 1.600 | 0.636 | C | |
| *D. hansenii* | | NC_006047 | | 1.049 | | 0.971 | | 0.110 | | 2.040 | 0.643 | D | |
| *D. hansenii* | | NC_006048 | | 1.043 | | 0.967 | | 0.126 | | 2.330 | 0.636 | D | |
| *D. hansenii* | | NC_006049 | | 1.031 | | 0.959 | | 0.150 | | 2.050 | 0.639 | D | |
| *E. cuniculi* | | NC_003229 | | 0.849 | | 0.787 | | 0.123 | | 0.200 | 0.534 | B | |
| *E. cuniculi* | | NC_003230 | | 0.880 | | 0.820 | | 0.174 | | 0.190 | 0.531 | C | |
| *E. cuniculi* | | NC_003231 | | 0.877 | | 0.824 | | 0.191 | | 0.220 | 0.528 | C | |
| *E. cuniculi* | | NC_003232 | | 0.855 | | 0.840 | | 0.335 | | 0.210 | 0.533 | C | |
| *E. cuniculi* | | NC_003233 | | 0.858 | | 0.799 | | 0.141 | | 0.220 | 0.527 | C | |
| *E. cuniculi* | | NC_003234 | | 0.830 | | 0.801 | | 0.294 | | 0.230 | 0.528 | C | |
| *E. cuniculi* | | NC_003235 | | 0.858 | | 0.806 | | 0.199 | | 0.240 | 0.522 | C | |
| *E. cuniculi* | | NC_003236 | | 0.881 | | 0.815 | | 0.108 | | 0.260 | 0.527 | C | |
| *E. cuniculi* | | NC_003237 | | 0.854 | | 0.813 | | 0.245 | | 0.270 | 0.527 | C | |
| *E. cuniculi* | | NC_003238 | | 0.887 | | 0.825 | | 0.138 | | 0.250 | 0.533 | C | |
| *E. cuniculi* | | NC_003242 | | 0.898 | | 0.834 | | 0.137 | | 0.210 | 0.507 | B | |
| *E. gossypii* | | NC_005782 | | 1.014 | | 0.940 | | 0.186 | | 0.690 | 0.481 | C | |
| *E. gossypii* | | NC_005783 | | 1.051 | | 0.976 | | 0.196 | | 0.870 | 0.486 | C | |
| *E. gossypii* | | NC_005784 | | 0.986 | | 0.911 | | 0.119 | | 0.910 | 0.470 | C | |
| *E. gossypii* | | NC_005785 | | 1.026 | | 0.946 | | 0.089 | | 1.470 | 0.477 | D | |
| *E. gossypii* | | NC_005786 | | 1.029 | | 0.950 | | 0.085 | | 1.520 | 0.486 | C | |
| *E. gossypii* | | NC_005787 | | 1.019 | | 0.939 | | 0.094 | | 1.810 | 0.481 | D | |
| *E. gossypii* | | NC_005788 | | 1.026 | | 0.948 | | 0.099 | | 1.480 | 0.480 | D | |
| *K. lactis* | | NC_006037 | | 0.991 | | 0.919 | | 0.130 | | 1.060 | 0.609 | C | |
| *K. lactis* | | NC_006038 | | 0.978 | | 0.906 | | 0.112 | | 1.320 | 0.610 | C | |
| *K. lactis* | | NC_006039 | | 1.002 | | 0.931 | | 0.137 | | 1.750 | 0.616 | D | |
| *K. lactis* | | NC_006040 | | 0.988 | | 0.915 | | 0.110 | | 1.720 | 0.611 | D | |
| *K. lactis* | | NC_006041 | | 1.005 | | 0.931 | | 0.101 | | 2.230 | 0.611 | D | |
| *K. lactis* | | NC_006042 | | 1.010 | | 0.934 | | 0.076 | | 2.600 | 0.615 | D | |
| *S. cerevisiae* | | NC_001133 | | 0.961 | | 0.902 | | 0.265 | | 0.230 | 0.607 | B | |
| *S. cerevisiae* | | NC_001134 | | 1.027 | | 0.952 | | 0.196 | | 0.810 | 0.617 | C | |
| *S. cerevisiae* | | NC_001135 | | 0.974 | | 0.939 | | 0.352 | | 0.320 | 0.615 | B | |
| *S. cerevisiae* | | NC_001136 | | 1.018 | | 0.943 | | 0.123 | | 1.530 | 0.621 | C | |
| *S. cerevisiae* | | NC_001137 | | 1.013 | | 0.948 | | 0.227 | | 0.580 | 0.615 | C | |
| *S. cerevisiae* | | NC_001138 | | 1.002 | | 0.928 | | 0.207 | | 0.270 | 0.613 | B | |
| *S. cerevisiae* | | NC_001139 | | 1.021 | | 0.944 | | 0.114 | | 1.090 | 0.619 | C | |
| *S. cerevisiae* | | NC_001140 | | 1.011 | | 0.941 | | 0.182 | | 0.560 | 0.615 | C | |
| *S. cerevisiae* | | NC_001141 | | 1.015 | | 0.941 | | 0.175 | | 0.440 | 0.611 | C | |
| *S. cerevisiae* | | NC_001142 | | 1.013 | | 0.939 | | 0.161 | | 0.750 | 0.616 | C | |
| *S. cerevisiae* | | NC_001143 | | 1.020 | | 0.948 | | 0.167 | | 0.670 | 0.619 | C | |
| *S. cerevisiae* | | NC_001144 | | 1.009 | | 0.934 | | 0.129 | | 1.080 | 0.615 | C | |
| *S. cerevisiae* | | NC_001145 | | 1.004 | | 0.929 | | 0.123 | | 0.920 | 0.618 | C | |
| *S. cerevisiae* | | NC_001146 | | 0.993 | | 0.917 | | 0.129 | | 0.780 | 0.614 | C | |
| *S. cerevisiae* | | NC_001147 | | 1.001 | | 0.928 | | 0.150 | | 1.090 | 0.618 | C | |
| *S. cerevisiae* | | NC_001148 | | 1.023 | | 0.948 | | 0.120 | | 0.950 | 0.619 | C | |
| *S. pombe* | | NC_003421 | | 1.001 | | 0.923 | | 0.108 | | 2.450 | 0.638 | C | |
| *S. pombe* | | NC_003423 | | 1.009 | | 0.930 | | 0.086 | | 4.510 | 0.641 | D | |
| *S. pombe* | | NC_003424 | | 1.016 | | 0.936 | | 0.071 | | 5.570 | 0.639 | D | |
| *Y. lipolytica* | | NC_006067 | | 0.861 | | 0.794 | | 0.079 | | 2.300 | 0.513 | D | |
| *Y. lipolytica* | | NC_006068 | | 0.874 | | 0.806 | | 0.085 | | 3.070 | 0.511 | D | |
| *Y. lipolytica* | | NC_006069 | | 0.851 | | 0.785 | | 0.059 | | 3.270 | 0.514 | D | |
| *Y. lipolytica* | | NC_006070 | | 0.863 | | 0.796 | | 0.070 | | 3.630 | 0.507 | D | |
| *Y. lipolytica* | | NC_006071 | | 0.873 | | 0.804 | | 0.059 | | 4.220 | 0.507 | D | |
| *Y. lipolytica* | | NC_006072 | | 0.874 | | 0.804 | | 0.064 | | 4.000 | 0.508 | D | |
| *P. falciparum* | | NC_000521 | | 0.768 | | 0.711 | | 0.172 | | 1.060 | 0.801 | B | |
| *P. falciparum* | | NC_000910 | | 0.730 | | 0.674 | | 0.147 | | 0.950 | 0.803 | B | |
| *P. falciparum* | | NC_004314 | | 0.747 | | 0.714 | | 0.304 | | 1.690 | 0.803 | B | |
| *P. falciparum* | | NC_004315 | | 0.780 | | 0.722 | | 0.169 | | 2.040 | 0.810 | C | |
| *P. falciparum* | | NC_004316 | | 0.781 | | 0.722 | | 0.134 | | 2.270 | 0.807 | C | |
| *P. falciparum* | | NC_004317 | | 0.815 | | 0.750 | | 0.102 | | 3.290 | 0.816 | C | |
| *P. falciparum* | | NC_004318 | | 0.743 | | 0.718 | | 0.376 | | 1.200 | 0.793 | B | |
| *P. falciparum* | | NC_004325 | | 0.731 | | 0.743 | | 0.348 | | 0.640 | 0.795 | B | |
| *P. falciparum* | | NC_004326 | | 0.774 | | 0.733 | | 0.250 | | 1.340 | 0.807 | B | |
| *P. falciparum* | | NC_004327 | | 0.768 | | 0.712 | | 0.231 | | 1.420 | 0.802 | B | |
| *P. falciparum* | | NC_004328 | | 0.766 | | 0.733 | | 0.276 | | 1.350 | 0.800 | B | |
| *P. falciparum* | | NC_004329 | | 0.763 | | 0.709 | | 0.154 | | 1.320 | 0.803 | C | |
| *P. falciparum* | | NC_004330 | | 0.762 | | 0.710 | | 0.195 | | 1.540 | 0.810 | C | |
| *P. falciparum* | | NC_004331 | | 0.793 | | 0.732 | | 0.121 | | 2.730 | 0.809 | C | |
| **17 Plant chromosomes** | | **Accession no.** | | ***χ_r_*,*_gl_*** | | ***χ_c_*,*_gl_*** | | ***χ_i_*,*_gl_*** | | ***L* (Mb)** | ***p*** | **Type** | |
| *A. thaliana* | | NC_003070 | | 0.811 | | 0.747 | | 0.045 | | 30.270 | 0.641 | D | |
| *A. thaliana* | | NC_003071 | | 0.796 | | 0.734 | | 0.049 | | 19.700 | 0.641 | D | |
| *A. thaliana* | | NC_003074 | | 0.812 | | 0.748 | | 0.036 | | 23.470 | 0.637 | D | |
| *A. thaliana* | | NC_003075 | | 0.797 | | 0.734 | | 0.050 | | 18.580 | 0.638 | D | |
| *A. thaliana* | | NC_003076 | | 0.805 | | 0.742 | | 0.046 | | 26.980 | 0.641 | D | |
| *O. sativa* | | Chr01 | | 1.039 | | 0.955 | | 0.023 | | 43.270 | 0.562 | D | |
| *O. sativa* | | Chr02 | | 1.053 | | 0.968 | | 0.021 | | 35.960 | 0.567 | D | |
| *O. sativa* | | Chr03 | | 1.053 | | 0.968 | | 0.023 | | 36.320 | 0.563 | D | |
| *O. sativa* | | Chr04 | | 1.030 | | 0.947 | | 0.027 | | 35.380 | 0.557 | D | |
| *O. sativa* | | Chr05 | | 1.018 | | 0.936 | | 0.036 | | 29.870 | 0.561 | D | |
| *O. sativa* | | Chr06 | | 1.027 | | 0.944 | | 0.027 | | 31.230 | 0.564 | D | |
| *O. sativa* | | Chr07 | | 1.030 | | 0.948 | | 0.041 | | 29.730 | 0.565 | D | |
| *O. sativa* | | Chr08 | | 1.027 | | 0.945 | | 0.033 | | 28.440 | 0.566 | D | |
| *O. sativa* | | Chr09 | | 1.024 | | 0.942 | | 0.050 | | 22.950 | 0.565 | D | |
| *O. sativa* | | Chr10 | | 1.019 | | 0.938 | | 0.040 | | 22.860 | 0.564 | D | |
| *O. sativa* | | Chr11 | | 1.035 | | 0.953 | | 0.032 | | 28.470 | 0.571 | D | |
| *O. sativa* | | Chr12 | | 1.030 | | 0.948 | | 0.029 | | 27.610 | 0.570 | D | |
| **43 Insect chromosomes** | | **Accession no.** | | ***χ_r_*,*_gl_*** | | ***χ_c_*,*_gl_*** | | ***χ_i_*,*_gl_*** | | ***L* (Mb)** | ***p*** | **Type** | |
| *C. elegans* | | NC_003279 | | 1.045 | | 0.968 | | 0.065 | | 15.070 | 0.643 | D | |
| *C. elegans* | | NC_003280 | | 1.055 | | 0.978 | | 0.066 | | 15.280 | 0.638 | D | |
| *C. elegans* | | NC_003281 | | 1.052 | | 0.975 | | 0.067 | | 13.780 | 0.643 | D | |
| *C. elegans* | | NC_003282 | | 1.054 | | 0.977 | | 0.078 | | 17.490 | 0.654 | D | |
| *C. elegans* | | NC_003283 | | 1.096 | | 1.014 | | 0.048 | | 20.920 | 0.646 | D | |
| *C. elegans* | | NC_003284 | | 1.020 | | 0.941 | | 0.047 | | 17.720 | 0.648 | D | |
| *D. melanogaster* | | NC_004353 | | 0.973 | | 0.902 | | 0.150 | | 1.280 | 0.649 | C | |
| *D. melanogaster* | | NC_004354 | | 1.111 | | 1.028 | | 0.024 | | 22.220 | 0.575 | D | |
| *D. melanogaster* | | NT_033777 | | 1.137 | | 1.052 | | 0.027 | | 27.910 | 0.571 | D | |
| *D. melanogaster* | | NT_033778 | | 1.144 | | 1.059 | | 0.025 | | 20.770 | 0.567 | D | |
| *D. melanogaster* | | NT_033779 | | 1.142 | | 1.056 | | 0.021 | | 22.410 | 0.581 | D | |
| *D. melanogaster* | | NT_037436 | | 1.139 | | 1.054 | | 0.029 | | 23.770 | 0.580 | D | |
| *A. gambiae* | | NC_004818 | | 0.747 | | 0.690 | | 0.025 | | 21.480 | 0.539 | D | |
| *A. gambiae* | | NT_078265 | | 0.745 | | 0.690 | | 0.018 | | 48.070 | 0.555 | D | |
| *A. gambiae* | | NT_078266 | | 0.730 | | 0.677 | | 0.018 | | 61.230 | 0.547 | D | |
| *A. gambiae* | | NT_078267 | | 0.786 | | 0.727 | | 0.025 | | 40.050 | 0.563 | D | |
| *A. gambiae* | | NT_078268 | | 0.773 | | 0.716 | | 0.026 | | 52.290 | 0.560 | D | |
| *A. mellifera* | | Chr1 | | 1.007 | | 0.931 | | 0.018 | | 25.390 | 0.650 | D | |
| *A. mellifera* | | Chr2 | | 0.987 | | 0.913 | | 0.026 | | 13.790 | 0.628 | D | |
| *A. mellifera* | | Chr3 | | 0.989 | | 0.916 | | 0.030 | | 11.420 | 0.653 | D | |
| *A. mellifera* | | Chr4 | | 0.986 | | 0.913 | | 0.044 | | 10.830 | 0.638 | D | |
| *A. mellifera* | | Chr5 | | 0.996 | | 0.921 | | 0.024 | | 12.640 | 0.654 | D | |
| *A. mellifera* | | Chr6 | | 1.011 | | 0.935 | | 0.033 | | 14.650 | 0.661 | D | |
| *A. mellifera* | | Chr7 | | 1.011 | | 0.935 | | 0.032 | | 10.320 | 0.671 | D | |
| *A. mellifera* | | Chr8 | | 0.991 | | 0.916 | | 0.036 | | 10.750 | 0.670 | D | |
| *A. mellifera* | | Chr9 | | 1.007 | | 0.932 | | 0.028 | | 9.690 | 0.656 | D | |
| *A. mellifera* | | Chr10 | | 0.973 | | 0.900 | | 0.028 | | 10.300 | 0.639 | D | |
| *A. mellifera* | | Chr11 | | 0.997 | | 0.922 | | 0.031 | | 12.300 | 0.665 | D | |
| *A. mellifera* | | Chr12 | | 1.009 | | 0.934 | | 0.030 | | 9.730 | 0.646 | D | |
| *A. mellifera* | | Chr13 | | 0.969 | | 0.896 | | 0.023 | | 9.010 | 0.618 | D | |
| *A. mellifera* | | Chr14 | | 0.967 | | 0.893 | | 0.027 | | 8.570 | 0.647 | D | |
| *A. mellifera* | | Chr15 | | 0.986 | | 0.912 | | 0.030 | | 7.940 | 0.657 | D | |
| *A. mellifera* | | Chr16 | | 0.976 | | 0.902 | | 0.039 | | 5.980 | 0.670 | D | |
| *T. castaneum* | | Chr1 | | 0.691 | | 0.643 | | 0.079 | | 5.560 | 0.652 | C | |
| *T. castaneum* | | Chr2 | | 0.754 | | 0.700 | | 0.065 | | 9.400 | 0.641 | D | |
| *T. castaneum* | | Chr3 | | 0.716 | | 0.664 | | 0.044 | | 22.420 | 0.678 | D | |
| *T. castaneum* | | Chr4 | | 0.771 | | 0.715 | | 0.063 | | 11.540 | 0.643 | D | |
| *T. castaneum* | | Chr5 | | 0.744 | | 0.690 | | 0.054 | | 13.850 | 0.642 | D | |
| *T. castaneum* | | Chr6 | | 0.816 | | 0.754 | | 0.095 | | 8.290 | 0.671 | C | |
| *T. castaneum* | | Chr7 | | 0.731 | | 0.679 | | 0.055 | | 13.020 | 0.647 | D | |
| *T. castaneum* | | Chr8 | | 0.754 | | 0.699 | | 0.064 | | 11.500 | 0.660 | D | |
| *T. castaneum* | | Chr9 | | 0.765 | | 0.710 | | 0.074 | | 10.760 | 0.650 | D | |
| *T. castaneum* | | Chr10 | | 0.727 | | 0.695 | | 0.233 | | 5.790 | 0.682 | C | |
| **236 Vertebrate chromosomes** | | **Accession no.** | | ***χ_r_*,*_gl_*** | | ***χ_c_*,*_gl_*** | | ***χ_i_*,*_gl_*** | | ***L* (Mb)** | ***p*** | **Type** | |
| *D. rerio* | | Chr1 | | 1.007 | | 0.933 | | 0.020 | | 58.790 | 0.637 | D | |
| *D. rerio* | | Chr2 | | 1.016 | | 0.942 | | 0.052 | | 49.510 | 0.634 | D | |
| *D. rerio* | | Chr3 | | 1.019 | | 0.945 | | 0.018 | | 42.370 | 0.637 | D | |
| *D. rerio* | | Chr4 | | 1.024 | | 0.948 | | 0.022 | | 32.130 | 0.640 | D | |
| *D. rerio* | | Chr5 | | 1.016 | | 0.941 | | 0.024 | | 64.510 | 0.637 | D | |
| *D. rerio* | | Chr6 | | 1.010 | | 0.936 | | 0.023 | | 31.520 | 0.641 | D | |
| *D. rerio* | | Chr7 | | 1.008 | | 0.934 | | 0.018 | | 59.420 | 0.636 | D | |
| *D. rerio* | | Chr8 | | 1.016 | | 0.942 | | 0.032 | | 35.440 | 0.638 | D | |
| *D. rerio* | | Chr9 | | 1.021 | | 0.946 | | 0.024 | | 41.790 | 0.638 | D | |
| *D. rerio* | | Chr10 | | 1.023 | | 0.948 | | 0.019 | | 34.880 | 0.639 | D | |
| *D. rerio* | | Chr11 | | 1.015 | | 0.940 | | 0.023 | | 35.200 | 0.637 | D | |
| *D. rerio* | | Chr12 | | 1.015 | | 0.941 | | 0.017 | | 33.160 | 0.637 | D | |
| *D. rerio* | | Chr13 | | 1.014 | | 0.939 | | 0.016 | | 35.700 | 0.636 | D | |
| *D. rerio* | | Chr14 | | 1.021 | | 0.946 | | 0.016 | | 56.970 | 0.634 | D | |
| *D. rerio* | | Chr15 | | 1.013 | | 0.939 | | 0.044 | | 34.530 | 0.635 | D | |
| *D. rerio* | | Chr16 | | 1.014 | | 0.940 | | 0.015 | | 40.610 | 0.637 | D | |
| *D. rerio* | | Chr17 | | 1.021 | | 0.946 | | 0.019 | | 40.250 | 0.638 | D | |
| *D. rerio* | | Chr18 | | 1.015 | | 0.940 | | 0.017 | | 45.570 | 0.635 | D | |
| *D. rerio* | | Chr19 | | 1.014 | | 0.939 | | 0.019 | | 48.960 | 0.635 | D | |
| *D. rerio* | | Chr20 | | 1.020 | | 0.946 | | 0.021 | | 57.250 | 0.636 | D | |
| *D. rerio* | | Chr21 | | 1.015 | | 0.940 | | 0.025 | | 38.620 | 0.635 | D | |
| *D. rerio* | | Chr22 | | 1.014 | | 0.939 | | 0.025 | | 35.070 | 0.633 | D | |
| *D. rerio* | | Chr23 | | 1.011 | | 0.937 | | 0.027 | | 40.330 | 0.637 | D | |
| *D. rerio* | | Chr24 | | 1.014 | | 0.939 | | 0.026 | | 24.300 | 0.639 | D | |
| *D. rerio* | | Chr25 | | 1.009 | | 0.935 | | 0.021 | | 24.510 | 0.635 | D | |
| *B. taurus* | | Chr01 | | 0.969 | | 0.897 | | 0.024 | | 81.970 | 0.592 | D | |
| *B. taurus* | | Chr02 | | 0.963 | | 0.891 | | 0.010 | | 69.610 | 0.583 | D | |
| *B. taurus* | | Chr03 | | 0.953 | | 0.883 | | 0.016 | | 70.040 | 0.575 | D | |
| *B. taurus* | | Chr04 | | 0.963 | | 0.891 | | 0.013 | | 56.070 | 0.587 | D | |
| *B. taurus* | | Chr05 | | 0.948 | | 0.878 | | 0.012 | | 61.950 | 0.570 | D | |
| *B. taurus* | | Chr06 | | 0.974 | | 0.901 | | 0.012 | | 55.290 | 0.596 | D | |
| *B. taurus* | | Chr07 | | 0.958 | | 0.886 | | 0.019 | | 56.570 | 0.563 | D | |
| *B. taurus* | | Chr08 | | 0.964 | | 0.892 | | 0.010 | | 48.840 | 0.581 | D | |
| *B. taurus* | | Chr09 | | 0.969 | | 0.897 | | 0.015 | | 50.580 | 0.595 | D | |
| *B. taurus* | | Chr10 | | 0.955 | | 0.884 | | 0.028 | | 57.910 | 0.580 | D | |
| *B. taurus* | | Chr11 | | 0.955 | | 0.883 | | 0.017 | | 70.930 | 0.566 | D | |
| *B. taurus* | | Chr12 | | 0.963 | | 0.891 | | 0.012 | | 39.570 | 0.584 | D | |
| *B. taurus* | | Chr13 | | 0.952 | | 0.882 | | 0.034 | | 51.470 | 0.558 | D | |
| *B. taurus* | | Chr14 | | 0.966 | | 0.894 | | 0.015 | | 40.820 | 0.580 | D | |
| *B. taurus* | | Chr15 | | 0.953 | | 0.882 | | 0.010 | | 43.130 | 0.570 | C | |
| *B. taurus* | | Chr16 | | 0.954 | | 0.884 | | 0.038 | | 47.470 | 0.568 | D | |
| *B. taurus* | | Chr17 | | 0.957 | | 0.886 | | 0.014 | | 37.550 | 0.562 | D | |
| *B. taurus* | | Chr18 | | 0.938 | | 0.867 | | 0.018 | | 46.640 | 0.539 | D | |
| *B. taurus* | | Chr19 | | 0.929 | | 0.860 | | 0.011 | | 48.020 | 0.536 | D | |
| *B. taurus* | | Chr20 | | 0.970 | | 0.898 | | 0.022 | | 34.500 | 0.587 | D | |
| *B. taurus* | | Chr21 | | 0.956 | | 0.885 | | 0.031 | | 39.300 | 0.562 | D | |
| *B. taurus* | | Chr22 | | 0.960 | | 0.888 | | 0.021 | | 39.390 | 0.566 | D | |
| *B. taurus* | | Chr23 | | 0.938 | | 0.868 | | 0.023 | | 34.250 | 0.565 | D | |
| *B. taurus* | | Chr24 | | 0.961 | | 0.889 | | 0.010 | | 36.440 | 0.579 | D | |
| *B. taurus* | | Chr25 | | 0.943 | | 0.873 | | 0.012 | | 32.870 | 0.532 | C | |
| *B. taurus* | | Chr26 | | 0.958 | | 0.887 | | 0.012 | | 28.840 | 0.569 | D | |
| *B. taurus* | | Chr27 | | 0.958 | | 0.886 | | 0.024 | | 24.950 | 0.583 | D | |
| *B. taurus* | | Chr28 | | 0.966 | | 0.894 | | 0.025 | | 28.330 | 0.575 | D | |
| *B. taurus* | | Chr29 | | 0.948 | | 0.877 | | 0.020 | | 35.860 | 0.551 | D | |
| *B. taurus* | | ChrX | | 0.974 | | 0.902 | | 0.020 | | 39.930 | 0.585 | D | |
| *G. gallus* | | Chr1 | | 1.065 | | 0.987 | | 0.020 | | 185.000 | 0.602 | D | |
| *G. gallus* | | Chr2 | | 1.072 | | 0.993 | | 0.015 | | 143.840 | 0.604 | D | |
| *G. gallus* | | Chr3 | | 1.078 | | 0.999 | | 0.028 | | 107.450 | 0.600 | D | |
| *G. gallus* | | Chr4 | | 1.085 | | 1.005 | | 0.017 | | 89.010 | 0.601 | D | |
| *G. gallus* | | Chr5 | | 1.085 | | 1.006 | | 0.037 | | 54.070 | 0.591 | D | |
| *G. gallus* | | Chr6 | | 1.093 | | 1.013 | | 0.027 | | 33.400 | 0.585 | D | |
| *G. gallus* | | Chr7 | | 1.097 | | 1.017 | | 0.013 | | 35.410 | 0.588 | D | |
| *G. gallus* | | Chr8 | | 1.094 | | 1.015 | | 0.059 | | 28.180 | 0.582 | D | |
| *G. gallus* | | Chr9 | | 1.101 | | 1.022 | | 0.068 | | 23.050 | 0.573 | D | |
| *G. gallus* | | Chr10 | | 1.093 | | 1.015 | | 0.069 | | 22.470 | 0.577 | D | |
| *G. gallus* | | Chr11 | | 1.098 | | 1.023 | | 0.116 | | 19.100 | 0.582 | D | |
| *G. gallus* | | Chr12 | | 1.107 | | 1.026 | | 0.015 | | 19.040 | 0.569 | C | |
| *G. gallus* | | Chr13 | | 1.103 | | 1.022 | | 0.017 | | 17.900 | 0.560 | C | |
| *G. gallus* | | Chr14 | | 1.098 | | 1.018 | | 0.032 | | 20.160 | 0.558 | D | |
| *G. gallus* | | Chr15 | | 1.101 | | 1.020 | | 0.020 | | 12.220 | 0.549 | D | |
| *G. gallus* | | Chr16 | | 1.026 | | 0.952 | | 0.127 | | 0.430 | 0.460 | D | |
| *G. gallus* | | Chr17 | | 1.102 | | 1.022 | | 0.059 | | 9.890 | 0.526 | D | |
| *G. gallus* | | Chr18 | | 1.105 | | 1.024 | | 0.034 | | 8.800 | 0.543 | D | |
| *G. gallus* | | Chr19 | | 1.104 | | 1.023 | | 0.034 | | 9.320 | 0.535 | C | |
| *G. gallus* | | Chr20 | | 1.106 | | 1.025 | | 0.039 | | 13.290 | 0.544 | D | |
| *G. gallus* | | Chr21 | | 1.101 | | 1.028 | | 0.147 | | 6.040 | 0.530 | D | |
| *G. gallus* | | Chr22 | | 1.100 | | 1.032 | | 0.182 | | 2.190 | 0.565 | D | |
| *G. gallus* | | Chr23 | | 1.117 | | 1.034 | | 0.030 | | 5.030 | 0.503 | D | |
| *G. gallus* | | Chr24 | | 1.115 | | 1.040 | | 0.146 | | 5.880 | 0.509 | D | |
| *G. gallus* | | Chr25 | | 1.086 | | 1.006 | | 0.069 | | 1.440 | 0.475 | D | |
| *G. gallus* | | Chr26 | | 1.117 | | 1.034 | | 0.032 | | 3.670 | 0.494 | C | |
| *G. gallus* | | Chr27 | | 1.095 | | 1.017 | | 0.099 | | 3.200 | 0.502 | D | |
| *G. gallus* | | Chr28 | | 1.080 | | 1.000 | | 0.025 | | 4.050 | 0.521 | D | |
| *G. gallus* | | ChrW | | 1.061 | | 0.983 | | 0.036 | | 4.360 | 0.609 | C | |
| *G. gallus* | | ChrZ | | 1.062 | | 0.983 | | 0.015 | | 45.170 | 0.602 | D | |
| *C. familiaris* | | Chr01 | | 0.926 | | 0.857 | | 0.020 | | 121.610 | 0.585 | D | |
| *C. familiaris* | | Chr02 | | 0.914 | | 0.845 | | 0.009 | | 84.340 | 0.572 | D | |
| *C. familiaris* | | Chr03 | | 0.938 | | 0.867 | | 0.017 | | 91.200 | 0.597 | D | |
| *C. familiaris* | | Chr04 | | 0.934 | | 0.865 | | 0.014 | | 88.140 | 0.597 | D | |
| *C. familiaris* | | Chr05 | | 0.929 | | 0.859 | | 0.028 | | 88.460 | 0.558 | D | |
| *C. familiaris* | | Chr06 | | 0.928 | | 0.859 | | 0.019 | | 76.910 | 0.574 | D | |
| *C. familiaris* | | Chr07 | | 0.923 | | 0.854 | | 0.023 | | 80.300 | 0.591 | D | |
| *C. familiaris* | | Chr08 | | 0.925 | | 0.857 | | 0.042 | | 73.880 | 0.595 | D | |
| *C. familiaris* | | Chr09 | | 0.909 | | 0.841 | | 0.018 | | 60.590 | 0.541 | D | |
| *C. familiaris* | | Chr10 | | 0.916 | | 0.847 | | 0.010 | | 68.880 | 0.573 | D | |
| *C. familiaris* | | Chr11 | | 0.924 | | 0.855 | | 0.020 | | 73.830 | 0.598 | D | |
| *C. familiaris* | | Chr12 | | 0.921 | | 0.852 | | 0.011 | | 72.220 | 0.611 | D | |
| *C. familiaris* | | Chr13 | | 0.934 | | 0.864 | | 0.022 | | 62.640 | 0.601 | D | |
| *C. familiaris* | | Chr14 | | 0.929 | | 0.860 | | 0.018 | | 60.580 | 0.613 | D | |
| *C. familiaris* | | Chr15 | | 0.912 | | 0.844 | | 0.009 | | 63.710 | 0.599 | C | |
| *C. familiaris* | | Chr16 | | 0.929 | | 0.860 | | 0.045 | | 58.760 | 0.591 | D | |
| *C. familiaris* | | Chr17 | | 0.921 | | 0.852 | | 0.015 | | 64.000 | 0.583 | D | |
| *C. familiaris* | | Chr18 | | 0.921 | | 0.852 | | 0.015 | | 55.220 | 0.573 | D | |
| *C. familiaris* | | Chr19 | | 0.935 | | 0.865 | | 0.009 | | 53.540 | 0.617 | C | |
| *C. familiaris* | | Chr20 | | 0.932 | | 0.862 | | 0.023 | | 57.660 | 0.557 | D | |
| *C. familiaris* | | Chr21 | | 0.922 | | 0.854 | | 0.046 | | 50.710 | 0.599 | D | |
| *C. familiaris* | | Chr22 | | 0.947 | | 0.877 | | 0.024 | | 61.180 | 0.621 | D | |
| *C. familiaris* | | Chr23 | | 0.934 | | 0.864 | | 0.033 | | 52.060 | 0.602 | D | |
| *C. familiaris* | | Chr24 | | 0.930 | | 0.861 | | 0.009 | | 47.360 | 0.556 | C | |
| *C. familiaris* | | Chr25 | | 0.926 | | 0.856 | | 0.012 | | 51.080 | 0.587 | D | |
| *C. familiaris* | | Chr26 | | 0.931 | | 0.861 | | 0.020 | | 38.670 | 0.546 | D | |
| *C. familiaris* | | Chr27 | | 0.906 | | 0.838 | | 0.010 | | 45.630 | 0.600 | C | |
| *C. familiaris* | | Chr28 | | 0.928 | | 0.859 | | 0.024 | | 40.890 | 0.565 | D | |
| *C. familiaris* | | Chr29 | | 0.934 | | 0.864 | | 0.013 | | 41.630 | 0.619 | D | |
| *C. familiaris* | | Chr30 | | 0.912 | | 0.844 | | 0.017 | | 39.960 | 0.586 | D | |
| *C. familiaris* | | Chr31 | | 0.936 | | 0.866 | | 0.011 | | 38.840 | 0.607 | D | |
| *C. familiaris* | | Chr32 | | 0.933 | | 0.863 | | 0.018 | | 38.650 | 0.631 | D | |
| *C. familiaris* | | Chr33 | | 0.913 | | 0.845 | | 0.030 | | 31.180 | 0.609 | D | |
| *C. familiaris* | | Chr34 | | 0.937 | | 0.867 | | 0.034 | | 41.870 | 0.597 | D | |
| *C. familiaris* | | Chr35 | | 0.922 | | 0.853 | | 0.028 | | 26.370 | 0.587 | D | |
| *C. familiaris* | | Chr36 | | 0.925 | | 0.857 | | 0.032 | | 30.680 | 0.615 | D | |
| *C. familiaris* | | Chr37 | | 0.919 | | 0.850 | | 0.033 | | 30.730 | 0.599 | D | |
| *C. familiaris* | | Chr38 | | 0.914 | | 0.845 | | 0.024 | | 23.670 | 0.592 | D | |
| *C. familiaris* | | ChrX | | 0.928 | | 0.858 | | 0.008 | | 122.250 | 0.598 | D | |
| *M. musculus* | | Chr1 | | 0.929 | | 0.858 | | 0.009 | | 193.400 | 0.589 | D | |
| *M. musculus* | | Chr2 | | 0.932 | | 0.861 | | 0.009 | | 178.440 | 0.579 | D | |
| *M. musculus* | | Chr3 | | 0.932 | | 0.861 | | 0.015 | | 157.320 | 0.596 | D | |
| *M. musculus* | | Chr4 | | 0.925 | | 0.855 | | 0.010 | | 150.930 | 0.577 | D | |
| *M. musculus* | | Chr5 | | 0.926 | | 0.855 | | 0.005 | | 148.700 | 0.575 | C | |
| *M. musculus* | | Chr6 | | 0.930 | | 0.859 | | 0.007 | | 147.850 | 0.586 | D | |
| *M. musculus* | | Chr7 | | 0.927 | | 0.857 | | 0.022 | | 137.650 | 0.569 | D | |
| *M. musculus* | | Chr8 | | 0.928 | | 0.857 | | 0.008 | | 124.990 | 0.577 | D | |
| *M. musculus* | | Chr9 | | 0.926 | | 0.856 | | 0.009 | | 120.630 | 0.573 | D | |
| *M. musculus* | | Chr10 | | 0.924 | | 0.853 | | 0.016 | | 127.650 | 0.586 | D | |
| *M. musculus* | | Chr11 | | 0.919 | | 0.849 | | 0.011 | | 118.640 | 0.562 | D | |
| *M. musculus* | | Chr12 | | 0.933 | | 0.863 | | 0.041 | | 114.570 | 0.584 | D | |
| *M. musculus* | | Chr13 | | 0.930 | | 0.859 | | 0.016 | | 113.700 | 0.584 | D | |
| *M. musculus* | | Chr14 | | 0.935 | | 0.864 | | 0.008 | | 117.260 | 0.589 | D | |
| *M. musculus* | | Chr15 | | 0.930 | | 0.859 | | 0.012 | | 101.500 | 0.581 | D | |
| *M. musculus* | | Chr16 | | 0.935 | | 0.863 | | 0.008 | | 95.610 | 0.591 | D | |
| *M. musculus* | | Chr17 | | 0.925 | | 0.854 | | 0.014 | | 89.790 | 0.574 | D | |
| *M. musculus* | | Chr18 | | 0.930 | | 0.859 | | 0.011 | | 88.420 | 0.586 | D | |
| *M. musculus* | | Chr19 | | 0.920 | | 0.850 | | 0.029 | | 58.600 | 0.572 | D | |
| *M. musculus* | | ChrX | | 0.935 | | 0.864 | | 0.010 | | 159.250 | 0.608 | D | |
| *M. musculus* | | ChrY | | 0.882 | | 0.815 | | 0.017 | | 26.990 | 0.611 | D | |
| *R. norvegicus* | | Chr1 | | 0.913 | | 0.844 | | 0.007 | | 245.870 | 0.573 | N/A | |
| *R. norvegicus* | | Chr2 | | 0.919 | | 0.849 | | 0.017 | | 239.190 | 0.596 | N/A | |
| *R. norvegicus* | | Chr3 | | 0.916 | | 0.846 | | 0.013 | | 158.690 | 0.576 | N/A | |
| *R. norvegicus* | | Chr4 | | 0.916 | | 0.846 | | 0.010 | | 174.290 | 0.585 | N/A | |
| *R. norvegicus* | | Chr5 | | 0.913 | | 0.843 | | 0.010 | | 159.370 | 0.578 | N/A | |
| *R. norvegicus* | | Chr6 | | 0.916 | | 0.846 | | 0.020 | | 136.090 | 0.582 | N/A | |
| *R. norvegicus* | | Chr7 | | 0.910 | | 0.840 | | 0.007 | | 132.640 | 0.576 | N/A | |
| *R. norvegicus* | | Chr8 | | 0.909 | | 0.840 | | 0.012 | | 118.820 | 0.571 | N/A | |
| *R. norvegicus* | | Chr9 | | 0.910 | | 0.840 | | 0.006 | | 105.690 | 0.582 | N/A | |
| *R. norvegicus* | | Chr10 | | 0.900 | | 0.831 | | 0.017 | | 101.750 | 0.551 | N/A | |
| *R. norvegicus* | | Chr11 | | 0.916 | | 0.846 | | 0.031 | | 82.920 | 0.592 | N/A | |
| *R. norvegicus* | | Chr12 | | 0.884 | | 0.815 | | 0.025 | | 41.870 | 0.530 | N/A | |
| *R. norvegicus* | | Chr13 | | 0.907 | | 0.838 | | 0.027 | | 102.800 | 0.587 | N/A | |
| *R. norvegicus* | | Chr14 | | 0.918 | | 0.847 | | 0.010 | | 102.330 | 0.587 | N/A | |
| *R. norvegicus* | | Chr15 | | 0.916 | | 0.846 | | 0.010 | | 100.270 | 0.588 | N/A | |
| *R. norvegicus* | | Chr16 | | 0.915 | | 0.845 | | 0.032 | | 82.530 | 0.582 | N/A | |
| *R. norvegicus* | | Chr17 | | 0.909 | | 0.840 | | 0.019 | | 88.180 | 0.575 | N/A | |
| *R. norvegicus* | | Chr18 | | 0.912 | | 0.842 | | 0.009 | | 80.130 | 0.584 | N/A | |
| *R. norvegicus* | | Chr19 | | 0.903 | | 0.833 | | 0.017 | | 54.220 | 0.558 | N/A | |
| *R. norvegicus* | | Chr20 | | 0.890 | | 0.821 | | 0.017 | | 49.810 | 0.565 | N/A | |
| *R. norvegicus* | | ChrX | | 0.929 | | 0.859 | | 0.012 | | 146.990 | 0.608 | N/A | |
| *M. mulatta* | | Chr01 | | 0.986 | | 0.913 | | 0.012 | | 219.580 | 0.581 | N/A | |
| *M. mulatta* | | Chr02 | | 1.002 | | 0.928 | | 0.013 | | 186.020 | 0.605 | N/A | |
| *M. mulatta* | | Chr03 | | 0.998 | | 0.924 | | 0.014 | | 180.620 | 0.594 | N/A | |
| *M. mulatta* | | Chr04 | | 0.996 | | 0.922 | | 0.016 | | 165.560 | 0.604 | N/A | |
| *M. mulatta* | | Chr05 | | 1.009 | | 0.934 | | 0.006 | | 178.810 | 0.621 | N/A | |
| *M. mulatta* | | Chr06 | | 1.004 | | 0.930 | | 0.015 | | 173.740 | 0.605 | N/A | |
| *M. mulatta* | | Chr07 | | 0.994 | | 0.920 | | 0.008 | | 162.970 | 0.586 | N/A | |
| *M. mulatta* | | Chr08 | | 1.002 | | 0.927 | | 0.008 | | 139.570 | 0.600 | N/A | |
| *M. mulatta* | | Chr09 | | 0.999 | | 0.925 | | 0.014 | | 126.870 | 0.584 | N/A | |
| *M. mulatta* | | Chr10 | | 0.987 | | 0.914 | | 0.017 | | 91.100 | 0.545 | N/A | |
| *M. mulatta* | | Chr11 | | 0.994 | | 0.920 | | 0.024 | | 133.020 | 0.591 | N/A | |
| *M. mulatta* | | Chr12 | | 1.003 | | 0.928 | | 0.015 | | 103.340 | 0.607 | N/A | |
| *M. mulatta* | | Chr13 | | 0.995 | | 0.921 | | 0.007 | | 128.790 | 0.590 | N/A | |
| *M. mulatta* | | Chr14 | | 0.991 | | 0.917 | | 0.011 | | 123.030 | 0.584 | N/A | |
| *M. mulatta* | | Chr15 | | 0.995 | | 0.921 | | 0.010 | | 106.870 | 0.585 | N/A | |
| *M. mulatta* | | Chr16 | | 0.975 | | 0.902 | | 0.012 | | 73.890 | 0.544 | N/A | |
| *M. mulatta* | | Chr17 | | 1.009 | | 0.934 | | 0.013 | | 92.050 | 0.615 | N/A | |
| *M. mulatta* | | Chr18 | | 1.002 | | 0.927 | | 0.011 | | 71.130 | 0.601 | N/A | |
| *M. mulatta* | | Chr19 | | 0.961 | | 0.889 | | 0.028 | | 52.140 | 0.518 | N/A | |
| *M. mulatta* | | Chr20 | | 0.993 | | 0.920 | | 0.046 | | 71.750 | 0.553 | N/A | |
| *M. mulatta* | | ChrX | | 1.009 | | 0.934 | | 0.017 | | 144.360 | 0.607 | N/A | |
| *P. troglodytes* | | Chr01 | | 0.999 | | 0.925 | | 0.008 | | 225.770 | 0.584 | N/A | |
| *P. troglodytes* | | Chr02A | | 1.006 | | 0.932 | | 0.008 | | 108.680 | 0.591 | N/A | |
| *P. troglodytes* | | Chr02B | | 1.014 | | 0.939 | | 0.016 | | 129.830 | 0.605 | N/A | |
| *P. troglodytes* | | Chr03 | | 1.012 | | 0.937 | | 0.007 | | 198.160 | 0.604 | N/A | |
| *P. troglodytes* | | Chr04 | | 1.022 | | 0.946 | | 0.012 | | 192.990 | 0.618 | N/A | |
| *P. troglodytes* | | Chr05 | | 1.015 | | 0.940 | | 0.010 | | 178.080 | 0.606 | N/A | |
| *P. troglodytes* | | Chr06 | | 1.009 | | 0.934 | | 0.009 | | 173.500 | 0.605 | N/A | |
| *P. troglodytes* | | Chr07 | | 1.010 | | 0.935 | | 0.007 | | 157.680 | 0.594 | N/A | |
| *P. troglodytes* | | Chr08 | | 1.013 | | 0.938 | | 0.009 | | 145.040 | 0.600 | N/A | |
| *P. troglodytes* | | Chr09 | | 1.006 | | 0.931 | | 0.010 | | 116.340 | 0.588 | N/A | |
| *P. troglodytes* | | Chr10 | | 1.010 | | 0.934 | | 0.009 | | 133.150 | 0.586 | N/A | |
| *P. troglodytes* | | Chr11 | | 1.003 | | 0.929 | | 0.008 | | 131.560 | 0.586 | N/A | |
| *P. troglodytes* | | Chr12 | | 1.006 | | 0.931 | | 0.015 | | 131.900 | 0.593 | N/A | |
| *P. troglodytes* | | Chr13 | | 1.021 | | 0.945 | | 0.013 | | 96.720 | 0.615 | N/A | |
| *P. troglodytes* | | Chr14 | | 1.006 | | 0.932 | | 0.031 | | 88.170 | 0.592 | N/A | |
| *P. troglodytes* | | Chr15 | | 1.002 | | 0.928 | | 0.010 | | 79.840 | 0.579 | N/A | |
| *P. troglodytes* | | Chr16 | | 1.007 | | 0.932 | | 0.022 | | 80.260 | 0.555 | N/A | |
| *P. troglodytes* | | Chr17 | | 0.987 | | 0.914 | | 0.027 | | 78.170 | 0.547 | N/A | |
| *P. troglodytes* | | Chr18 | | 1.015 | | 0.940 | | 0.012 | | 75.840 | 0.603 | N/A | |
| *P. troglodytes* | | Chr19 | | 0.976 | | 0.903 | | 0.019 | | 54.070 | 0.521 | N/A | |
| *P. troglodytes* | | Chr20 | | 1.005 | | 0.931 | | 0.053 | | 59.730 | 0.561 | N/A | |
| *P. troglodytes* | | Chr21 | | 1.008 | | 0.933 | | 0.023 | | 32.710 | 0.590 | N/A | |
| *P. troglodytes* | | Chr22 | | 0.993 | | 0.918 | | 0.016 | | 33.420 | 0.521 | N/A | |
| *P. troglodytes* | | ChrX | | 1.018 | | 0.942 | | 0.008 | | 133.730 | 0.608 | N/A | |
| *P. troglodytes* | | ChrY | | 1.019 | | 0.944 | | 0.057 | | 23.460 | 0.599 | N/A | |
| *H. sapiens* | | Chr01 | | 0.998 | | 0.924 | | 0.008 | | 217.470 | 0.583 | D | |
| *H. sapiens* | | Chr02 | | 1.009 | | 0.934 | | 0.009 | | 232.340 | 0.598 | D | |
| *H. sapiens* | | Chr03 | | 1.011 | | 0.936 | | 0.007 | | 194.030 | 0.604 | D | |
| *H. sapiens* | | Chr04 | | 1.020 | | 0.944 | | 0.006 | | 185.870 | 0.618 | D | |
| *H. sapiens* | | Chr05 | | 1.014 | | 0.939 | | 0.012 | | 175.070 | 0.605 | D | |
| *H. sapiens* | | Chr06 | | 1.005 | | 0.930 | | 0.011 | | 178.820 | 0.602 | D | |
| *H. sapiens* | | Chr07 | | 1.008 | | 0.933 | | 0.008 | | 305.370 | 0.593 | D | |
| *H. sapiens* | | Chr08 | | 1.012 | | 0.937 | | 0.008 | | 140.200 | 0.599 | D | |
| *H. sapiens* | | Chr09 | | 1.005 | | 0.930 | | 0.007 | | 108.480 | 0.585 | D | |
| *H. sapiens* | | Chr10 | | 1.009 | | 0.934 | | 0.009 | | 127.150 | 0.584 | D | |
| *H. sapiens* | | Chr11 | | 1.002 | | 0.927 | | 0.010 | | 129.770 | 0.585 | D | |
| *H. sapiens* | | Chr12 | | 1.004 | | 0.929 | | 0.006 | | 131.380 | 0.592 | D | |
| *H. sapiens* | | Chr13 | | 1.020 | | 0.944 | | 0.014 | | 95.230 | 0.615 | D | |
| *H. sapiens* | | Chr14 | | 1.005 | | 0.931 | | 0.032 | | 86.940 | 0.591 | D | |
| *H. sapiens* | | Chr15 | | 1.001 | | 0.927 | | 0.013 | | 76.380 | 0.579 | D | |
| *H. sapiens* | | Chr16 | | 1.003 | | 0.928 | | 0.018 | | 71.290 | 0.552 | D | |
| *H. sapiens* | | Chr17 | | 0.986 | | 0.913 | | 0.011 | | 74.750 | 0.545 | D | |
| *H. sapiens* | | Chr18 | | 1.014 | | 0.938 | | 0.011 | | 73.370 | 0.602 | D | |
| *H. sapiens* | | Chr19 | | 0.971 | | 0.899 | | 0.021 | | 54.970 | 0.516 | D | |
| *H. sapiens* | | Chr20 | | 1.002 | | 0.928 | | 0.045 | | 59.230 | 0.559 | D | |
| *H. sapiens* | | Chr21 | | 1.007 | | 0.932 | | 0.031 | | 32.990 | 0.591 | D | |
| *H. sapiens* | | Chr22 | | 0.994 | | 0.920 | | 0.021 | | 33.530 | 0.521 | D | |
| *H. sapiens* | | ChrX | | 1.016 | | 0.941 | | 0.012 | | 151.310 | 0.606 | D | |
| *H. sapiens* | | ChrY | | 1.021 | | 0.945 | | 0.069 | | 9.560 | 0.604 | D | |
