# Supplementary material for: Inverse Symmetry in Complete Genomes and Whole-Genome Inverse Duplication
Source: PLoS One. 2009 Nov 9;4(11):e7553. doi: 10.1371/journal.pone.0007553 (PMC2771390; doi:10.1371/journal.pone.0007553)
Supplement: Table S2 — Classification of chromosomes by inverse symmetry type and χi,bg and rχ values. (0.83 MB DOC) [file pone.0007553.s002.doc]

Table S2. Classification of chromosomes by inverse symmetry type and ***χ_i_*,*_bg_*** and ***r_χ_*** values

| **Type A – Eubacteria (119 chromosomes)** | **Accession no.** | ***χ_i_*,*_bg_*** | ***r_χ_*** |
| --- | --- | --- | --- |
| *Anaplasma marginale str. St. Maries* | NC_004842 | 0.566 | 4.187 |
| *Anaplasma phagocytophilum HZ* | NC_007797 | 0.718 | 2.463 |
| *Bacillus anthracis str. Ames* | NC_003997 | 0.989 | 10.432 |
| *Bacillus anthracis str. 'Ames Ancestor'* | NC_007530 | 0.989 | 10.425 |
| *Bacillus anthracis str. Sterne* | NC_005945 | 0.989 | 10.284 |
| *Bacillus cereus ATCC 10987* | NC_003909 | 1.024 | 17.955 |
| *Bacillus cereus ATCC 14579* | NC_004722 | 1.034 | 24.395 |
| *Bacillus cereus E33L* | NC_006274 | 1.003 | 12.393 |
| *Bacillus clausii KSM-K16* | NC_006582 | 0.525 | 12.481 |
| *Bacillus halodurans C-125* | NC_002570 | 0.807 | 5.558 |
| *Bacillus licheniformis ATCC 14580* | NC_006270 | 0.433 | 18.389 |
| *Bacillus licheniformis ATCC 14580* | NC_006322 | 0.434 | 17.873 |
| *Bacillus subtilis subsp. subtilis str. 168* | NC_000964 | 0.534 | 12.928 |
| *Bacillus thuringiensis serovar konkukian str. 97-27* | NC_005957 | 1.009 | 11.677 |
| *Bacteroides fragilis NCTC 9343* | NC_003228 | 0.649 | 11.553 |
| *Bacteroides fragilis YCH46* | NC_006347 | 0.648 | 13.822 |
| *Bacteroides thetaiotaomicron VPI-5482* | NC_004663 | 0.586 | 6.904 |
| *Bartonella henselae str. Houston-1* | NC_005956 | 0.712 | 3.370 |
| *Bartonella quintana str. Toulouse* | NC_005955 | 0.743 | 4.932 |
| *Bdellovibrio bacteriovorus HD100* | NC_005363 | 0.414 | 9.566 |
| *Borrelia burgdorferi B31* | NC_001318 | 0.704 | 10.209 |
| *Borrelia garinii PBi chromosome linear* | NC_006156 | 0.704 | 8.858 |
| *Buchnera aphidicola str. Bp (Baizongia pistaciae)* | NC_004545 | 0.970 | 4.762 |
| *Campylobacter jejuni RM1221* | NC_003912 | 0.404 | 8.472 |
| *Campylobacter jejuni subsp. jejuni NCTC 11168* | NC_002163 | 0.430 | 7.799 |
| *Candidatus Blochmannia floridanus* | NC_005061 | 1.070 | 3.095 |
| *Candidatus Blochmannia pennsylvanicus str. BPEN* | NC_007292 | 0.854 | 2.942 |
| *Carboxydothermus hydrogenoformans Z-2901* | NC_007503 | 0.744 | 24.145 |
| *Chlamydia muridarum Nigg* | NC_002620 | 0.689 | 9.946 |
| *Chlamydia trachomatis A/HAR-13* | NC_007429 | 0.651 | 10.810 |
| *Chlamydia trachomatis D/UW-3/CX* | NC_000117 | 0.652 | 11.034 |
| *Chlamydophila abortus S26/3* | NC_004552 | 0.650 | 7.598 |
| *Chlamydophila caviae GPIC* | NC_003361 | 0.568 | 10.768 |
| *Chlamydophila felis Fe/C-56* | NC_007899 | 0.600 | 9.096 |
| *Chlamydophila pneumoniae AR39* | NC_002179 | 0.535 | 7.201 |
| *Clostridium acetobutylicum ATCC 824* | NC_003030 | 0.986 | 19.870 |
| *Clostridium perfringens str. 13* | NC_003366 | 0.985 | 3.803 |
| *Clostridium tetani E88* | NC_004557 | 0.961 | 6.351 |
| *Corynebacterium diphtheriae NCTC 13129* | NC_002935 | 0.394 | 8.938 |
| *Coxiella burnetii RSA 493* | NC_002971 | 0.386 | 9.478 |
| *Desulfovibrio vulgaris subsp. vulgaris str. Hildenborough* | NC_002937 | 0.446 | 11.946 |
| *Ehrlichia canis str. Jake* | NC_007354 | 1.089 | 7.887 |
| *Ehrlichia chaffeensis str. Arkansas* | NC_007799 | 1.080 | 4.131 |
| *Ehrlichia ruminantium str. Gardel* | NC_006831 | 1.071 | 5.807 |
| *Ehrlichia ruminantium str. Welgevonden* | NC_005295 | 1.057 | 5.435 |
| *Ehrlichia ruminantium str. Welgevonden* | NC_006832 | 1.071 | 5.546 |
| *Enterococcus faecalis V583* | NC_004668 | 0.822 | 10.050 |
| *Geobacillus kaustophilus HTA426* | NC_006510 | 0.488 | 11.356 |
| *Hahella chejuensis KCTC 2396* | NC_007645 | 0.431 | 9.217 |
| *Lactobacillus acidophilus NCFM* | NC_006814 | 0.784 | 4.690 |
| *Lactobacillus johnsonii NCC 533* | NC_005362 | 0.799 | 5.040 |
| *Lactobacillus plantarum WCFS1* | NC_004567 | 0.717 | 13.288 |
| *Lactobacillus sakei subsp. sakei 23K* | NC_007576 | 0.697 | 11.041 |
| *Lactobacillus salivarius subsp. salivarius UCC118* | NC_007929 | 0.989 | 8.733 |
| *Lactococcus lactis subsp. lactis Il1403* | NC_002662 | 0.695 | 8.265 |
| *Lawsonia intracellularis PHE/MN1-00* | NC_008011 | 0.619 | 6.183 |
| *Legionella pneumophila str. Paris* | NC_006368 | 0.394 | 7.747 |
| *Legionella pneumophila subsp. pneumophila str. Philadelphia 1* | NC_002942 | 0.402 | 10.635 |
| *Leptospira interrogans serovar Copenhageni str. Fiocruz L1-130* | NC_005823 | 0.358 | 10.635 |
| *Listeria innocua Clip11262* | NC_003212 | 0.791 | 7.791 |
| *Listeria monocytogenes EGD-e* | NC_003210 | 0.770 | 8.221 |
| *Listeria monocytogenes str. 4b F2365* | NC_002973 | 0.774 | 8.419 |
| *Mesoplasma florum L1* | NC_006055 | 0.844 | 9.575 |
| *Mycobacterium leprae TN* | NC_002677 | 0.592 | 6.964 |
| *Mycoplasma gallisepticum R* | NC_004829 | 0.540 | 4.873 |
| *Mycoplasma penetrans HF-2* | NC_004432 | 0.597 | 5.838 |
| *Neorickettsia sennetsu str. Miyayama* | NC_007798 | 0.814 | 6.538 |
| *Nitrosococcus oceani ATCC 19707* | NC_007484 | 0.519 | 7.089 |
| *Oceanobacillus iheyensis HTE831* | NC_004193 | 0.916 | 15.632 |
| *Pelobacter carbinolicus DSM 2380* | NC_007498 | 0.545 | 9.167 |
| *Photobacterium profundum SS9 chromosome 1* | NC_006370 | 0.459 | 8.325 |
| *Prochlorococcus marinus subsp. marinus str. CCMP1375* | NC_005042 | 0.376 | 8.760 |
| *Propionibacterium acnes KPA171202* | NC_006085 | 0.524 | 9.892 |
| *Rhodospirillum rubrum ATCC 11170* | NC_007643 | 0.174 | 11.642 |
| *Rickettsia prowazekii str. Madrid E* | NC_000963 | 0.570 | 3.750 |
| *Saccharophagus degradans 2-40* | NC_007912 | 0.469 | 18.092 |
| *Shewanella oneidensis MR-1* | NC_004347 | 0.388 | 10.729 |
| *Staphylococcus aureus RF122* | NC_007622 | 0.875 | 13.955 |
| *Staphylococcus aureus subsp. aureus COL* | NC_002951 | 0.896 | 9.936 |
| *Staphylococcus aureus subsp. aureus MRSA252* | NC_002952 | 0.873 | 8.684 |
| *Staphylococcus aureus subsp. aureus MSSA476* | NC_002953 | 0.907 | 10.094 |
| *Staphylococcus aureus subsp. aureus Mu50* | NC_002758 | 0.876 | 11.035 |
| *Staphylococcus aureus subsp. aureus MW2* | NC_003923 | 0.897 | 9.357 |
| *Staphylococcus aureus subsp. aureus N315* | NC_002745 | 0.873 | 9.705 |
| *Staphylococcus aureus subsp. aureus NCTC 8325* | NC_007795 | 0.872 | 6.504 |
| *Staphylococcus aureus subsp. aureus USA300* | NC_007793 | 0.898 | 9.024 |
| *Staphylococcus epidermidis ATCC 12228* | NC_004461 | 0.923 | 3.858 |
| *Staphylococcus epidermidis RP62A* | NC_002976 | 0.885 | 2.939 |
| *Staphylococcus haemolyticus JCSC1435* | NC_007168 | 0.876 | 5.847 |
| *Staphylococcus saprophyticus subsp. saprophyticus ATCC 15305* | NC_007350 | 0.865 | 9.164 |
| *Streptococcus agalactiae 2603V/R* | NC_004116 | 0.826 | 9.421 |
| *Streptococcus agalactiae A909* | NC_007432 | 0.809 | 5.848 |
| *Streptococcus agalactiae NEM316* | NC_004368 | 0.829 | 11.606 |
| *Streptococcus mutans UA159* | NC_004350 | 0.611 | 7.390 |
| *Streptococcus pneumoniae R6* | NC_003098 | 0.649 | 10.886 |
| *Streptococcus pneumoniae TIGR4* | NC_003028 | 0.644 | 6.129 |
| *Streptococcus pyogenes M1 GAS* | NC_002737 | 0.624 | 4.861 |
| *Streptococcus pyogenes MGAS10270* | NC_008022 | 0.666 | 8.774 |
| *Streptococcus pyogenes MGAS10394* | NC_006086 | 0.663 | 7.844 |
| *Streptococcus pyogenes MGAS10750* | NC_008024 | 0.593 | 3.730 |
| *Streptococcus pyogenes MGAS2096* | NC_008023 | 0.662 | 9.196 |
| *Streptococcus pyogenes MGAS315* | NC_004070 | 0.691 | 6.363 |
| *Streptococcus pyogenes MGAS5005* | NC_007297 | 0.652 | 7.954 |
| *Streptococcus pyogenes MGAS6180* | NC_007296 | 0.671 | 6.153 |
| *Streptococcus pyogenes MGAS8232* | NC_003485 | 0.660 | 8.898 |
| *Streptococcus pyogenes MGAS9429* | NC_008021 | 0.638 | 5.074 |
| *Streptococcus pyogenes SSI-1* | NC_004606 | 0.655 | 4.502 |
| *Streptococcus thermophilus CNRZ1066* | NC_006449 | 0.712 | 7.425 |
| *Streptococcus thermophilus LMG 18311* | NC_006448 | 0.722 | 7.511 |
| *Symbiobacterium thermophilum IAM 14863* | NC_006177 | 0.530 | 5.841 |
| *Synechococcus sp. CC9902* | NC_007513 | 0.486 | 9.725 |
| *Thermoanaerobacter tengcongensis MB4* | NC_003869 | 0.869 | 24.689 |
| *Thiomicrospira crunogena XCL-2* | NC_007520 | 0.633 | 4.804 |
| *Treponema pallidum subsp. pallidum str. Nichols* | NC_000919 | 0.723 | 7.033 |
| *Vibrio fischeri ES114 chromosome I* | NC_006840 | 0.518 | 10.040 |
| *Vibrio fischeri ES114 chromosome II* | NC_006841 | 0.564 | 3.779 |
| *Vibrio vulnificus YJ016 chromosome I* | NC_005139 | 0.396 | 7.751 |
| *Xylella fastidiosa 9a5c* | NC_002488 | 0.759 | 1.474 |
| *Xylella fastidiosa Temecula1* | NC_004556 | 0.834 | 6.641 |
| **Type B – Eubacteria (61 chromosomes)** | **Accession no.** | ***χ_i_*,*_bg_*** | ***r_χ_*** |
| *Acinetobacter sp. ADP1* | NC_005966 | 0.292 | 5.772 |
| *Buchnera aphidicola str. APS* | NC_002528 | 0.478 | 2.616 |
| *Buchnera aphidicola str. Sg* | NC_004061 | 0.508 | 2.449 |
| *Candidatus Protochlamydia amoebophila UWE25* | NC_005861 | 0.303 | 6.005 |
| *Chlamydophila pneumoniae CWL029* | NC_000922 | 0.430 | 5.604 |
| *Chlamydophila pneumoniae J138* | NC_002491 | 0.431 | 5.460 |
| *Chlamydophila pneumoniae TW-183* | NC_005043 | 0.430 | 5.504 |
| *Chromobacterium violaceum ATCC 12472* | NC_005085 | 0.243 | 5.928 |
| *Colwellia psychrerythraea 34H* | NC_003910 | 0.284 | 8.075 |
| *Corynebacterium jeikeium K411* | NC_007164 | 0.389 | 2.593 |
| *Dechloromonas aromatica RCB* | NC_007298 | 0.292 | 6.527 |
| *Dehalococcoides sp. CBDB1* | NC_007356 | 0.349 | 6.180 |
| *Desulfitobacterium hafniense Y51* | NC_007907 | 0.505 | 1.634 |
| *Desulfotalea psychrophila LSv54* | NC_006138 | 0.543 | 4.103 |
| *Desulfovibrio desulfuricans G20* | NC_007519 | 0.334 | 6.278 |
| *Erwinia carotovora subsp. atroseptica SCRI1043* | NC_004547 | 0.368 | 7.773 |
| *Escherichia coli O157:H7 EDL933* | NC_002655 | 0.194 | 7.537 |
| *Francisella tularensis subsp. holarctica* | NC_007880 | 0.483 | 3.303 |
| *Francisella tularensis subsp. tularensis Schu 4* | NC_006570 | 0.479 | 3.200 |
| *Fusobacterium nucleatum subsp. nucleatum ATCC 25586* | NC_003454 | 0.426 | 2.064 |
| *Geobacter sulfurreducens PCA* | NC_002939 | 0.292 | 4.815 |
| *Haemophilus ducreyi 35000HP* | NC_002940 | 0.414 | 1.632 |
| *Lactobacillus delbrueckii subsp. bulgaricus ATCC 11842* | NC_008054 | 0.432 | 6.536 |
| *Legionella pneumophila str. Lens* | NC_006369 | 0.405 | 5.185 |
| *Leptospira interrogans serovar Copenhageni* | NC_005824 | 0.374 | 2.927 |
| *Leptospira interrogans serovar Lai str. 56601* | NC_004342 | 0.348 | 6.032 |
| *Leptospira interrogans serovar Lai str. 56601* | NC_004343 | 0.371 | 2.777 |
| *Methylobacillus flagellatus KT* | NC_007947 | 0.331 | 7.789 |
| *Methylococcus capsulatus str. Bath* | NC_002977 | 0.276 | 6.069 |
| *Moorella thermoacetica ATCC 39073* | NC_007644 | 0.424 | 6.323 |
| *Mycoplasma capricolum subsp. capricolum ATCC 27343* | NC_007633 | 0.384 | 3.115 |
| *Myxococcus xanthus DK 1622* | NC_008095 | 0.317 | 6.532 |
| *Pelodictyon luteolum DSM 273* | NC_007512 | 0.417 | 4.100 |
| *Photobacterium profundum SS9 chromosome 2* | NC_006371 | 0.398 | 5.632 |
| *Photorhabdus luminescens subsp. laumondii TTO1* | NC_005126 | 0.546 | 4.050 |
| *Prochlorococcus marinus str. MIT 9313* | NC_005071 | 0.474 | 1.390 |
| *Prochlorococcus marinus str. NATL2A* | NC_007335 | 0.320 | 7.062 |
| *Pseudoalteromonas atlantica T6c* | NC_008228 | 0.227 | 7.794 |
| *Pseudoalteromonas haloplanktis TAC125 chromosome I* | NC_007481 | 0.237 | 6.800 |
| *Rickettsia conorii str. Malish 7* | NC_003103 | 0.409 | 3.708 |
| *Rickettsia typhi str. Wilmington* | NC_006142 | 0.537 | 2.875 |
| *Salinibacter ruber DSM 13855* | NC_007677 | 0.238 | 6.530 |
| *Salmonella enterica subsp. enterica serovar Choleraesuis* | NC_006905 | 0.237 | 5.718 |
| *Salmonella enterica subsp. enterica serovar Paratyphi A* | NC_006511 | 0.253 | 5.695 |
| *Salmonella enterica subsp. enterica serovar Typhi* | NC_003198 | 0.240 | 8.350 |
| *Shewanella denitrificans OS217* | NC_007954 | 0.298 | 6.565 |
| *Synechococcus sp. CC9605* | NC_007516 | 0.302 | 7.712 |
| *Synechococcus sp. WH 8102* | NC_005070 | 0.292 | 5.922 |
| *Tropheryma whipplei str. Twist* | NC_004572 | 0.505 | 2.698 |
| *Tropheryma whipplei TW08/27* | NC_004551 | 0.480 | 2.866 |
| *Ureaplasma parvum serovar 3 str. ATCC 700970* | NC_002162 | 0.391 | 2.267 |
| *Vibrio cholerae O1 biovar eltor str. N16961 chromosome I* | NC_002505 | 0.316 | 4.325 |
| *Vibrio parahaemolyticus RIMD 2210633 chromosome I* | NC_004603 | 0.392 | 6.377 |
| *Vibrio parahaemolyticus RIMD 2210633 chromosome II* | NC_004605 | 0.333 | 6.163 |
| *Vibrio vulnificus CMCP6* | NC_004460 | 0.262 | 7.883 |
| *Vibrio vulnificus CMCP6 chromosome I* | NC_004459 | 0.384 | 5.127 |
| *Vibrio vulnificus YJ016 chromosome II* | NC_005140 | 0.295 | 5.410 |
| *Wolinella succinogenes DSM 1740* | NC_005090 | 0.365 | 4.846 |
| *Yersinia pestis Antiqua* | NC_008150 | 0.224 | 7.896 |
| *Yersinia pestis Nepal516* | NC_008149 | 0.336 | 4.029 |
| *Yersinia pseudotuberculosis IP 32953* | NC_006155 | 0.330 | 5.225 |
| **Type B – Eukaryotes (13 chromosomes)** | **Accession no.** | ***χ_i_*,*_bg_*** | ***r_χ_*** |
| *E. cuniculi* | NC_003229 | 0.355 | 4.091 |
| *E. cuniculi* | NC_003242 | 0.435 | 3.272 |
| *S. cerevisiae* | NC_001133 | 0.529 | 2.048 |
| *S. cerevisiae* | NC_001135 | 0.426 | 1.218 |
| *S. cerevisiae* | NC_001138 | 0.430 | 2.373 |
| *P. falciparum* | NC_000521 | 0.413 | 2.613 |
| *P. falciparum* | NC_000910 | 0.527 | 4.412 |
| *P. falciparum* | NC_004314 | 0.459 | 1.626 |
| *P. falciparum* | NC_004318 | 0.461 | 1.242 |
| *P. falciparum* | NC_004325 | 0.561 | 1.818 |
| *P. falciparum* | NC_004326 | 0.405 | 1.654 |
| *P. falciparum* | NC_004327 | 0.414 | 1.914 |
| *P. falciparum* | NC_004328 | 0.468 | 1.763 |
| **Type C – Archaea (14 chromosomes)** | **Accession no.** | ***χ_i_*,*_bg_*** | ***r_χ_*** |
| *Haloarcula marismortui ATCC 43049 chromosome II* | NC_006397 | 0.239 | 1.181 |
| *Halobacterium sp. NRC-1* | NC_002607 | 0.165 | 4.087 |
| *Methanococcoides burtonii DSM 6242* | NC_007955 | 0.291 | 2.689 |
| *Methanococcus maripaludis S2* | NC_005791 | 0.215 | 2.435 |
| *Methanosarcina mazei Go1* | NC_003901 | 0.128 | 3.458 |
| *Methanosphaera stadtmanae DSM 3091* | NC_007681 | 0.365 | 2.337 |
| *Nanoarchaeum equitans Kin4-M* | NC_005213 | 0.184 | 3.631 |
| *Pyrococcus abyssi GE5* | NC_000868 | 0.172 | 3.479 |
| *Pyrococcus furiosus DSM 3638* | NC_003413 | 0.143 | 5.217 |
| *Pyrococcus horikoshii OT3* | NC_000961 | 0.209 | 2.447 |
| *Sulfolobus acidocaldarius DSM 639* | NC_007181 | 0.235 | 2.668 |
| *Sulfolobus solfataricus P2* | NC_002754 | 0.208 | 1.347 |
| *Thermoplasma acidophilum DSM 1728* | NC_002578 | 0.193 | 2.316 |
| *Thermoplasma volcanium GSS1* | NC_002689 | 0.184 | 1.968 |
| **Type C – Eubacteria (135 chromosomes)** | **Accession no.** | ***χ_i_*,*_bg_*** | ***r_χ_*** |
| *Agrobacterium tumefaciens str. C58 chromosome linear* | NC_003063 | 0.118 | 3.158 |
| *Agrobacterium tumefaciens str. C58 chromosome linear* | NC_003305 | 0.125 | 3.412 |
| *Aster yellows witches'-broom phytoplasma AYWB* | NC_007716 | 0.291 | 1.776 |
| *Azoarcus sp. EbN1* | NC_006513 | 0.106 | 3.926 |
| *Baumannia cicadellinicola str. Hc (Homalodisca coagulata)* | NC_007984 | 0.253 | 2.622 |
| *Bifidobacterium longum NCC2705* | NC_004307 | 0.197 | 4.200 |
| *Bordetella bronchiseptica RB50* | NC_002927 | 0.174 | 3.339 |
| *Bordetella parapertussis 12822* | NC_002928 | 0.182 | 2.309 |
| *Bradyrhizobium japonicum USDA 110* | NC_004463 | 0.063 | 4.726 |
| *Brucella abortus biovar 1 str. 9-941 chromosome I* | NC_006932 | 0.190 | 3.874 |
| *Brucella abortus biovar 1 str. 9-941 chromosome II* | NC_006933 | 0.198 | 2.673 |
| *Brucella melitensis 16M chromosome I* | NC_003317 | 0.203 | 4.154 |
| *Brucella melitensis 16M chromosome II* | NC_003318 | 0.194 | 4.033 |
| *Brucella melitensis biovar Abortus 2308 chromosome I* | NC_007618 | 0.190 | 3.897 |
| *Brucella melitensis biovar Abortus 2308 chromosome II* | NC_007624 | 0.197 | 2.639 |
| *Brucella suis 1330 chromosome I* | NC_004310 | 0.192 | 3.950 |
| *Brucella suis 1330 chromosome II* | NC_004311 | 0.195 | 4.202 |
| *Burkholderia cenocepacia AU 1054 chromosome 1* | NC_008060 | 0.124 | 2.992 |
| *Burkholderia cenocepacia AU 1054 chromosome 2* | NC_008061 | 0.117 | 2.650 |
| *Burkholderia mallei ATCC 23344* | NC_006349 | 0.124 | 2.984 |
| *Burkholderia pseudomallei 1710b chromosome I* | NC_007434 | 0.107 | 2.637 |
| *Burkholderia pseudomallei 1710b chromosome II* | NC_007435 | 0.122 | 2.505 |
| *Burkholderia pseudomallei K96243 chromosome 2* | NC_006351 | 0.124 | 2.837 |
| *Burkholderia sp. 383 chromosome 1* | NC_007510 | 0.125 | 2.962 |
| *Burkholderia sp. 383 chromosome 2* | NC_007511 | 0.133 | 4.798 |
| *Burkholderia sp. 383 chromosome 3* | NC_007509 | 0.133 | 5.184 |
| *Burkholderia thailandensis E264 chromosome II* | NC_007650 | 0.126 | 3.097 |
| *Burkholderia xenovorans LB400 chromosome 1* | NC_007951 | 0.114 | 3.218 |
| *Burkholderia xenovorans LB400 chromosome 2* | NC_007952 | 0.105 | 4.247 |
| *Burkholderia xenovorans LB400 chromosome 3* | NC_007953 | 0.156 | 2.710 |
| *Candidatus Pelagibacter ubique HTCC1062* | NC_007205 | 0.318 | 4.210 |
| *Chlorobium chlorochromatii CaD3* | NC_007514 | 0.226 | 3.268 |
| *Chlorobium tepidum TLS* | NC_002932 | 0.291 | 3.744 |
| *Chromohalobacter salexigens DSM 3043* | NC_007963 | 0.269 | 3.896 |
| *Corynebacterium glutamicum ATCC 13032* | NC_003450 | 0.281 | 3.806 |
| *Corynebacterium glutamicum ATCC 13032* | NC_006958 | 0.277 | 4.492 |
| *Dehalococcoides ethenogenes 195* | NC_002936 | 0.301 | 2.994 |
| *Deinococcus geothermalis DSM 11300* | NC_008025 | 0.175 | 2.389 |
| *Deinococcus radiodurans R1 chromosome 2* | NC_001264 | 0.313 | 1.288 |
| *Escherichia coli CFT073* | NC_004431 | 0.162 | 4.298 |
| *Escherichia coli K12* | NC_000913 | 0.141 | 4.040 |
| *Escherichia coli O157:H7 str. Sakai* | NC_002695 | 0.199 | 5.791 |
| *Escherichia coli UTI89* | NC_007946 | 0.160 | 3.134 |
| *Escherichia coli W3110* | AC_000091 | 0.139 | 4.753 |
| *Frankia sp. CcI3* | NC_007777 | 0.198 | 5.228 |
| *Geobacter metallireducens GS-15* | NC_007517 | 0.213 | 2.749 |
| *Gluconobacter oxydans 621H* | NC_006677 | 0.356 | 2.557 |
| *Haemophilus influenzae 86-028NP* | NC_007146 | 0.187 | 5.479 |
| *Haemophilus influenzae Rd KW20* | NC_000907 | 0.163 | 2.644 |
| *Helicobacter acinonychis str. Sheeba* | NC_008229 | 0.184 | 2.376 |
| *Helicobacter hepaticus ATCC 51449* | NC_004917 | 0.293 | 2.697 |
| *Helicobacter pylori 26695* | NC_000915 | 0.168 | 1.820 |
| *Helicobacter pylori HPAG1* | NC_008086 | 0.154 | 3.187 |
| *Helicobacter pylori J99* | NC_000921 | 0.149 | 2.252 |
| *Idiomarina loihiensis L2TR* | NC_006512 | 0.381 | 1.360 |
| *Leifsonia xyli subsp. xyli str. CTCB07* | NC_006087 | 0.144 | 2.553 |
| *Magnetospirillum magneticum AMB-1* | NC_007626 | 0.184 | 1.891 |
| *Mannheimia succiniciproducens MBEL55E* | NC_006300 | 0.297 | 2.508 |
| *Mycobacterium bovis AF2122/97* | NC_002945 | 0.192 | 3.675 |
| *Mycobacterium sp. MCS* | NC_008146 | 0.076 | 3.515 |
| *Mycobacterium tuberculosis CDC1551* | NC_002755 | 0.192 | 3.775 |
| *Mycobacterium tuberculosis H37Rv* | NC_000962 | 0.191 | 3.911 |
| *Mycoplasma genitalium G37* | NC_000908 | 0.365 | 2.224 |
| *Mycoplasma hyopneumoniae 232* | NC_006360 | 0.203 | 1.464 |
| *Mycoplasma hyopneumoniae 7448* | NC_007332 | 0.213 | 1.542 |
| *Mycoplasma hyopneumoniae J* | NC_007295 | 0.202 | 1.634 |
| *Mycoplasma mobile 163K* | NC_006908 | 0.262 | 3.244 |
| *Mycoplasma mycoides subsp. mycoides SC str. PG1* | NC_005364 | 0.346 | 2.562 |
| *Mycoplasma pneumoniae M129* | NC_000912 | 0.335 | 1.789 |
| *Mycoplasma pulmonis UAB CTIP* | NC_002771 | 0.210 | 1.959 |
| *Mycoplasma synoviae 53* | NC_007294 | 0.234 | 0.963 |
| *Neisseria gonorrhoeae FA 1090* | NC_002946 | 0.242 | 4.981 |
| *Neisseria meningitidis MC58* | NC_003112 | 0.258 | 3.312 |
| *Neisseria meningitidis Z2491* | NC_003116 | 0.249 | 4.517 |
| *Nitrobacter hamburgensis X14* | NC_007964 | 0.090 | 4.382 |
| *Nitrobacter winogradskyi Nb-255* | NC_007406 | 0.120 | 3.360 |
| *Nitrosomonas europaea ATCC 19718* | NC_004757 | 0.346 | 1.988 |
| *Nitrosospira multiformis ATCC 25196 chromosome 1* | NC_007614 | 0.190 | 4.441 |
| *Nocardia farcinica IFM 10152* | NC_006361 | 0.099 | 3.521 |
| *Novosphingobium aromaticivorans DSM 12444* | NC_007794 | 0.104 | 2.707 |
| *Onion yellows phytoplasma OY-M* | NC_005303 | 0.246 | 1.109 |
| *Pasteurella multocida subsp. multocida str. Pm70* | NC_002663 | 0.335 | 2.594 |
| *Polaromonas sp. JS666* | NC_007948 | 0.116 | 3.729 |
| *Porphyromonas gingivalis W83* | NC_002950 | 0.142 | 2.365 |
| *Prochlorococcus marinus str. MIT 9312* | NC_007577 | 0.205 | 5.124 |
| *Prochlorococcus marinus subsp. pastoris str. CCMP1986* | NC_005072 | 0.186 | 2.738 |
| *Pseudomonas aeruginosa PAO1* | NC_002516 | 0.187 | 1.777 |
| *Pseudomonas entomophila L48* | NC_008027 | 0.183 | 5.404 |
| *Pseudomonas fluorescens Pf-5* | NC_004129 | 0.225 | 3.590 |
| *Pseudomonas fluorescens PfO-1* | NC_007492 | 0.151 | 3.411 |
| *Pseudomonas putida KT2440* | NC_002947 | 0.179 | 3.059 |
| *Pseudomonas syringae pv. phaseolicola 1448A* | NC_005773 | 0.190 | 4.730 |
| *Pseudomonas syringae pv. syringae B728a* | NC_007005 | 0.175 | 5.196 |
| *Pseudomonas syringae pv. tomato str. DC3000* | NC_004578 | 0.154 | 3.781 |
| *Psychrobacter arcticus 273-4* | NC_007204 | 0.185 | 4.090 |
| *Psychrobacter cryohalolentis K5* | NC_007969 | 0.179 | 3.814 |
| *Ralstonia eutropha JMP134 chromosome 1* | NC_007347 | 0.139 | 2.366 |
| *Ralstonia metallidurans CH34 chromosome 1* | NC_007973 | 0.170 | 2.728 |
| *Ralstonia metallidurans CH34 chromosome 2* | NC_007974 | 0.168 | 2.187 |
| *Ralstonia solanacearum GMI1000* | NC_003295 | 0.161 | 2.262 |
| *Rhodoferax ferrireducens T118* | NC_007908 | 0.100 | 3.398 |
| *Rhodopirellula baltica SH 1* | NC_005027 | 0.209 | 3.403 |
| *Rhodopseudomonas palustris CGA009* | NC_005296 | 0.104 | 4.193 |
| *Rickettsia bellii RML369-C* | NC_007940 | 0.244 | 1.364 |
| *Rickettsia felis URRWXCal2* | NC_007109 | 0.330 | 2.184 |
| *Rubrobacter xylanophilus DSM 9941* | NC_008148 | 0.348 | 2.839 |
| *Salmonella enterica subsp. enterica serovar Typhi Ty2* | NC_004631 | 0.236 | 4.616 |
| *Salmonella typhimurium LT2* | NC_003197 | 0.236 | 5.299 |
| *Shigella flexneri 2a str. 2457T* | NC_004741 | 0.126 | 3.152 |
| *Shigella flexneri 2a str. 301* | NC_004337 | 0.129 | 2.898 |
| *Shigella sonnei Ss046* | NC_007384 | 0.137 | 4.265 |
| *Silicibacter pomeroyi DSS-3* | NC_003911 | 0.118 | 3.243 |
| *Silicibacter sp. TM1040* | NC_008044 | 0.183 | 5.321 |
| *Sodalis glossinidius str. 'morsitans'* | NC_007712 | 0.196 | 2.239 |
| *Streptomyces coelicolor A3(2)* | NC_003888 | 0.145 | 2.287 |
| *Synechococcus elongatus PCC 7942* | NC_007604 | 0.081 | 2.762 |
| *Syntrophus aciditrophicus SB* | NC_007759 | 0.232 | 2.620 |
| *Thermobifida fusca YX* | NC_007333 | 0.242 | 4.606 |
| *Thermus thermophilus HB27* | NC_005835 | 0.156 | 2.180 |
| *Thiomicrospira denitrificans ATCC 33889* | NC_007575 | 0.267 | 3.198 |
| *Treponema denticola ATCC 35405* | NC_002967 | 0.250 | 2.672 |
| *Vibrio cholerae O1 biovar eltor str. N16961 chromosome II* | NC_002506 | 0.328 | 2.367 |
| *Wigglesworthia glossinidia endosymbiont of Glossina brevipalpis* | NC_004344 | 0.282 | 4.427 |
| *Wolbachia endosymbiont of Drosophila melanogaster* | NC_002978 | 0.216 | 2.414 |
| *Wolbachia endosymbiont strain TRS of Brugia malayi* | NC_006833 | 0.351 | 1.130 |
| *Xanthomonas axonopodis pv. citri str. 306* | NC_003919 | 0.113 | 3.981 |
| *Xanthomonas campestris pv. campestris str. 8004* | NC_007086 | 0.113 | 3.527 |
| *Xanthomonas campestris pv. campestris str. ATCC 33913* | NC_003902 | 0.118 | 3.372 |
| *Xanthomonas campestris pv. vesicatoria str. 85-10* | NC_007508 | 0.117 | 4.221 |
| *Xanthomonas oryzae pv. oryzae KACC10331* | NC_006834 | 0.106 | 4.785 |
| *Xanthomonas oryzae pv. oryzae MAFF 311018* | NC_007705 | 0.108 | 2.750 |
| *Yersinia pestis biovar Microtus str. 91001* | NC_005810 | 0.201 | 4.689 |
| *Yersinia pestis CO92* | NC_003143 | 0.263 | 3.675 |
| *Yersinia pestis KIM* | NC_004088 | 0.269 | 3.973 |
| *Zymomonas mobilis subsp. mobilis ZM4* | NC_006526 | 0.292 | 1.015 |
| **Type C – Eukaryotes (71 chromosomes)** | **Accession no.** | ***χ_i_*,*_bg_*** | ***r_χ_*** |
| *A. fumigatus* | NC_007201 | 0.167 | 2.297 |
| *C. glabrata* | NC_005967 | 0.336 | 1.509 |
| *C. glabrata* | NC_005968 | 0.260 | 1.294 |
| *C. glabrata* | NC_006026 | 0.335 | 1.785 |
| *C. glabrata* | NC_006027 | 0.272 | 1.650 |
| *C. glabrata* | NC_006028 | 0.269 | 1.600 |
| *C. glabrata* | NC_006029 | 0.244 | 1.861 |
| *C. glabrata* | NC_006030 | 0.222 | 1.605 |
| *C. glabrata* | NC_006031 | 0.227 | 2.364 |
| *C. glabrata* | NC_006032 | 0.205 | 1.970 |
| *C. glabrata* | NC_006033 | 0.206 | 1.749 |
| *C. glabrata* | NC_006034 | 0.216 | 1.437 |
| *C. glabrata* | NC_006035 | 0.215 | 1.408 |
| *C. neoformans* | NC_006680 | 0.168 | 2.310 |
| *C. neoformans* | NC_006682 | 0.239 | 1.512 |
| *C. neoformans* | NC_006683 | 0.224 | 2.111 |
| *D. hansenii* | NC_006043 | 0.223 | 1.212 |
| *D. hansenii* | NC_006044 | 0.212 | 1.370 |
| *D. hansenii* | NC_006045 | 0.184 | 2.539 |
| *D. hansenii* | NC_006046 | 0.195 | 1.565 |
| *E. cuniculi* | NC_003230 | 0.322 | 1.881 |
| *E. cuniculi* | NC_003231 | 0.353 | 1.870 |
| *E. cuniculi* | NC_003232 | 0.340 | 1.016 |
| *E. cuniculi* | NC_003233 | 0.298 | 2.222 |
| *E. cuniculi* | NC_003234 | 0.314 | 1.065 |
| *E. cuniculi* | NC_003235 | 0.331 | 1.663 |
| *E. cuniculi* | NC_003236 | 0.313 | 3.786 |
| *E. cuniculi* | NC_003237 | 0.320 | 1.301 |
| *E. cuniculi* | NC_003238 | 0.231 | 1.709 |
| *E. gossypii* | NC_005782 | 0.246 | 1.278 |
| *E. gossypii* | NC_005783 | 0.247 | 1.234 |
| *E. gossypii* | NC_005784 | 0.193 | 1.561 |
| *E. gossypii* | NC_005786 | 0.163 | 1.943 |
| *K. lactis* | NC_006037 | 0.203 | 1.503 |
| *K. lactis* | NC_006038 | 0.181 | 1.593 |
| *S. cerevisiae* | NC_001134 | 0.244 | 1.198 |
| *S. cerevisiae* | NC_001136 | 0.188 | 1.474 |
| *S. cerevisiae* | NC_001137 | 0.335 | 1.460 |
| *S. cerevisiae* | NC_001139 | 0.217 | 1.921 |
| *S. cerevisiae* | NC_001140 | 0.286 | 1.532 |
| *S. cerevisiae* | NC_001141 | 0.330 | 2.469 |
| *S. cerevisiae* | NC_001142 | 0.289 | 1.800 |
| *S. cerevisiae* | NC_001143 | 0.254 | 1.480 |
| *S. cerevisiae* | NC_001144 | 0.256 | 2.088 |
| *S. cerevisiae* | NC_001145 | 0.227 | 1.970 |
| *S. cerevisiae* | NC_001146 | 0.260 | 2.129 |
| *S. cerevisiae* | NC_001147 | 0.200 | 1.256 |
| *S. cerevisiae* | NC_001148 | 0.197 | 1.757 |
| *S. pombe* | NC_003421 | 0.184 | 1.696 |
| *P. falciparum* | NC_004315 | 0.338 | 2.091 |
| *P. falciparum* | NC_004316 | 0.328 | 2.568 |
| *P. falciparum* | NC_004317 | 0.280 | 2.884 |
| *P. falciparum* | NC_004329 | 0.331 | 2.312 |
| *P. falciparum* | NC_004330 | 0.325 | 1.709 |
| *P. falciparum* | NC_004331 | 0.272 | 2.281 |
| *D. melanogaster* | NC_004353 | 0.283 | 2.044 |
| *T. castaneum* | Chr01 | 0.161 | 2.160 |
| *T. castaneum* | Chr06 | 0.186 | 2.023 |
| *T. castaneum* | Chr10 | 0.226 | 0.980 |
| *B. taurus* | Chr15 | 0.030 | 3.421 |
| *B. taurus* | Chr25 | 0.051 | 5.000 |
| *G. gallus* | Chr12 | 0.057 | 4.493 |
| *G. gallus* | Chr13 | 0.084 | 5.232 |
| *G. gallus* | Chr19 | 0.124 | 3.637 |
| *G. gallus* | Chr26 | 0.113 | 3.873 |
| *G. gallus* | ChrW | 0.097 | 3.099 |
| *C. familiaris* | Chr15 | 0.036 | 4.440 |
| *C. familiaris* | Chr19 | 0.044 | 6.126 |
| *C. familiaris* | Chr24 | 0.025 | 5.784 |
| *C. familiaris* | Chr27 | 0.035 | 3.682 |
| *M. musculus* | Chr05 | 0.013 | 3.706 |
| **Type D – Archaea (14 chromosomes)** | **Accession no.** | ***χ_i_*,*_bg_*** | ***r_χ_*** |
| *Aeropyrum pernix K1* | NC_000854 | 0.155 | 1.285 |
| *Archaeoglobus fulgidus DSM 4304* | NC_000917 | 0.097 | 1.761 |
| *Haloarcula marismortui ATCC 43049* | NC_006396 | 0.072 | 2.049 |
| *Methanocaldococcus jannaschii DSM 2661* | NC_000909 | 0.139 | 1.520 |
| *Methanopyrus kandleri AV19* | NC_003551 | 0.145 | 1.820 |
| *Methanosarcina acetivorans C2A* | NC_003552 | 0.116 | 2.063 |
| *Methanosarcina barkeri str. Fusaro* | NC_007355 | 0.090 | 1.447 |
| *Methanospirillum hungatei JF-1* | NC_007796 | 0.100 | 1.120 |
| *Methanothermobacter thermautotrophicus str. Delta H* | NC_000916 | 0.136 | 1.830 |
| *Natronomonas pharaonis DSM 2160* | NC_007426 | 0.090 | 2.049 |
| *Picrophilus torridus DSM 9790* | NC_005877 | 0.129 | 1.625 |
| *Pyrobaculum aerophilum str. IM2* | NC_003364 | 0.109 | 0.773 |
| *Sulfolobus tokodaii str. 7* | NC_003106 | 0.140 | 1.295 |
| *Thermococcus kodakarensis KOD1* | NC_006624 | 0.100 | 2.053 |
| **Type D – Eubacteria (41 chromosomes)** | **Accession no.** | ***χ_i_*,*_bg_*** | ***r_χ_*** |
| *Acidobacteria bacterium Ellin345* | NC_008009 | 0.069 | 2.770 |
| *Agrobacterium tumefaciens str. C58* | NC_003062 | 0.150 | 1.631 |
| *Agrobacterium tumefaciens str. C58* | NC_003304 | 0.150 | 1.631 |
| *Anabaena variabilis ATCC 29413* | NC_007413 | 0.061 | 0.891 |
| *Anaeromyxobacter dehalogenans 2CP-C* | NC_007760 | 0.129 | 1.583 |
| *Aquifex aeolicus VF5* | NC_000918 | 0.069 | 1.102 |
| *Bordetella pertussis Tohama I* | NC_002929 | 0.101 | 2.269 |
| *Burkholderia cenocepacia AU 1054 chromosome 3* | NC_008062 | 0.122 | 1.528 |
| *Burkholderia mallei ATCC 23344 chromosome 1* | NC_006348 | 0.085 | 1.174 |
| *Burkholderia pseudomallei K96243 chromosome 1* | NC_006350 | 0.117 | 2.171 |
| *Burkholderia thailandensis E264* | NC_007651 | 0.115 | 1.434 |
| *Caulobacter crescentus CB15* | NC_002696 | 0.102 | 2.330 |
| *Corynebacterium efficiens YS-314* | NC_004369 | 0.111 | 2.016 |
| *Deinococcus radiodurans R1* | NC_001263 | 0.095 | 2.026 |
| *Erythrobacter litoralis HTCC2594* | NC_007722 | 0.056 | 1.777 |
| *Gloeobacter violaceus PCC 7421* | NC_005125 | 0.051 | 1.150 |
| *Jannaschia sp. CCS1* | NC_007802 | 0.163 | 1.427 |
| *Mesorhizobium loti MAFF303099* | NC_002678 | 0.059 | 1.015 |
| *Mycobacterium avium subsp. paratuberculosis K-10* | NC_002944 | 0.082 | 2.043 |
| *Nostoc sp. PCC 7120* | NC_003272 | 0.059 | 1.343 |
| *Pseudoalteromonas haloplanktis TAC125 chromosome II* | NC_007482 | 0.189 | 0.843 |
| *Ralstonia eutropha JMP134* | NC_007348 | 0.116 | 1.719 |
| *Rhizobium etli CFN 42* | NC_007761 | 0.076 | 0.928 |
| *Rhodobacter sphaeroides 2.4.1 chromosome 1* | NC_007493 | 0.082 | 1.331 |
| *Rhodobacter sphaeroides 2.4.1 chromosome 2* | NC_007494 | 0.114 | 1.368 |
| *Rhodopseudomonas palustris BisB18* | NC_007925 | 0.068 | 2.386 |
| *Rhodopseudomonas palustris BisB5* | NC_007958 | 0.082 | 2.664 |
| *Rhodopseudomonas palustris HaA2* | NC_007778 | 0.073 | 2.464 |
| *Shigella boydii Sb227* | NC_007613 | 0.091 | 2.055 |
| *Shigella dysenteriae Sd197* | NC_007606 | 0.095 | 1.279 |
| *Sinorhizobium meliloti 1021* | NC_003047 | 0.071 | 1.714 |
| *Sphingopyxis alaskensis RB2256* | NC_008048 | 0.082 | 1.667 |
| *Streptomyces avermitilis MA-4680* | NC_003155 | 0.096 | 1.468 |
| *Synechococcus elongatus PCC 6301* | NC_006576 | 0.080 | 2.679 |
| *Synechococcus sp. JA-2-3B'a(2-13)* | NC_007776 | 0.045 | 1.118 |
| *Synechococcus sp. JA-3-3Ab* | NC_007775 | 0.042 | 1.187 |
| *Synechocystis sp. PCC 6803* | NC_000911 | 0.044 | 1.080 |
| *Thermosynechococcus elongatus BP-1* | NC_004113 | 0.061 | 1.514 |
| *Thermotoga maritima MSB8* | NC_000853 | 0.149 | 1.020 |
| *Thermus thermophilus HB8* | NC_006461 | 0.148 | 1.292 |
| *Thiobacillus denitrificans ATCC 25259* | NC_007404 | 0.082 | 2.088 |
| **Type D – Eukaryotes (251 chromosomes)** | **Accession no.** | ***χ_i_*,*_bg_*** | ***r_χ_*** |
| *A. fumigatus* | NC_007194 | 0.083 | 1.388 |
| *A. fumigatus* | NC_007195 | 0.089 | 1.276 |
| *A. fumigatus* | NC_007196 | 0.093 | 1.279 |
| *A. fumigatus* | NC_007197 | 0.117 | 2.068 |
| *A. fumigatus* | NC_007198 | 0.089 | 1.098 |
| *A. fumigatus* | NC_007199 | 0.111 | 1.695 |
| *A. fumigatus* | NC_007200 | 0.127 | 1.676 |
| *C. albicans* | NC_007436 | 0.148 | 1.663 |
| *C. glabrata* | NC_006036 | 0.168 | 1.613 |
| *C. neoformans* | NC_006670 | 0.118 | 1.820 |
| *C. neoformans* | NC_006679 | 0.159 | 1.821 |
| *C. neoformans* | NC_006681 | 0.152 | 1.395 |
| *C. neoformans* | NC_006684 | 0.154 | 1.550 |
| *C. neoformans* | NC_006685 | 0.119 | 1.783 |
| *C. neoformans* | NC_006686 | 0.120 | 1.365 |
| *C. neoformans* | NC_006687 | 0.153 | 1.482 |
| *C. neoformans* | NC_006691 | 0.142 | 1.589 |
| *C. neoformans* | NC_006692 | 0.149 | 1.074 |
| *C. neoformans* | NC_006693 | 0.151 | 1.554 |
| *C. neoformans* | NC_006694 | 0.155 | 1.421 |
| *D. hansenii* | NC_006047 | 0.157 | 1.335 |
| *D. hansenii* | NC_006048 | 0.157 | 1.185 |
| *D. hansenii* | NC_006049 | 0.171 | 1.099 |
| *E. gossypii* | NC_005785 | 0.147 | 1.582 |
| *E. gossypii* | NC_005787 | 0.159 | 1.670 |
| *E. gossypii* | NC_005788 | 0.158 | 1.532 |
| *K. lactis* | NC_006039 | 0.151 | 1.032 |
| *K. lactis* | NC_006040 | 0.174 | 1.544 |
| *K. lactis* | NC_006041 | 0.137 | 1.276 |
| *K. lactis* | NC_006042 | 0.116 | 1.560 |
| *S. pombe* | NC_003423 | 0.143 | 1.626 |
| *S. pombe* | NC_003424 | 0.126 | 1.820 |
| *Y. lipolytica* | NC_006067 | 0.129 | 1.635 |
| *Y. lipolytica* | NC_006068 | 0.106 | 1.219 |
| *Y. lipolytica* | NC_006069 | 0.108 | 1.958 |
| *Y. lipolytica* | NC_006070 | 0.092 | 1.255 |
| *Y. lipolytica* | NC_006071 | 0.089 | 1.489 |
| *Y. lipolytica* | NC_006072 | 0.087 | 1.314 |
| *A. thaliana* | NC_003070 | 0.049 | 1.160 |
| *A. thaliana* | NC_003071 | 0.044 | 0.869 |
| *A. thaliana* | NC_003074 | 0.042 | 1.160 |
| *A. thaliana* | NC_003075 | 0.056 | 1.086 |
| *A. thaliana* | NC_003076 | 0.040 | 0.850 |
| *O. sativa* | Chr01 | 0.028 | 1.234 |
| *O. sativa* | Chr02 | 0.027 | 1.354 |
| *O. sativa* | Chr03 | 0.029 | 1.288 |
| *O. sativa* | Chr04 | 0.034 | 1.269 |
| *O. sativa* | Chr05 | 0.039 | 1.077 |
| *O. sativa* | Chr06 | 0.040 | 1.659 |
| *O. sativa* | Chr07 | 0.038 | 0.918 |
| *O. sativa* | Chr08 | 0.041 | 1.251 |
| *O. sativa* | Chr09 | 0.047 | 0.943 |
| *O. sativa* | Chr10 | 0.047 | 1.180 |
| *O. sativa* | Chr11 | 0.041 | 1.289 |
| *O. sativa* | Chr12 | 0.043 | 1.521 |
| *A. gambiae* | NC_004818 | 0.032 | 1.245 |
| *A. gambiae* | NT_078265 | 0.024 | 1.344 |
| *A. gambiae* | NT_078266 | 0.019 | 1.060 |
| *A. gambiae* | NT_078267 | 0.027 | 1.072 |
| *A. gambiae* | NT_078268 | 0.025 | 0.948 |
| *C. elegans* | NC_003279 | 0.090 | 1.304 |
| *C. elegans* | NC_003280 | 0.075 | 1.139 |
| *C. elegans* | NC_003281 | 0.091 | 1.317 |
| *C. elegans* | NC_003282 | 0.073 | 0.971 |
| *C. elegans* | NC_003283 | 0.056 | 1.177 |
| *C. elegans* | NC_003284 | 0.085 | 2.523 |
| *D. melanogaster* | NC_004354 | 0.037 | 1.612 |
| *D. melanogaster* | NT_033777 | 0.021 | 0.745 |
| *D. melanogaster* | NT_033778 | 0.030 | 1.202 |
| *D. melanogaster* | NT_033779 | 0.029 | 1.400 |
| *D. melanogaster* | NT_037436 | 0.030 | 1.028 |
| *A. mellifera* | Chr01 | 0.026 | 1.440 |
| *A. mellifera* | Chr02 | 0.033 | 1.250 |
| *A. mellifera* | Chr03 | 0.031 | 1.020 |
| *A. mellifera* | Chr04 | 0.035 | 0.797 |
| *A. mellifera* | Chr05 | 0.036 | 1.613 |
| *A. mellifera* | Chr06 | 0.040 | 1.187 |
| *A. mellifera* | Chr07 | 0.047 | 1.533 |
| *A. mellifera* | Chr08 | 0.045 | 1.241 |
| *A. mellifera* | Chr09 | 0.038 | 1.333 |
| *A. mellifera* | Chr10 | 0.039 | 1.391 |
| *A. mellifera* | Chr11 | 0.038 | 1.259 |
| *A. mellifera* | Chr12 | 0.039 | 1.319 |
| *A. mellifera* | Chr13 | 0.041 | 2.089 |
| *A. mellifera* | Chr14 | 0.049 | 1.869 |
| *A. mellifera* | Chr15 | 0.051 | 1.668 |
| *A. mellifera* | Chr16 | 0.064 | 1.617 |
| *T. castaneum* | Chr02 | 0.126 | 2.072 |
| *T. castaneum* | Chr03 | 0.085 | 2.218 |
| *T. castaneum* | Chr04 | 0.102 | 2.020 |
| *T. castaneum* | Chr05 | 0.083 | 1.555 |
| *T. castaneum* | Chr07 | 0.094 | 1.847 |
| *T. castaneum* | Chr08 | 0.120 | 2.106 |
| *T. castaneum* | Chr09 | 0.128 | 1.733 |
| *D. rerio* | Chr01 | 0.026 | 1.284 |
| *D. rerio* | Chr02 | 0.035 | 0.788 |
| *D. rerio* | Chr03 | 0.031 | 2.023 |
| *D. rerio* | Chr04 | 0.036 | 1.692 |
| *D. rerio* | Chr05 | 0.022 | 0.879 |
| *D. rerio* | Chr06 | 0.031 | 1.414 |
| *D. rerio* | Chr07 | 0.023 | 1.258 |
| *D. rerio* | Chr08 | 0.034 | 1.085 |
| *D. rerio* | Chr09 | 0.025 | 1.008 |
| *D. rerio* | Chr10 | 0.028 | 1.524 |
| *D. rerio* | Chr11 | 0.031 | 1.335 |
| *D. rerio* | Chr12 | 0.024 | 1.498 |
| *D. rerio* | Chr13 | 0.027 | 1.811 |
| *D. rerio* | Chr14 | 0.024 | 1.590 |
| *D. rerio* | Chr15 | 0.035 | 0.798 |
| *D. rerio* | Chr16 | 0.023 | 1.823 |
| *D. rerio* | Chr17 | 0.037 | 2.028 |
| *D. rerio* | Chr18 | 0.028 | 1.943 |
| *D. rerio* | Chr19 | 0.023 | 1.205 |
| *D. rerio* | Chr20 | 0.027 | 1.242 |
| *D. rerio* | Chr21 | 0.029 | 1.128 |
| *D. rerio* | Chr22 | 0.030 | 1.183 |
| *D. rerio* | Chr23 | 0.029 | 1.077 |
| *D. rerio* | Chr24 | 0.028 | 1.070 |
| *D. rerio* | Chr25 | 0.036 | 1.761 |
| *B. taurus* | Chr01 | 0.018 | 0.752 |
| *B. taurus* | Chr02 | 0.023 | 2.523 |
| *B. taurus* | Chr03 | 0.016 | 1.026 |
| *B. taurus* | Chr04 | 0.027 | 2.152 |
| *B. taurus* | Chr05 | 0.026 | 2.304 |
| *B. taurus* | Chr06 | 0.028 | 2.344 |
| *B. taurus* | Chr07 | 0.028 | 1.431 |
| *B. taurus* | Chr08 | 0.023 | 2.782 |
| *B. taurus* | Chr09 | 0.021 | 1.442 |
| *B. taurus* | Chr10 | 0.028 | 0.991 |
| *B. taurus* | Chr11 | 0.017 | 1.050 |
| *B. taurus* | Chr12 | 0.020 | 1.697 |
| *B. taurus* | Chr13 | 0.027 | 0.784 |
| *B. taurus* | Chr14 | 0.028 | 1.916 |
| *B. taurus* | Chr16 | 0.032 | 0.837 |
| *B. taurus* | Chr17 | 0.030 | 2.160 |
| *B. taurus* | Chr18 | 0.041 | 2.332 |
| *B. taurus* | Chr19 | 0.024 | 2.264 |
| *B. taurus* | Chr20 | 0.033 | 1.518 |
| *B. taurus* | Chr21 | 0.037 | 1.180 |
| *B. taurus* | Chr22 | 0.050 | 2.306 |
| *B. taurus* | Chr23 | 0.027 | 1.199 |
| *B. taurus* | Chr24 | 0.019 | 2.529 |
| *B. taurus* | Chr26 | 0.030 | 2.860 |
| *B. taurus* | Chr27 | 0.038 | 1.582 |
| *B. taurus* | Chr28 | 0.037 | 1.506 |
| *B. taurus* | Chr29 | 0.041 | 2.096 |
| *B. taurus* | ChrX | 0.038 | 1.862 |
| *G. gallus* | Chr01 | 0.015 | 0.734 |
| *G. gallus* | Chr02 | 0.015 | 1.026 |
| *G. gallus* | Chr03 | 0.023 | 0.834 |
| *G. gallus* | Chr04 | 0.028 | 1.603 |
| *G. gallus* | Chr05 | 0.042 | 1.130 |
| *G. gallus* | Chr06 | 0.055 | 2.011 |
| *G. gallus* | Chr07 | 0.029 | 2.422 |
| *G. gallus* | Chr08 | 0.062 | 1.053 |
| *G. gallus* | Chr09 | 0.053 | 0.781 |
| *G. gallus* | Chr10 | 0.044 | 0.648 |
| *G. gallus* | Chr11 | 0.067 | 0.583 |
| *G. gallus* | Chr14 | 0.067 | 2.086 |
| *G. gallus* | Chr15 | 0.054 | 2.810 |
| *G. gallus* | Chr16 | 0.144 | 1.089 |
| *G. gallus* | Chr17 | 0.105 | 1.764 |
| *G. gallus* | Chr18 | 0.087 | 2.575 |
| *G. gallus* | Chr20 | 0.046 | 1.178 |
| *G. gallus* | Chr21 | 0.138 | 0.932 |
| *G. gallus* | Chr22 | 0.187 | 1.020 |
| *G. gallus* | Chr23 | 0.082 | 2.855 |
| *G. gallus* | Chr24 | 0.143 | 0.968 |
| *G. gallus* | Chr25 | 0.139 | 2.159 |
| *G. gallus* | Chr27 | 0.109 | 1.095 |
| *G. gallus* | Chr28 | 0.054 | 2.466 |
| *G. gallus* | ChrZ | 0.026 | 1.763 |
| *C. familiaris* | Chr01 | 0.015 | 0.776 |
| *C. familiaris* | Chr02 | 0.025 | 3.014 |
| *C. familiaris* | Chr03 | 0.019 | 1.157 |
| *C. familiaris* | Chr04 | 0.023 | 1.674 |
| *C. familiaris* | Chr05 | 0.023 | 0.843 |
| *C. familiaris* | Chr06 | 0.027 | 1.398 |
| *C. familiaris* | Chr07 | 0.025 | 1.072 |
| *C. familiaris* | Chr08 | 0.030 | 0.713 |
| *C. familiaris* | Chr09 | 0.019 | 1.064 |
| *C. familiaris* | Chr10 | 0.020 | 2.000 |
| *C. familiaris* | Chr11 | 0.031 | 1.485 |
| *C. familiaris* | Chr12 | 0.022 | 2.153 |
| *C. familiaris* | Chr13 | 0.036 | 1.593 |
| *C. familiaris* | Chr14 | 0.022 | 1.228 |
| *C. familiaris* | Chr16 | 0.037 | 0.823 |
| *C. familiaris* | Chr17 | 0.027 | 1.857 |
| *C. familiaris* | Chr18 | 0.038 | 2.580 |
| *C. familiaris* | Chr20 | 0.037 | 1.615 |
| *C. familiaris* | Chr21 | 0.042 | 0.910 |
| *C. familiaris* | Chr22 | 0.028 | 1.149 |
| *C. familiaris* | Chr23 | 0.031 | 0.960 |
| *C. familiaris* | Chr25 | 0.028 | 2.473 |
| *C. familiaris* | Chr26 | 0.042 | 2.151 |
| *C. familiaris* | Chr28 | 0.030 | 1.271 |
| *C. familiaris* | Chr29 | 0.032 | 2.416 |
| *C. familiaris* | Chr30 | 0.039 | 2.337 |
| *C. familiaris* | Chr31 | 0.026 | 2.610 |
| *C. familiaris* | Chr32 | 0.037 | 2.092 |
| *C. familiaris* | Chr33 | 0.047 | 1.551 |
| *C. familiaris* | Chr34 | 0.037 | 1.073 |
| *C. familiaris* | Chr35 | 0.049 | 1.750 |
| *C. familiaris* | Chr36 | 0.036 | 1.147 |
| *C. familiaris* | Chr37 | 0.047 | 1.431 |
| *C. familiaris* | Chr38 | 0.062 | 2.556 |
| *C. familiaris* | ChrX | 0.015 | 1.799 |
| *M. musculus* | Chr01 | 0.011 | 1.176 |
| *M. musculus* | Chr02 | 0.008 | 0.971 |
| *M. musculus* | Chr03 | 0.010 | 0.710 |
| *M. musculus* | Chr04 | 0.012 | 1.188 |
| *M. musculus* | Chr06 | 0.016 | 2.579 |
| *M. musculus* | Chr07 | 0.017 | 0.773 |
| *M. musculus* | Chr08 | 0.013 | 1.829 |
| *M. musculus* | Chr09 | 0.012 | 1.493 |
| *M. musculus* | Chr10 | 0.016 | 1.014 |
| *M. musculus* | Chr11 | 0.013 | 1.141 |
| *M. musculus* | Chr12 | 0.024 | 0.588 |
| *M. musculus* | Chr13 | 0.014 | 0.850 |
| *M. musculus* | Chr14 | 0.013 | 1.691 |
| *M. musculus* | Chr15 | 0.022 | 1.829 |
| *M. musculus* | Chr16 | 0.019 | 2.404 |
| *M. musculus* | Chr17 | 0.022 | 1.653 |
| *M. musculus* | Chr18 | 0.018 | 1.726 |
| *M. musculus* | Chr19 | 0.036 | 1.204 |
| *M. musculus* | ChrX | 0.019 | 1.952 |
| *M. musculus* | ChrY | 0.042 | 2.610 |
| *H. sapiens* | Chr01 | 0.012 | 1.715 |
| *H. sapiens* | Chr02 | 0.008 | 0.894 |
| *H. sapiens* | Chr03 | 0.012 | 1.985 |
| *H. sapiens* | Chr04 | 0.014 | 2.294 |
| *H. sapiens* | Chr05 | 0.012 | 1.018 |
| *H. sapiens* | Chr06 | 0.014 | 2.932 |
| *H. sapiens* | Chr07 | 0.013 | 1.448 |
| *H. sapiens* | Chr08 | 0.018 | 2.316 |
| *H. sapiens* | Chr09 | 0.016 | 2.376 |
| *H. sapiens* | Chr10 | 0.014 | 2.161 |
| *H. sapiens* | Chr11 | 0.016 | 2.539 |
| *H. sapiens* | Chr12 | 0.014 | 2.599 |
| *H. sapiens* | Chr13 | 0.019 | 1.414 |
| *H. sapiens* | Chr14 | 0.034 | 1.097 |
| *H. sapiens* | Chr15 | 0.025 | 2.654 |
| *H. sapiens* | Chr16 | 0.020 | 0.943 |
| *H. sapiens* | Chr17 | 0.021 | 1.817 |
| *H. sapiens* | Chr18 | 0.021 | 1.918 |
| *H. sapiens* | Chr19 | 0.023 | 1.211 |
| *H. sapiens* | Chr20 | 0.025 | 0.576 |
| *H. sapiens* | Chr21 | 0.048 | 1.223 |
| *H. sapiens* | Chr22 | 0.040 | 2.177 |
| *H. sapiens* | ChrX | 0.014 | 1.376 |
| *H. sapiens* | ChrY | 0.056 | 0.909 |
