# Supplementary material for: Inverse Symmetry in Complete Genomes and Whole-Genome Inverse Duplication
Source: PLoS One. 2009 Nov 9;4(11):e7553. doi: 10.1371/journal.pone.0007553 (PMC2771390; doi:10.1371/journal.pone.0007553)
Supplement: Table S3 — List of 38 exceptional chromosomes and their χi values. (0.09 MB DOC) [file pone.0007553.s003.doc]

Table S3. List of 38 exceptional chromosomes and their *χ_i_* values

| **Bacteria chromosome (23 chromosomes)** | **Accession no.** | ***L* (Mb)** | ***χ_i_*** | **Type** |
| --- | --- | --- | --- | --- |
| *X. fastidiosa 9a5c* | NC_002488 | 2.68 | 0.517 | A |
| *C. Blochmannia floridanus* | NC_005061 | 0.71 | 0.353 | A |
| *A. phagocytophilum HZ* | NC_007797 | 1.47 | 0.328 | A |
| *S. epidermidis RP62A* | NC_002976 | 2.62 | 0.304 | A |
| *C. Blochmannia pennsylvanicus str. BPEN* | NC_007292 | 0.79 | 0.295 | A |
| *E. chaffeensis str. Arkansas* | NC_007799 | 1.18 | 0.278 | A |
| *C. perfringens str. 13* | NC_003366 | 3.03 | 0.267 | A |
| *S. epidermidis ATCC 12228* | NC_004461 | 2.50 | 0.239 | A |
| *B. aphidicola str. Bp* | NC_004545 | 0.62 | 0.225 | A |
| *B. henselae str. Houston-1* | NC_005956 | 1.93 | 0.211 | A |
| *P. marinus str. MIT 9313* | NC_005071 | 2.41 | 0.340 | B |
| *D. hafniense Y51* | NC_007907 | 5.73 | 0.307 | B |
| *H. ducreyi 35000HP* | NC_002940 | 1.70 | 0.250 | B |
| *B. aphidicola str. Sg* | NC_004061 | 0.64 | 0.222 | B |
| *F. nucleatum subsp. nucleatum ATCC 25586* | NC_003454 | 2.17 | 0.206 | B |
| *B. aphidicola str. APS* | NC_002528 | 0.64 | 0.202 | B |
| *W. endosymbiont strain TRS of Brugia malayi* | NC_006833 | 1.08 | 0.311 | C |
| *Z. mobilis subsp. mobilis ZM4* | NC_006526 | 2.06 | 0.279 | C |
| *I. loihiensis L2TR* | NC_006512 | 2.84 | 0.277 | C |
| *D. radiodurans R1* | NC_001264 | 0.41 | 0.246 | C |
| *M. synoviae 53* | NC_007294 | 0.80 | 0.240 | C |
| *M. synoviae 53* | NC_005303 | 0.86 | 0.230 | C |
| *P. haloplanktis TAC125* | NC_007482 | 0.64 | 0.225 | D |
| **Eukaryotes chromosome (15 chromosomes)** | **Accession no.** | ***L* (Mb)** | ***χ_i_*** | **Type** |
| *P. falciparum* | NC_004318 | 1.20 | 0.376 | B |
| *S. cerevisiae* | NC_001135 | 0.32 | 0.352 | B |
| *P. falciparum* | NC_004325 | 0.64 | 0.348 | B |
| *P. falciparum* | NC_004314 | 1.69 | 0.304 | B |
| *P. falciparum* | NC_004328 | 1.35 | 0.276 | B |
| *S. cerevisiae* | NC_001133 | 0.23 | 0.265 | B |
| *P. falciparum* | NC_004326 | 1.34 | 0.250 | B |
| *P. falciparum* | NC_004327 | 1.42 | 0.231 | B |
| *S. cerevisiae* | NC_001138 | 0.27 | 0.207 | B |
| *E. cuniculi* | NC_003232 | 0.21 | 0.335 | C |
| *E. cuniculi* | NC_003234 | 0.23 | 0.294 | C |
| *E. cuniculi* | NC_003237 | 0.27 | 0.245 | C |
| *T. castaneum* | Chr10 | 5.79 | 0.233 | C |
| *S. cerevisiae* | NC_001137 | 0.58 | 0.227 | C |
| *C. glabrata* | NC_005967 | 0.49 | 0.220 | C |
