# Supplementary figures and images for: Inverse Symmetry in Complete Genomes and Whole-Genome Inverse Duplication
Source: PLoS One. 2009 Nov 9;4(11):e7553. doi: 10.1371/journal.pone.0007553 (PMC2771390; doi:10.1371/journal.pone.0007553)

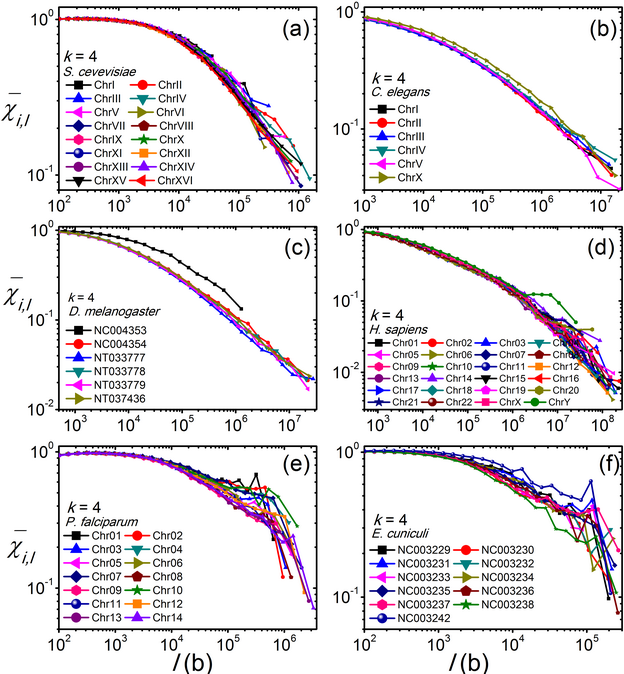

Supplement: Figure S1 — χi,l-l plots for six eukaryotes (number of chromosomes in parentheses). (a) Yeast (16), (b) Worm (6), (c) Fly (6), (d) Human (24), (e) P. falciparum(14), (f) E. cuniculi (11). In each case the result for all chromosomes are overlayed. Results for other k-mers are similar. (1.26 MB TIF) [file pone.0007553.s004.tif]

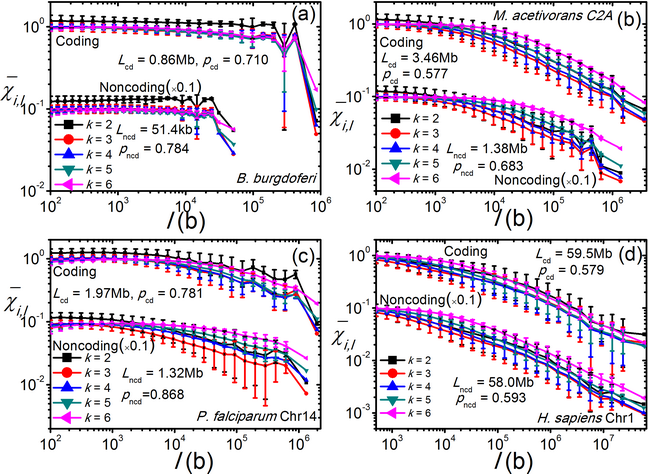

Supplement: Figure S2 — χi,l-l plots for the coding and non-coding parts of (a) the type-A eubacterial B. burgdorferi (5% of chromosome is non-coding), (b) the type-D archaeon M. acetivorans (29%), (c) the type-C chromosome 14 of the protozoan P. falciparum (41%), and (d) the type-D chromosome 1 of human (49%). (0.92 MB TIF) [file pone.0007553.s005.tif]

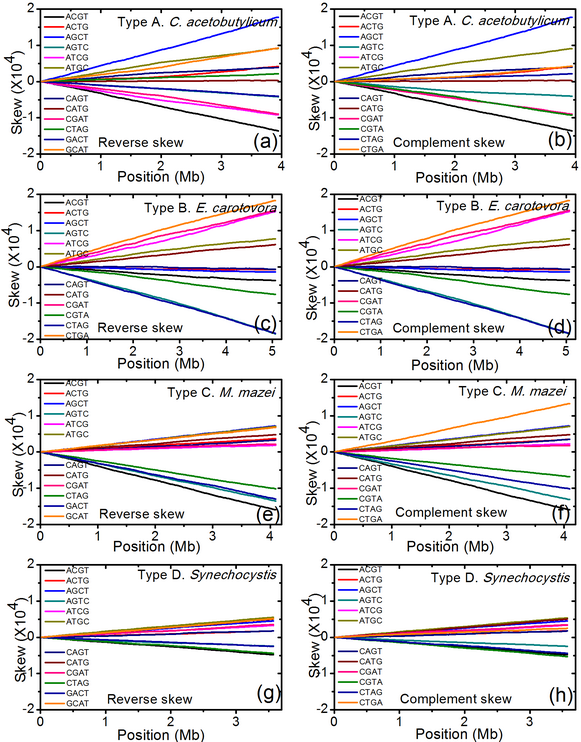

Supplement: Figure S3 — Cumulative skews of 12 base-neutral reverse- and complement-conjugate paris of 4-mers in four types of chromosomes. Reverse (a) and complement (b) skews in type-A C. acetobutylicum; reverse (c) and complement (d) skews in type-B E. carotovora; reverse (e) and complement (f) skews in type-C M. mazei; reverse (g) and complement (h) skews in type-D Synechocystis. (1.29 MB TIF) [file pone.0007553.s006.tif]
